# Total synthesis of asperdinones B, C, D, E and terezine D

**DOI:** 10.3762/bjoc.21.210

**Published:** 2025-12-17

**Authors:** Ravi Devarajappa, Stephen Hanessian

**Affiliations:** 1 Department of Chemistry, Université de Montréal, Station Centre-Ville, C.P. 6128, Montreal, QC, H3C 3J7, Canada; 2 Department of Pharmaceutical Sciences, University of California, Irvine, CA, 92697, USA

**Keywords:** C–H activation, indole alkaloid, Negishi reaction, prenylation

## Abstract

The total synthesis of new members of prenylated indole alkaloids exhibiting α-glucosidase activity is described. Asperdinones B, C, D, and E are characterized by the presence of a (3*R*)-3-indolylmethylbenzodiazepine-2,5-dione unit at C-3 of C4–C7 prenylated indoles. Methods of direct and indirect prenylation of indole and tryptophan were explored. Different approaches were adopted for the functionalization of C4–C7 prenylindoles at C-3 using Negishi cross-coupling methods. The asperdinones are among the rare tryptophan-derived indole alkaloids which appear to have undergone epimerization due to genetic alteration of specific gene clusters that code for a (3*R*) configuration.

## Introduction

Alkaloids constitute an important family of naturally occurring compounds with a rich history in the annals of bioactive compounds [1]. Among these, the family of indole alkaloids is known for its biomedical importance [2]. These alkaloids have been isolated from plants [3], marine sources [4], bacterial sources [5–7], and they are particularly relevant primarily because of their potent pharmacological activities as among other, anticancer drugs, but also for their architecturally intricate structures [8]. For these reasons, and considering the structural and stereochemical complexities of some members, indole alkaloids have been prime compounds of interest for total synthesis with spectacular successes [2,9–10]. A subset of simple indole alkaloids contains a prenyl group at various positions in the indole ring [11]. The importance of the indole core structure and the nature and position of prenylation is reflected by the observation that 6-prenylindole but not 6-isopropylindole has been reported to exhibit antifungal activity [6]. Tryptophans containing a prenyl group at the 5, 6, or 7 positions are found as naturally occurring metabolites from diverse plant and bacterial sources [5–7]. The biosynthesis of prenyltryptophans is well studied and involves a series of prenyl transferases [12–13].

**Figure 1:**
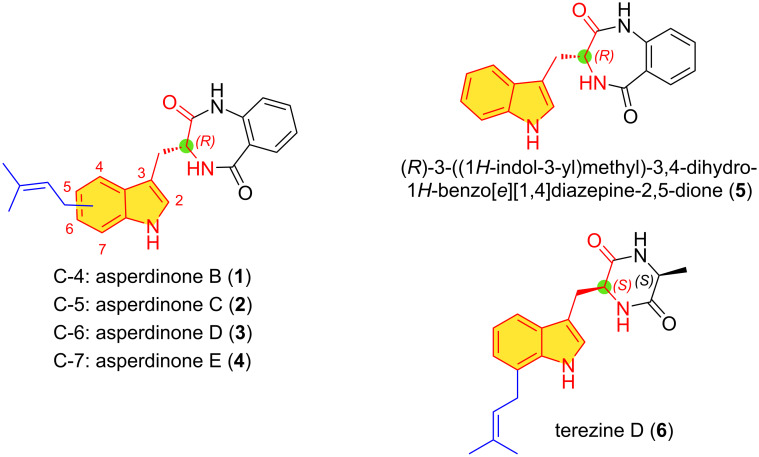
Structures of prenylindole alkaloids derived from tryptophan.

A series of 4-, 5-, 6-, and 7-prenylated 3-indolylmethylbenzodiazepine-2,5-diones known as asperdinones B, C, D, and E (**1**–**4**) exhibiting moderate α-glucosidase inhibitory activity was recently isolated from cultures of *Aspergillus spinosus* WHUF0344 (Figure 1) [14]. The putative biogenetic precursor, (3*R*)-3-indolylmethylbenzodiazepine-2,5-dione **5** was also isolated as a metabolite. Historically, (3*S*)-3-indolylbenzodiazepine-2,5-dione *ent*-**5** was isolated for the first time as a natural metabolite from the fungal culture extract of *Aspergillus flavipes* by Barrow and Sun [15] and synthesized by the condensation of isatoic anhydride and ʟ-tryptophan as previously reported by Bock in 1987 [16]. It is of interest that although the biosynthesis of 3-indolylbenzoquinone-2,5-dione *ent*-**5** is initiated with ʟ-tryptophan and anthranilic acid [17], the resulting natural products **1**–**4** possess a (3*R*) configuration. This is because of an epimerization mediated by the non-ribosomal peptide synthetase AnaPS within the genetic machinery of these microorganisms during biosynthesis [18–19]. Subsequently, post-translational enzymatic processes mediated by prenyl transferases (AnaPT) lead to prenylation at the C4–C7 sites in the indole unit.

Terezine D (**6**), an indole-containing metabolite and cyclic dipeptide consisting of a 7-prenylated ʟ-tryptophan and ʟ-alanine was isolated from the liquid cultures of *S. teretispora* containing potato broth in the medium. Its structure was based on extensive spectroscopic studies (Figure 1). Terezine D (**6**) exhibited modest in vitro antifungal activity [20]. A related metabolite lacking the C7 prenyl group had been isolated from the cultures of *Aspergillus chevalieri* in 1976 [21]. The structure and absolute stereochemistry were confirmed by synthesis from tryptophan and ʟ-alanine [22]. The isolation of terezine D (**6**) and more recently, the (3*R*)-configured asperdinones as new members of this small subfamily of 7-prenyltryptophans with appended benzodiazepine-2,5-dione and diketopiperazine units piqued our interest. Herein, we report our efforts toward the total synthesis of asperdinones B, D, C, and E (**1**–**4**) and terezine D (**6**).

**Figure 2:**
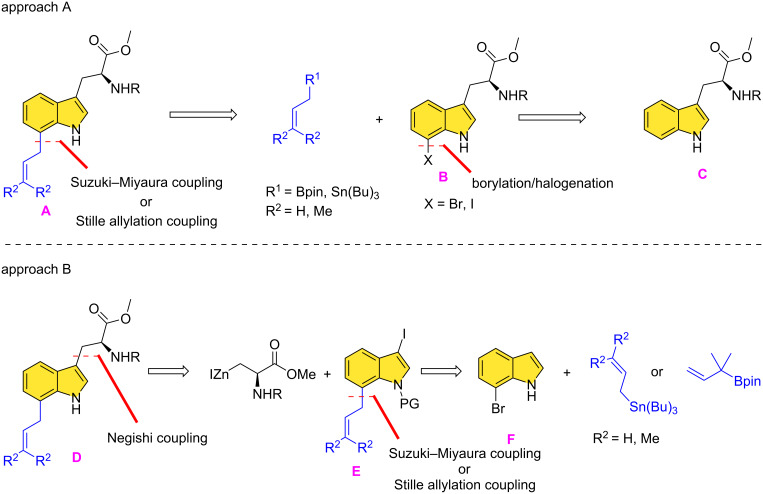
Representative retrosynthetic considerations for 7-prenyl- and 7-allyltryptophan.

Considering their relatively simple structures, we considered two basic approaches to 4-, 5-, 6-, and 7-prenylated tryptophans as synthetic precursors which could be converted to the intended natural products by cyclization to benzodiazepine-2,5-diones or diketopiperazines. First, is the evident use of tryptophan as a starting chiral synthon (chiron), and to regioselectively install a prenyl group at different sites on the indole moiety (Figure 2, approach A, **A–C**) [23–24]. This would formally mimic the post-translational biosynthetic pathway wherein a prenyl group would be inserted at C4–C7 selectively via the prenyl transferase AnaPT. A second, less evident approach, starts with a prenylindole, followed by introducing an (*R*)- or (*S*)*-*2-aminopropionate (alanine) unit at C-3 by a Negishi cross-coupling reaction (Figure 2, approach B, **D–F**). Although each approach has precedents in different contexts, achieving regioselective bond formation and functional group compatibility presented unforeseen challenges.

Methods for the chemical synthesis of prenyltryptophans (approach A, **A–C**) are scarce [25–26]. Adopting a bio-inspired approach, Ishikawa et al. treated tryptophan ethyl ester with prenyl alcohol in the presence of 2 equivalents of H_2_SO_4_ in water to obtain a mixture of six C-prenylated tryptophans from which the 7-prenyl isomer could be isolated in 4% yield after chromatography on a 10 g scale [27]. This direct prenylation method was then adapted for the synthesis of terezine D (**6**), which was isolated as a pale-yellow amorphous powder. Viswanathan [25] and Chen [28] reported a C-2 prenylation of tryptophan methyl ester mediated by acid salts and Lewis acids, respectively. A prenylation at C-4 in bis-*N*-Boc-tryptophan methyl ester has been achieved by Chein utilizing optimized Suzuki coupling conditions [29].

## Results and Discussion

**Scheme 1:**
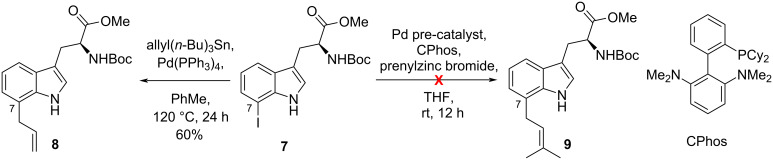
C-7 Functionalization of 7-iodo-*N*^α^-Boc-tryptophan methyl ester.

Adopting approach A, we considered prenylation of the known methyl (2*S*)-((*tert*-butoxycarbonyl)amino)-3-(7-iodo-1*H*-indol-3-yl)propanoate (**7**) [30] with prenylzinc bromide using a catalytic system reported by Buchwald. In that study, Buchwald’s group achieved the C-6 prenylation of 6-bromoindole in 94% yield using CPhos-Pd-G3 as the palladium pre-catalyst (Scheme 1) [31]. However, under the same reported conditions only the unreacted starting material was recovered. In an isolated example, C-7 allylation of **7** was reported via a Stille coupling protocol to give **8** (Scheme 1) [32].

**Scheme 2:**
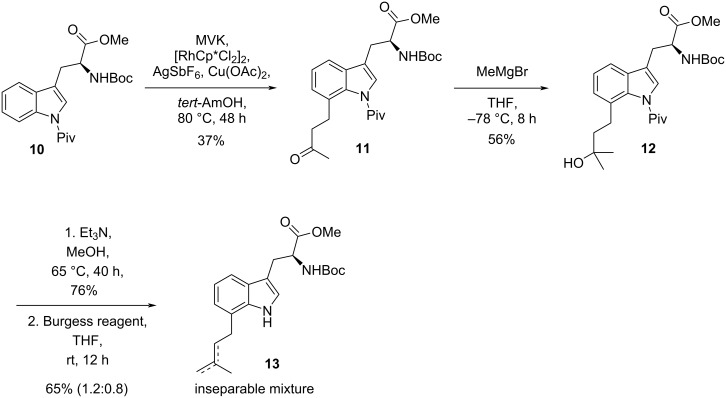
C-7 Prenylation via C–H activation.

As an alternative approach to C-7 functionalization of *N*^α^-Boc-tryptophan methyl ester, we chose a CH activation protocol [33]. Treatment of *N*^1^-Piv-*N*^α^-Boc-tryptophan methyl ester (**10**) with but-3-en-2-one (MVK) in the presence of [RhCp*Cl_2_]_2_ catalyst according to Ma et al. [34] led to the 7-(3-keto-1-butyl)-alkylated product **11** in 37% yield (Scheme 2). Despite the modest yield, product **11** was transformed to the corresponding tertiary alcohol **12**. Removal of the *N*-Piv group and dehydration with the Burgess reagent [35] led to an inseparable mixture of olefins slightly in favor of the *exo*-olefin isomer. Dehydration in the presence of the *N*-pivaloyl group led to the same mixture of isomers. Numerous conditions to change the ratio were not successful [36] (Supporting Information File 1, Table S1).

**Figure 3:**
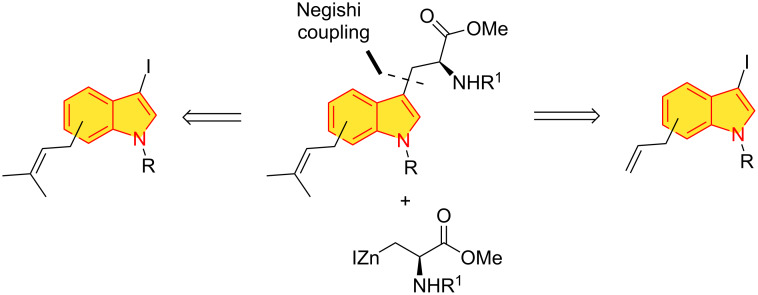
Negishi cross-coupling of allyl- and prenyl(iodo)indoles.

In view of the above mentioned issues, we considered chemically more challenging protocols starting with allyl- or prenylindoles (Figure 1, approach B) which would rely on a Negishi cross-coupling reaction [37–38] using Jackson’s iodozinc *N*-Boc-ʟ-serine methyl ester [39] and 3-iodoallyl- or 3-iodoprenylindoles (Figure 3). Related Negishi cross-coupling reactions have been reported for bromoindoles [40] and 3-iodo-*N*-Boc-7-methylindole [41] in excellent yields. The Negishi cross-coupling reaction with the organozinc reagent prepared from iodoalanine derivative (vide infra) has been used to prepare various aryl-substituted tryptophans in 76–96% yields [42]. In principle, this protocol should be applicable to all the C4–C7-substituted indoles as well as to the corresponding allylindoles. In the latter case, a Grubbs olefin cross-metathesis reaction with 2-butene would indirectly introduce the prenyl appendage [43–45].

The synthesis of 7-prenylindole from 2-iodoaniline in 4 steps was reported in 1996 [46–47]. Subsequently, the Pirrung group reported two practical syntheses of 7-prenylindole [48–49]. The regioselective C-7 prenylation of indole has been achieved by directing-group C–H activation as reported by Snieckus [50]. However, the deprotection of the preferred *N*-bis-*tert*-butylphosphinoyl directing group required conditions that would be incompatible with the presence of an amino acid appendage at C-3. 7-Prenylindole has been prepared from *N*-Boc-indoline in the presence of *sec*-BuLi, TMEDA, and prenyl bromide at −78 °C, followed by oxidation with MnO_2_ [51].

**Scheme 3:**
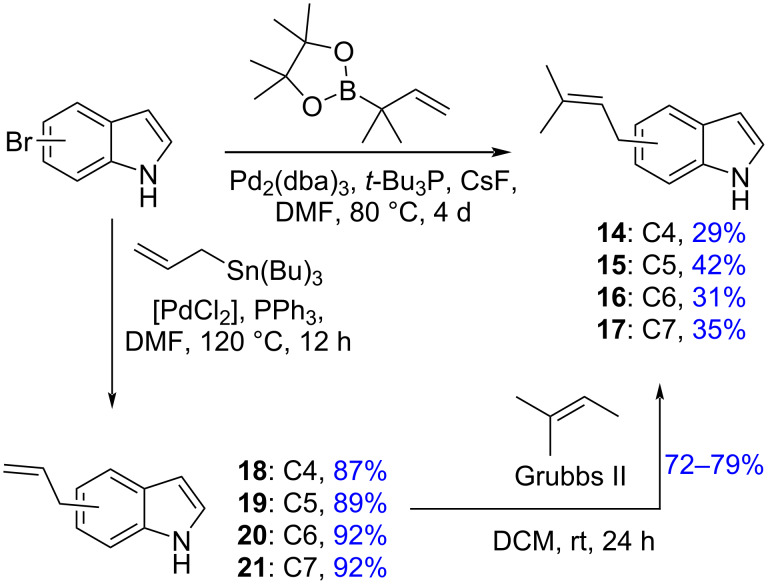
Synthesis of prenyl- and allylindoles.

We deemed it necessary to explore alternative synthetic methods to prenyl- and allylindoles as starting points toward the synthesis of asperdinones B, C, D, and E (**1**–**4**) as well as to terezine D (**6**). To access 4-prenylindole, we adopted a Pd-catalyzed Suzuki coupling of 3,3-dimethyl-1-butene-3-pinacol boronate with bromoindoles [52]. Unfortunately, despite the relatively simple protocol (Pd(PPh_3_)_4_, toluene, NaOH, 90 °C, 12 h) and the reported high yield of 4-prenylindole, we consistently obtained inseparable mixtures of the desired prenylindole and the reverse indole products. Mixtures of prenylated compounds have been observed under the same conditions with 4-*tert*-butylbromobenzene [53]. We therefore adopted a prenylation method used by Knölker for bromocarbazole derivatives using Pd_2_(dba)_3_ [54]. Pleasingly, this led to the corresponding prenylindoles **14**–**17** without forming the isomeric reverse prenylated adducts, although the starting material was recovered intact resulting in modest yields of the coupling products (Scheme 3). It is of interest that 5-, 6-, and 7-prenylindoles are found as naturally occurring metabolites from diverse plant sources [5–7].

The synthesis of allylindoles **18**–**21** was realized in excellent yields via a Stille coupling of the corresponding bromoindoles (Scheme 3) [43,55]. Grubbs cross-coupling [56] afforded the corresponding prenylindoles **14**–**17** in excellent yields.

**Scheme 4:**
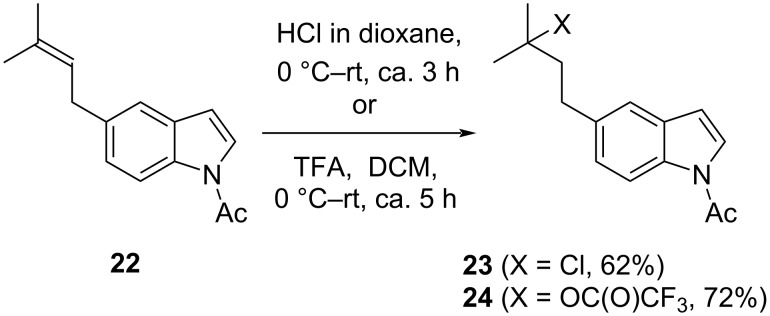
Markovnikov hydrochlorination and hydrotrifluoroacetylation.

Prior to initiating a synthesis toward our intended target molecules **1**–**4** (Figure 1), we tested the stability of prenylindoles under acidic conditions. Not surprisingly, treatment of *N*-acetyl-5-prenyl-1*H*-indole (**22**) as a test substrate with HCl in dioxane or TFA in dichloromethane led to Markovnikov hydrochlorination and hydrotrifluoroacetylation of the prenyl group, respectively (Scheme 4) [57–58].

This result required the use of an N-protecting group in the iodozinc amino acid reagent that could be removed under non-acidic conditions after the cross-coupling reaction. To this end, we prepared iodo *N*-Fmoc-ᴅ-alanyl anthranilamide methyl ester **29** from ᴅ-serine.

**Scheme 5:**
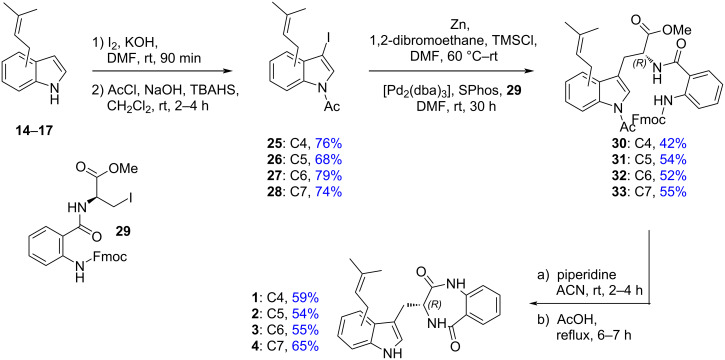
Synthesis of asperdinones B–E **1**–**4**.

Treatment of prenylindoles **14**–**17** with iodine and KOH followed by acetylation afforded the corresponding iodoindoles **25**–**28** in 68–79% yields (Scheme 5). Negishi cross-coupling according to Jackson et al. [39] with the organozinc reagent prepared from **29** gave the adducts **30**–**33** in 42–55% yields. Removal of the Fmoc and *N*-acetyl groups with piperidine gave the corresponding anthranilamides, which upon heating with acetic acid [59] afforded asperdinones **1**–**4** in an average overall yield of 14% via the allylation/cross metathesis route and 7% via the direct prenylation route. In all cases, spectroscopic and analytical data were in accordance with the literature.

**Scheme 6:**
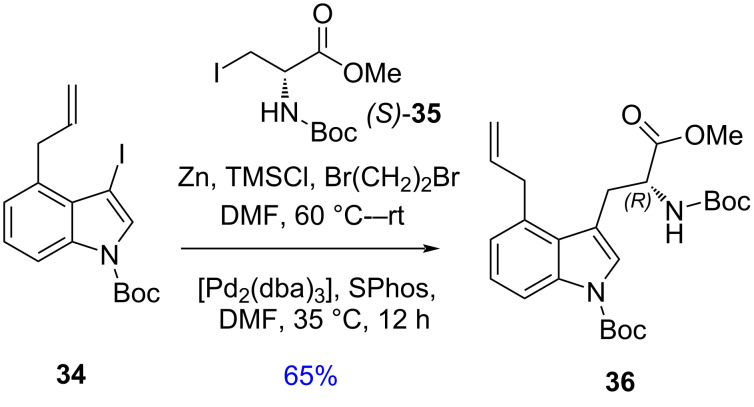
Control experiment.

The moderate yield in the Negishi cross-coupling reactions was attributed in part to the nature of the organozinc reagent **29**. In contrast, a similar coupling using the organozinc reagent prepared from iodo *N*-Boc-ᴅ-alanyl methyl ester (*S*)-**35** with 4-allylindole under the same conditions afforded a significantly improved yield of 65%. Steric bulk due to the presence of the allyl or propenyl group at different positions does not appear to affect the yields (Scheme 6).

**Scheme 7:**
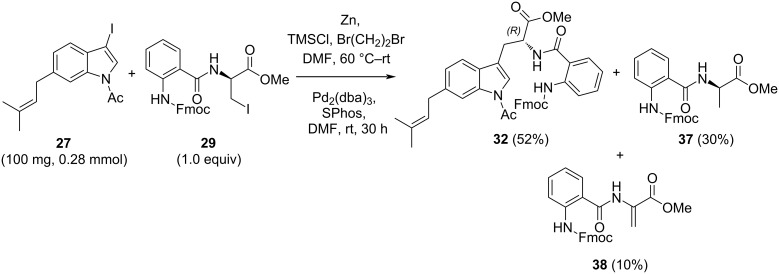
Control experiment of the Negishi cross-coupling reaction.

Importantly, it was observed that reduction and β-elimination of the organozinc reagent prepared from **29** took place during the cross-coupling reaction, thereby affecting the yield (Scheme 7) [60]. For example, using 1 equivalent of reagent **29**, the cross-coupling of 3-iodo-6-prenyl-*N*-acetylindole (**27**) led to the adduct **32** in 52% yield accompanied by the reduced product **37** in 30% and the dehydro product **38** in 10% yield. Control experiments revealed that the iodozinc reagent from **29** was unaffected in the presence of the Pd catalyst. It follows that elimination and reduction must occur after Pd insertion and formation of a pallado–zinc intermediate which undergoes β-elimination and proton transfer. Seminal studies by Jackson [61] have reported related results with iodozinc *N*-Boc-ʟ-alanine methyl ester who observed intramolecular proton transfer with partial incorporation of deuterium upon quenching with deuterium oxide. In a different context, the role of the Pd catalyst and the associated ligand was studied in the cross-coupling of the organozinc reagent *ent*-**35** with 3-iodomethylfuran resulting in a large variation in ratios of *N*-Boc-alanine methyl ester coupled products to dehydro-*N*-Boc- and reduced *N*-Boc-alanines [60].

**Scheme 8:**
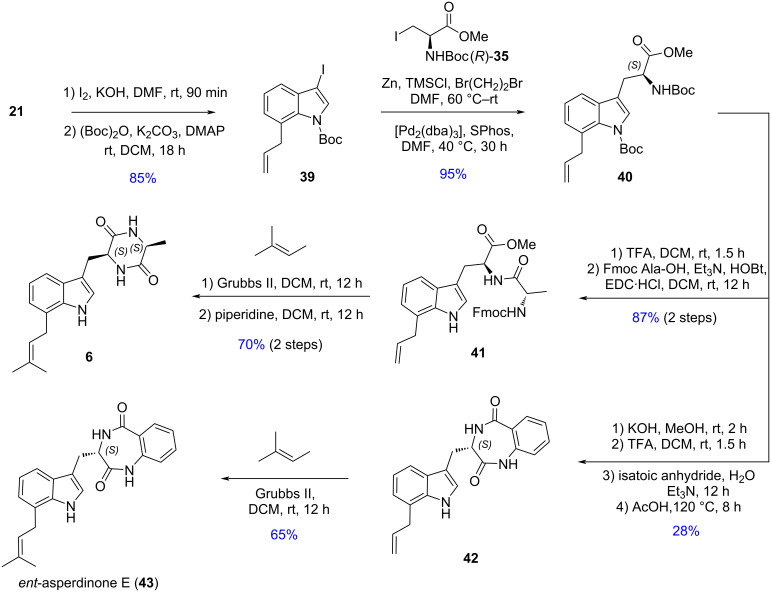
Synthesis of terezine D and *ent*-asperdinone E.

The modest yields starting with the prenylation of the respective bromoindoles urged us to explore the use of allylindoles as precursors for the synthesis of terezine D (**6**) and *ent*-asperdinone E, now using an *N*-Boc protecting group (Scheme 8). Treatment of 3-iodoindole **39** with the iodozinc reagent prepared from *N*-Boc-ʟ-alanine methyl ester ((*R)*-**35**) under Negishi coupling conditions afforded **40** in excellent yield. Cleavage of the *N*-Boc group and amide formation with Fmoc-ʟ-alanine gave **41** which was subjected to a cross-metathesis reaction with the Grubbs II catalyst, then deprotection to give terezine D (**6**) in 44% overall yield. Application of the same protocol using isatoic anhydride led to *ent*-asperdinone E (**43**) in 13% overall yield. It is interesting that the Negishi cross-coupling reactions took place in excellent yield with the iodozinc *N*-Boc reagent **35**, in contrast to the organozinc reagent derived from iodo *N*-Fmoc-ᴅ-alanyl anthranilamide methyl ester **29**.

## Conclusion

In summary, we have accomplished the total synthesis of asperdinones **1**–**4** and terezine D (**6**), members of a new class of prenylated 3-indolyl-2,5-benzodiazepine-1,4-diones and diketopiperazine alkaloids, respectively, through Negishi cross-coupling and related C–H functionalization strategies. Importantly, no epimerization was observed during the Negishi coupling or throughout the subsequent steps. Asperidinones **1**–**4** were obtained in 7% average yields via the direct prenylation route, and 14% from the allylation/cross-metathesis route, whereas terezine D (**6**) was isolated in 44% overall yield. Insights into the reactivity and stability of the iodozinc *N*-Fmoc-ᴅ-alanyl anthranilamide reagent derived from **29** were explored and validated. In view of the prevalent occurrence of prenylindoles, it is interesting to speculate whether there is an alternative biochemical pathway that involves prenylindoles as biogenetic precursors to prenylated tryptophans. The reverse appears to occur in the biosynthesis of 6-prenylindole-3-carbaldehyde (6-DMAI-3-carbaldehyde) via a gene cluster that contains a tryptophanase [62]. It is tempting to speculate whether prenylated indole-3-carbaldehyde could be enzymatically transformed to the corresponding prenylated tryptophans via a biogenetic Strecker-like process.

## Supporting Information

File 1 Experimental section and characterization of compounds.

## Data Availability

All data that supports the findings of this study is available in the published article and/or the supporting information of this article.
