# Supplementary material for: Total synthesis of asperdinones B, C, D, E and terezine D
Source: Beilstein J Org Chem. 2025 Dec 17;21:2730–8. doi: 10.3762/bjoc.21.210 (PMC12719882; doi:10.3762/bjoc.21.210)
Supplement: File 1 — Experimental section and characterization of compounds. [file Beilstein_J_Org_Chem-21-2730-s001.pdf]

BEILSTEIN JOURNAL OF ORGANIC CHEMISTRY

Supporting Information

for

Total synthesis of asperdinones B, C, D, E and terezine D

Ravi Devarajappa and Stephen Hanessian

Beilstein J. Org. Chem. **2025,** *21,* 2730–2738. doi:10.3762/bjoc.21.210

Experimental section and characterization of compounds

License and Terms: This is a supporting information file under the terms of the Creative Commons Attribution License (https://creativecommons.org/licenses/by/4.0). Please note that the reuse, redistribution and reproduction in particular requires that the author(s) and source are credited and that individual graphics may be subject to special legal provisions.
The license is subject to the *Beilstein Journal of Organic Chemistry* terms and conditions: (https://www.beilstein-journals.org/bjoc/terms)

Table of contents

Contents	Page No.
1. General information	S2
2. Experimental	S3
3. Characterization data of all compounds	S7
4. References	S25
5. ^{1}H NMR and ^{13}C NMR spectra	S26

1. General information

Physical data and spectroscopic measurements

NMR spectra were recorded on a Bruker AVANCETM 400 RG spectrometer (400 MHz for ^{1}H, 101 MHz for ^{13}C, 377 MHz for ^{19}F) and a Bruker AVANCETM 500 Ultra shield Plus spectrometer (500 MHz for ^{1}H, 126 MHz for ^{13}C and 471 MHz for ^{19}F) in chloroform-*d*, DMSO-d_6, acetone-d_6 or methanol-d_4. Spectra were taken in the indicated solvent at ambient temperature. Chemical shifts (δ) are given in parts per million (ppm) with tetramethyl silane as an internal standard. Multiplicities are recorded as follows: s = singlet, d = doublet, t = triplet, q = quartet, br = broad. Coupling constants (*J* values) are given in Hz. Accurate mass measurements were performed on a LC-TOF instrument from Agilent Technologies in positive electrospray mode. Protonated molecular ions $[M + H]^+$ and/or sodium adducts $[M + Na]^+$ were used for empirical formula confirmation. Infrared spectra (IR) were obtained on a PERKIN-ELMER Spectrum One ATR-FTIR system and are reported in terms of frequency of absorption (cm^{-1}). Melting points were determined on a Büchi B-540 melting point apparatus. Thin-layer chromatography (TLC) was performed on pre-coated Silicycle silica gel (250 μM, 60 Å) plates with F-254 indicator. Visualizing was performed with a UV light (254 nm) or with stains ($KMnO_4$, *p*-anisaldehyde, CAM or ninhydrin). ZEO prep 60 (0.040–0.063 mm) silica gel was used for all column chromatography.

All reactions involving air or moisture-sensitive compounds were performed under argon atmosphere in flame-dried glassware. Other solvents used for reactions were purified according to standard procedures. Starting reagents were purchased from commercial suppliers and used without further purification unless otherwise specified.

2. Experimental

General procedure A: prenylation

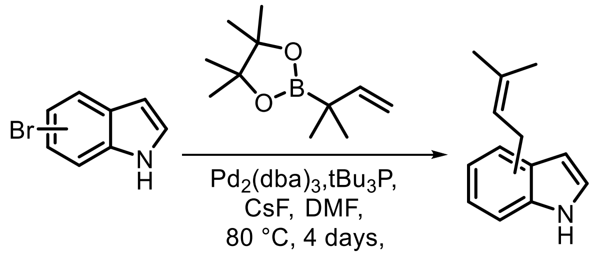

To a solution of bromoindole in dry DMF, Pd(dba)$_2$ (11.3 mg, 19.7 µmol) and P(*t*-Bu)$_3$ (8.0 mg, 40 µmol) was added, and the mixture was stirred at room temperature under an argon atmosphere for 5 min. To this solution, 3,3-dimethyl-1-butene-3-pinacol boronate (44.4 mg, 226 µmol) and CsF (16.2 mg, 107 µmol) were added, and the reaction mixture was slowly heated to 80 °C and stirred under argon for 4 days. The mixture was diluted with ethyl acetate, poured into water, and the layers were separated. The aqueous layer was extracted with ethyl acetate (2 × 20 mL). The combined organic layers were dried over Na_2SO_4 and purified by column chromatography on silica gel (hexanes/ethyl acetate 9:1) to afford the prenylated indole.

<u>General procedure B: allylation</u>

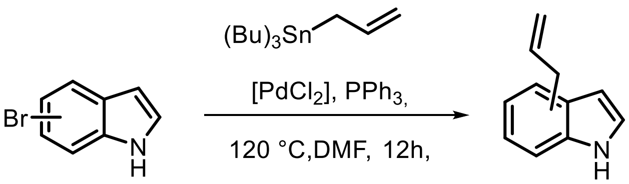

To a solution of bromoindole (1.0 g, 5.1 mmol, 1.0 equiv) in degassed DMF (12 mL) in a sealed tube were added triphenylphosphine (0.2 equiv), $PdCl_2$ (0.05 equiv), and allyltributylstannane (1.2 equiv). The bright yellow reaction mixture was heated to 120 °C in a preheated oil bath. After 16 h, the mixture was cooled to rt, and diethyl ether (25 mL) and saturated aqueous NaCl (15 mL) were added sequentially. The organic layer was separated, washed with water (15 mL), dried over Na_2SO_4, and concentrated under reduced pressure. The resulting yellow oil was purified by flash column chromatography on silica gel (hexanes/EtOAc 10:1) to afford the desired product as a colourless liquid in 91% yield.

<u>General procedure C: iodination and acetyl protection</u>

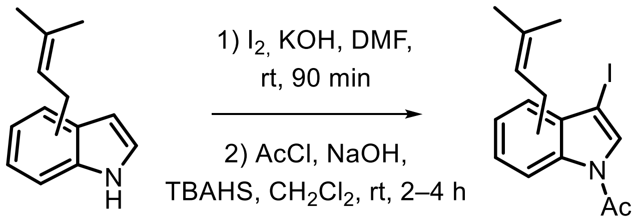

To a solution of prenylindole (284 mg, 1.53 mmol) in DMF (12 mL) was added powdered KOH (2.5 equiv). A solution of I_2 (1.0 equiv) in DMF (8 mL) was added dropwise to the stirring solution over 30 min. The reaction mixture was stirred for an additional 1 h, then poured into ice–water containing 0.5% NH_3 and 0.1% $Na_2S_2O_5$. The mixture was extracted with a 1:1 mixture of EtOAc/hexanes until no product remained in the aqueous phase. The combined organic layers were washed with cold water, dried over anhydrous Na_2SO_4, and concentrated in vacuo. The crude product was dissolved in CH_2Cl_2 (10 mL), and powdered NaOH (2.5 equiv) and tetrabutylammonium hydrogen sulfate (TBAHS, 20 mol %) were added. Acetyl chloride (1.5 equiv) in CH_2Cl_2 (6 mL) was then added dropwise to the vigorously stirred solution. After 2–4 h at rt, TLC indicated complete consumption of the prenylindole. The crude reaction mixture was purified by flash column chromatography on silica gel (hexanes/EtOAc 8:1) to afford the desired product.

General procedure D: Negishi coupling

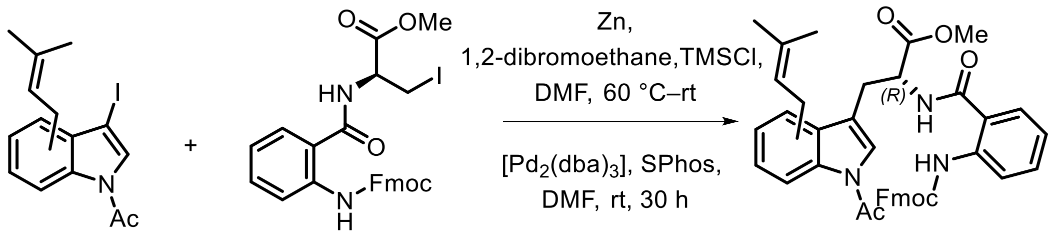

An oven-dried two-necked round-bottomed flask was further flame-dried twice under vacuum. After cooling to room temperature, Zn dust (132 mg, 1.98 mmol) was added under an argon atmosphere. The flask was evacuated and backfilled with argon three times. Dry DMF (6 mL) was then added via syringe, followed by 1,2-dibromoethane (0.6 equiv) to the stirred suspension. The mixture was stirred at 60 °C in an oil bath for 45 min, then cooled to rt. Chlorotrimethylsilane (0.2 equiv) was added via syringe, and the mixture was stirred for 40 min at rt. A solution of methyl (*S*)-2-(2-((((9H-fluoren-9-yl)methoxy)carbonyl)amino)benzamido)-3-iodopropanoate (1.50 equiv) in dry DMF (3 mL) was added via syringe to the activated zinc suspension at rt, and the mixture was stirred at 35 °C for 1 h. The reaction mixture was then cooled to rt, and under an argon atmosphere, $Pd_2(dba)_3$ (2.5 mol %) and 2-dicyclohexylphospino-2',6'-dimethoxybiphenyl (SPhos, 5 mol %) were added. A solution of the corresponding prenylated *N*-acetyl-3-iodo-1*H*-indole (0.25 mmol, 1.0 equiv) in DMF (1 mL) was immediately added, and the mixture was stirred at rt for 30 h. The reaction mixture was filtered through a pad of Celite, diluted with EtOAc, washed with H_2O and brine, dried over anhydrous Na_2SO_4, filtered, and concentrated under reduced pressure. The product was purified by silica gel chromatography.

General procedure E: deprotection and cyclization

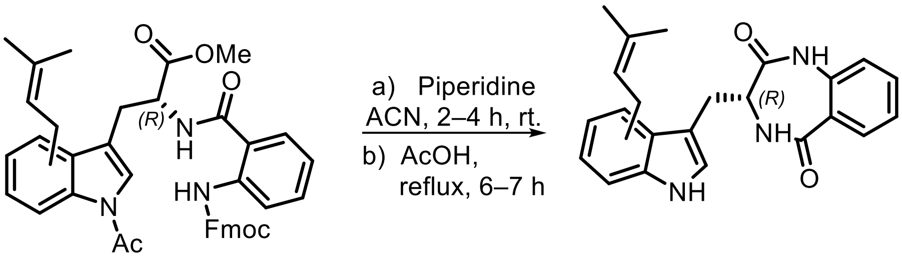

The prenylated methyl *N*-(2-((((9*H*-fluoren-9-yl)methoxy)carbonyl)amino)benzoyl)-1-acetyl-ᴅ-tryptophanate was dissolved in acetonitrile/piperidine (8:2, 2 mL), and the reaction mixture was stirred at rt until completion (2–4 h). The solvent was evaporated under reduced pressure, and the crude product was dissolved in AcOH (2 mL) in a sealed tube. The tube was heated at 120 °C for 6–7 h. After completion, the solvent was evaporated under reduced pressure, and the crude residue was purified by silica gel chromatography to afford the desired product.

General procedure F: olefin metathesis

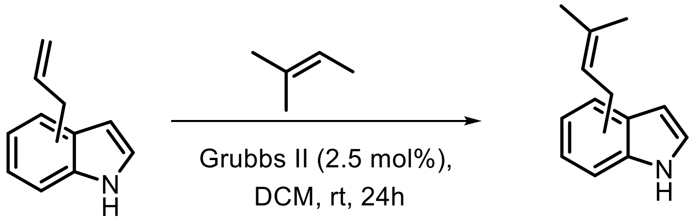

The allylindole (202 mg, 1.28 mmol) was dissolved in dry CH_2Cl_2 (2.0 mL). To this stirring solution, Grubbs II catalyst (21.1 mg, 2 mol %) and 2-methyl-2-butene (1.5 mL) were added. The reaction mixture was stirred at rt for 24 h. After completion, the crude mixture was filtered through a short pad of Celite, the solvent was removed under reduced pressure, and the residue was purified by silica gel chromatography to afford the desired product.

Table S1:

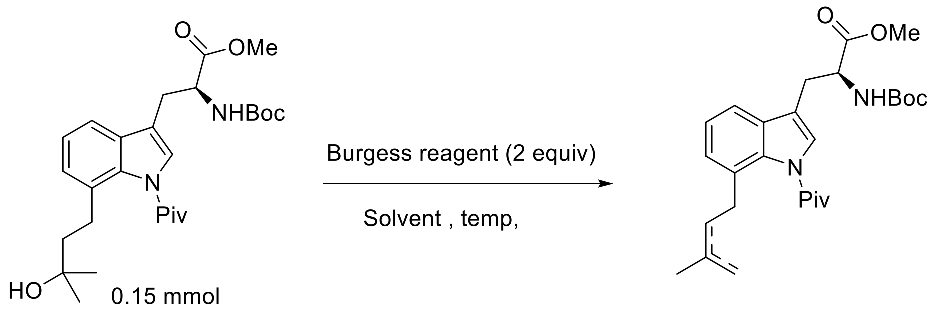

Entry	Solvent (2 ml)	Time (h)	Temp.	Yield (%)/Ratio
1	THF	6	0	69/1:2
2	THF	12	rt	85/1:2
3	1,4-dioxane	12	rt	trace
4	1,4-dioxane	12	reflux	trace
5	DCM	36	rt	52/1:2
6	Ethyl acetate	12	rt / reflux	NR

NR : no reaction

3. Characterization data:

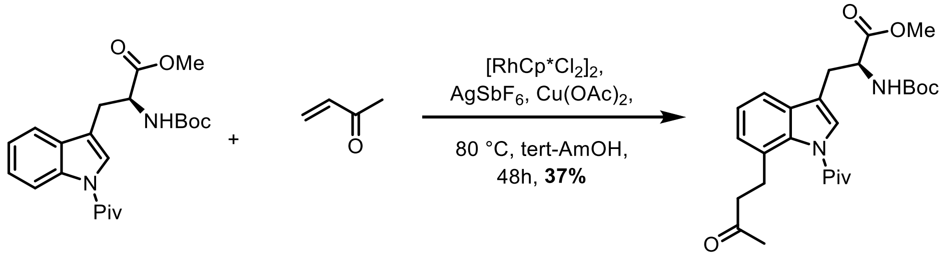

Methyl (*S*)-2-((*tert*-butoxycarbonyl) amino)-3-(7-(3-oxobutyl)-1-pivaloyl-1*H*-indol-3-yl)propanoate (11): To a dry sealed tube, methyl *N*-(tert-butoxycarbonyl)-1-pivaloyl-L-tryptophanate (0.2 mmol), $[RhCp^*Cl_2]_2$ (4 mol %), $Cu(OAc)_2 \cdot H_2O$ (0.41 mmol), and $AgSbF_6$ (16 mol %) were added. The tube was evacuated and backfilled with argon before acrylate (5.0 equiv) and *t*-AmylOH (2.0 mL) were added. The reaction mixture was stirred at 80 °C for 48 h. The cooled mixture was diluted with ethyl acetate, filtered through a short pad of Celite, concentrated under vacuum, and the residue was purified by chromatography on silica gel (hexanes/ethyl acetate) to afford methyl (*S*)-2-((*tert*-butoxycarbonyl)amino)-3-(7-(3-oxobutyl)-1-pivaloyl-1*H*-indol-3-yl)propanoate as a colourless liquid in 37% yield (brsm 90%). ^{1}H NMR (400 MHz, chloroform-*d*) δ 7.38 (d, *J* = 16.1 Hz, 2H), 7.24 (t, *J* = 7.6 Hz, 1H), 7.18 – 7.12 (m, 1H), 5.13 (d, *J* = 8.3 Hz, 1H), 4.73 (d, *J* = 7.6 Hz, 1H), 3.72 (s, 3H), 3.33 – 3.13 (m, 2H), 2.99 (dd, *J* = 8.7, 6.7 Hz, 2H), 2.82 (dd, *J* = 8.8, 6.6 Hz, 2H), 2.16 (s, 3H), 1.54 (s, 9H), 1.45 (s, 9H). ^{13}C NMR (101 MHz, $CDCl_3$) δ 208.4, 179.0, 172.5, 155.2, 139.8, 136.9, 135.6, 131.6, 129.0, 126.6, 124.3, 123.7, 116.9, 115.3, 80.2, 53.4, 52.5, 43.6, 41.9, 29.9, 29.3, 28.4, 28.3, 28.0. HRMS $(M+H)^+$ calcd. for $C_{26}H_{36}N_2O_6$ 473.2652, found 473.2649.

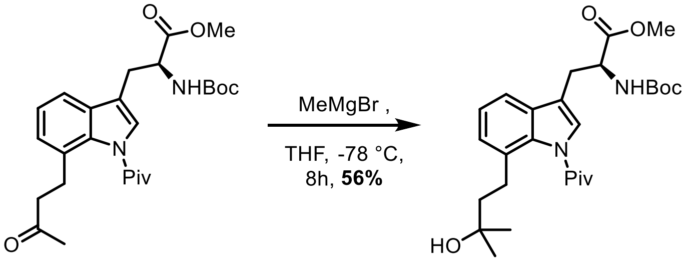

Methyl (*S*)-2-((*tert*-butoxycarbonyl) amino)-3-(7-(3-hydroxy-3-methylbutyl)-1-pivaloyl-1*H*-indol-3-yl)propanoate (12): To a dry round-bottomed flask, methyl (*S*)-2-((*tert*-butoxycarbonyl)amino)-3-(7-(3-oxobutyl)-1-pivaloyl-1*H*-indol-3-yl)propanoate (166.0 mg, 0.35 mmol, 1.0 equiv) and dry THF (7.0 mL) were added under an argon atmosphere. The reaction mixture was cooled to −78 °C, and a 3.0 M solution of MeMgBr in Et_2O (3.0 equiv) was added dropwise. The reaction was maintained at this temperature and monitored by TLC. After 8 h, the reaction mixture was slowly quenched with saturated aqueous NH_4Cl and extracted with ethyl acetate (3 ×). The combined organic layers were washed with brine, dried over Na_2SO_4, filtered, and concentrated in vacuo. The crude mixture was purified by flash chromatography to afford methyl (*S*)-2-((*tert*-butoxycarbonyl)amino)-3-(7-(3-hydroxy-3-methylbutyl)-1-pivaloyl-1*H*-indol-3-

yl)propanoate as a liquid in 56% yield.^{1}H NMR (400 MHz, chloroform-*d*) δ 7.40 (s, 1H), 7.33 (d, *J* = 7.6 Hz, 1H), 7.23 (t, *J* = 7.5 Hz, 1H), 7.17 (dd, *J* = 7.4, 1.4 Hz, 1H), 5.19 – 5.03 (m, 1H), 4.70 (t, *J* = 7.1 Hz, 1H), 3.70 (s, 3H), 3.30 – 3.08 (m, 2H), 2.83 – 2.75 (m, 2H), 1.87 (s, 1H), 1.84 – 1.77 (m, 2H), 1.53 (s, 9H), 1.43 (s, 9H), 1.26 (s, 6H). ^{13}C NMR (101 MHz, $CDCl_3$) δ 179.0, 172.5, 155.2, 135.6, 131.6, 130.6, 127.0, 124.3, 123.8, 116.5, 115.5, 80.1, 70.6, 53.4, 52.5, 43.6, 41.8, 29.5, 29.4, 29.3, 29.1, 28.4, 27.9. HRMS (M+H)$^+$ calcd. for $C_{27}H_{40}N_2O_6$ 489.2965, found 489.2961.

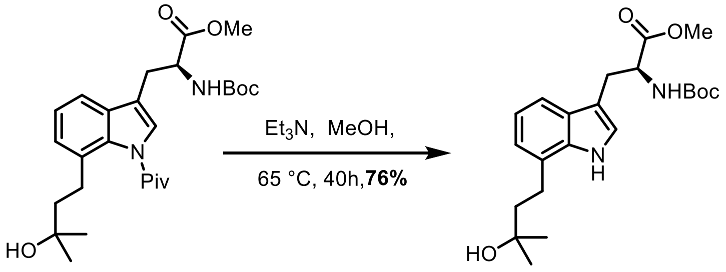

Methyl (*S*)-2-((*tert*-butoxycarbonyl)amino)-3-(7-(3-hydroxy-3-methylbutyl)-1*H*-indol-3-yl) propanoate: To a dry sealed tube, methyl (*S*)-2-((*tert*-butoxycarbonyl)amino)-3-(7-(3-hydroxy-3-methylbutyl)-1-pivaloyl-1*H*-indol-3-yl)propanoate (45 mg, 0.08 mmol) and MeOH (2.0 mL) were added. To this stirring solution, triethylamine (10 equiv) was added at rt. The sealed tube was then heated at 65 °C for 40 h. The solvent was evaporated, and the crude mixture was purified by column chromatography on silica gel (hexanes/ethyl acetate) to afford methyl (*S*)-2-((*tert*-butoxycarbonyl)amino)-3-(7-(3-hydroxy-3-methylbutyl)-1*H*-indol-3-yl)propanoate as a liquid in 76% yield.^{1}H NMR (400 MHz, chloroform-*d*) δ 8.99 (s, 1H), 7.40 (d, *J* = 7.8 Hz, 1H), 7.03 (t, *J* = 7.5 Hz, 1H), 7.00 – 6.94 (m, 2H), 5.09 (d, *J* = 8.2 Hz, 1H), 4.64 (q, *J* = 6.5, 6.1 Hz, 1H), 3.68 (s, 3H), 3.33 – 3.22 (m, 2H), 2.96 – 2.88 (m, 2H), 1.92 – 1.84 (m, 2H), 1.78 (s, 1H), 1.42 (s, 9H), 1.33 (s, 6H). ^{13}C NMR (101 MHz, $CDCl_3$) δ 172.9, 155.3, 135.1, 127.6, 125.6, 122.6, 121.3, 119.6, 116.6, 110.2, 79.8, 71.6, 54.2, 52.2, 42.8, 29.6, 28.3, 28.1, 25.8. HRMS (M+H)$^+$ calcd. for $C_{22}H_{32}N_2O_5$ 405.2389, found 405.2386.

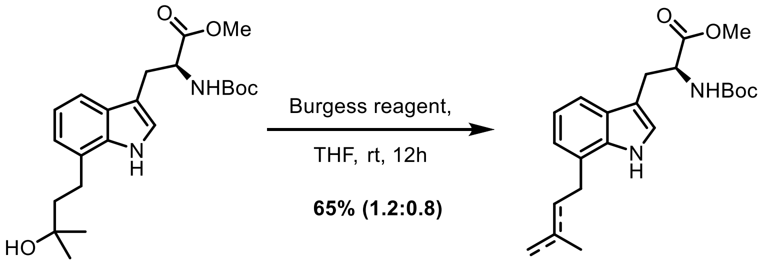

inseparable mixture

Methyl (*S*)-2-((*tert*-butoxycarbonyl)amino)-3-(7-(3-methylbut-2-en-1-yl)-1*H*-indol-3-yl)propanoate and **methyl (*S*)-2-((*tert*-butoxycarbonyl)amino)-3-(7-(3-methylbut-3-en-1-yl)-1*H*-indol-3-yl)propanoate (13):** In a dry round-bottomed flask, methyl (*S*)-2-((*tert*-butoxycarbonyl)amino)-3-(7-(3-hydroxy-3-methylbutyl)-1*H*-indol-3-yl)propanoate (13.3 mg, 0.0329 mmol) and dry THF (1.0 mL) were

added. To this stirring solution, Burgess reagent (2.0 equiv) was added at rt, and the reaction mixture was stirred at the same temperature for 12 h. The crude product was purified by column chromatography on silica gel (hexanes/ethyl acetate) to afford an inseparable mixture of dehydrated products in 65% yield.^{1}H NMR (400 MHz, chloroform-*d*) δ 8.06 (s, 1H), 7.41 (d, *J* = 7.7 Hz, 1H), 7.11 – 6.96 (m, 3H), 5.38 – 5.42 (m, 1H, endo), 5.14 – 5.02 (m, 1H), 4.84 – 4.76 (m, 1H, exo), 4.65 (q, *J* = 6.3 Hz, 1H), 3.69 (d, *J* = 2.9 Hz, 3H), 3.55 (d, *J* = 7.1 Hz, 1H, endo), 3.28 (dd, *J* = 5.7, 2.9 Hz, 2H), 2.99 – 2.88 (m, 2H, exo), 2.43 (dd, *J* = 9.9, 6.4 Hz, 2H, exo), 1.83 – 1.76 (m, 6H), 1.59 (s, 3H, exo), 1.43 (s, 9H). ^{13}C NMR (101 MHz, $CDCl_3$) δ 172.9, 155.4, 145.7, 135.2, 133.5, 127.7, 124.8, 122.5, 122.2, 121.9, 121.6, 120.0, 120.0, 116.8, 110.9, 110.7, 110.4, 79.9, 54.3, 52.4, 37.7, 30.8, 29.7, 28.5, 28.2, 25.9, 22.9, 18.1. HRMS $(M+H)^+$ calcd for $C_{22}H_{30}N_2O_4$ 387.2284, found 387.2278.

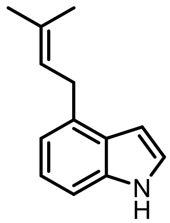

4-(3-Methylbut-2-en-1-yl)-1*H*-indole (14)[1]**:** The compound was prepared following the general procedure A and was obtained as a liquid 29% (brsm 79%) or following the general procedure F, using 4-allylindole (210 mg, 1.34 mmol) as the starting material gave 77% of the product. ^{1}H NMR (400 MHz, chloroform-*d*) δ 8.14 (s, 1H), 7.26 (d, *J* = 8.1 Hz, 1H), 7.20 (t, *J* = 3.0 Hz, 1H), 7.18 – 7.10 (m, 1H), 6.95 (d, *J* = 7.1 Hz, 1H), 6.65 – 6.56 (m, 1H), 5.46 (ddt, *J* = 7.3, 5.7, 1.5 Hz, 1H), 3.64 (d, *J* = 7.2 Hz, 2H), 1.81 (s, 3H), 1.76 (t, *J* = 1.4 Hz, 3H). ^{13}C NMR (101 MHz, $CDCl_3$) δ 135.9, 134.1, 132.2, 127.3, 123.6, 123.2, 122.3, 119.0, 109.0, 101.2, 32.3, 25.9, 18.0. HRMS $(M+H)^+$ calcd for $C_{13}H_{15}N$ 186.1277, found 186.1272.

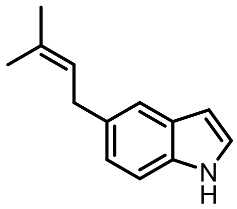

5-(3-Methylbut-2-en-1-yl)-1*H*-indole (15)[2]**:** The compound was prepared following the general procedure A and was obtained as a liquid 42% (brsm 72%) or following the general procedure F, using 5-allylindole (380 mg, 2.42 mmol) as the starting material gave 79% of the prenyl indole. ^{1}H NMR (400 MHz, chloroform-*d*) δ 8.03 (s, 1H), 7.45 (d, *J* = 1.7 Hz, 1H), 7.31 (d, *J* = 8.3 Hz, 1H), 7.17 (t, *J* = 2.8 Hz, 1H), 7.05 (dd, *J* = 8.3, 1.6 Hz, 1H), 6.53 – 6.47 (m, 1H), 5.42 (tq, *J* = 7.3, 1.5 Hz, 1H), 3.46 (d, *J* = 7.4 Hz, 2H), 1.77 (t, *J* = 1.7 Hz, 6H). ^{13}C NMR (101 MHz, $CDCl_3$) δ 134.5, 133.3, 131.7, 128.3, 124.6, 124.4, 123.1, 119.8, 110.9, 102.4, 34.5, 25.9, 17.9. HRMS $(M+H)^+$ calcd for $C_{13}H_{15}N$ 186.1277, found 186.1281.

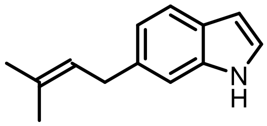

6-(3-Methylbut-2-en-1-yl)-1*H*-indole (16): The compound was prepared following the general procedure A and was obtained as a liquid 31% (brsm 81%) or following the general procedure F, using 6-allylindole (300 mg, 1.91 mmol) as the starting material gave 72% of product. ^{1}H NMR (400 MHz, chloroform-*d*) δ 8.03 (s, 1H), 7.55 (d, *J* = 8.0 Hz, 1H), 7.20 (s, 1H), 7.15 (t, *J* = 2.8 Hz, 1H), 6.97 (d, *J* = 8.1 Hz, 1H), 6.51 (s, 1H), 5.40 (t, *J* = 7.4 Hz, 1H), 3.46 (d, *J* = 7.2 Hz, 2H), 1.76 (s, 6H). ^{13}C NMR (101 MHz, $CDCl_3$) δ 136.4, 136.0, 132.1, 126.1, 124.1, 123.7, 121.1, 120.6, 110.3, 102.6, 34.7, 25.9, 18.0. HRMS $(M+H)^+$ calcd for $C_{13}H_{15}N$ 186.1277, found 186.1279.

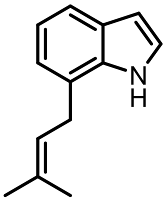

7-(3-Methylbut-2-en-1-yl)-1*H*-indole(17)[3]**:** The compound was prepared following the general procedure A and was obtained as a liquid 35% (brsm 79%) or following the general procedure F, using 7-allylindole (350 mg, 2.23 mmol) as the starting material gave 75% of expected product. ^{1}H NMR (400 MHz, chloroform-*d*) δ 8.16 (s, 1H), 7.52 (d, *J* = 7.7 Hz, 1H), 7.20 (t, *J* = 2.8 Hz, 1H), 7.06 (t, *J* = 7.5 Hz, 1H), 7.01 (d, *J* = 7.1 Hz, 1H), 6.56 (dd, *J* = 3.2, 2.1 Hz, 1H), 5.47 – 5.38 (m, 1H), 3.59 (d, *J* = 7.0 Hz, 2H), 1.83 (s, 3H), 1.79 (s, 3H). ^{13}C NMR (101 MHz, $CDCl_3$) δ 135.2, 133.5, 127.9, 124.0, 123.9, 122.4, 121.6, 120.1, 118.9, 103.1, 31.0, 25.9, 18.1. HRMS $(M+H)^+$ calcd for $C_{13}H_{15}N$ 186.1277, found 186.1281.

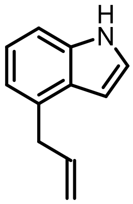

4-Allyl-1*H*-indole (18): The compound was prepared following the general procedure B and was obtained as a liquid 87% ^{1}H NMR (400 MHz, chloroform-*d*) δ 8.07 (s, 1H), 7.47 (dt, *J* = 1.6, 0.8 Hz, 1H), 7.33 (dt, *J* = 8.3, 0.9 Hz, 1H), 7.18 (dd, *J* = 3.2, 2.4 Hz, 1H), 7.05 (dd, *J* = 8.3, 1.7 Hz, 1H), 6.51 (ddd, *J* = 3.1, 2.0, 1.0 Hz, 1H), 6.05 (ddt, *J* = 16.8, 10.0, 6.7 Hz, 1H), 5.15 – 5.03 (m, 2H), 3.50 (d, *J* = 6.7 Hz, 2H). ^{13}C NMR (101 MHz, $CDCl_3$) δ 138.8, 134.6, 131.5, 128.3, 124.5, 123.2, 120.2, 115.1, 111.0, 102.5, 40.5. HRMS $(M+H)^+$ calcd for $C_{11}H_{11}N$ 158.0964, found 158.0962.

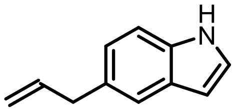

5-Allyl-1*H*-indole (19) [2]**:** The compound was prepared following the general procedure B and was obtained as a liquid 89%. ^{1}H NMR (500 MHz, chloroform-*d*) δ 8.11 (s, 1H), 7.28 (d, *J* = 8.1 Hz, 1H), 7.19 (h, *J* = 3.6, 3.2 Hz, 2H), 7.00 (dd, *J* = 7.3, 3.4 Hz, 1H), 6.64 (q, *J* = 2.7 Hz, 1H), 6.14 (ddtd, *J* = 16.6, 9.8, 6.7, 3.1 Hz, 1H), 5.26 – 5.09 (m, 2H), 3.76 – 3.65 (m, 2H). ^{13}C NMR (126 MHz, $CDCl_3$) δ 137.3, 135.9, 132.3, 127.3, 123.8, 123.8, 122.3, 119.4, 115.7, 115.6, 109.4, 109.3, 101.1, 38.0. HRMS $(M+H)^+$ calcd for $C_{11}H_{11}N$ 158.0964, found 158.0961.

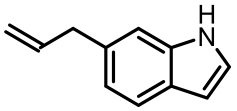

6-Allyl-1*H*-indole (20): The compound was prepared following the general procedure B and was obtained as a liquid 92%.^{1}H NMR (400 MHz, chloroform-*d*) δ 8.10 (s, 1H), 7.64 (d, *J* = 8.1 Hz, 1H), 7.20 (dd, *J* = 3.2, 2.4 Hz, 1H), 7.05 (dd, *J* = 8.1, 1.5 Hz, 1H), 6.61 – 6.54 (m, 1H), 6.11 (ddt, *J* = 16.8, 10.0, 6.7 Hz, 1H), 5.23 – 5.10 (m, 2H), 3.62 – 3.51 (m, 2H). ^{13}C NMR (101 MHz, $CDCl_3$) δ 138.4, 136.3, 134.2, 126.3, 123.9, 121.2, 120.6, 115.4, 110.7, 102.5, 40.6. HRMS $(M+H)^+$ calcd for $C_{11}H_{11}N$ 158.0964, found 158.0962.

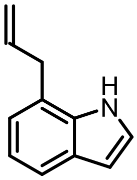

7-Allyl-1*H*-indole (21)[4]**:** The compound was prepared following the general procedure B and was obtained as a colorless liquid in 92%.^{1}H NMR (400 MHz, chloroform-*d*) δ 8.25 (s, 1H), 7.55 (d, *J* = 7.8 Hz, 1H), 7.20 (dd, *J* = 3.2, 2.4 Hz, 1H), 7.11 – 7.04 (m, 1H), 7.02 (dd, *J* = 7.1, 1.1 Hz, 1H), 6.56 (dd, *J* = 3.2, 2.0 Hz, 1H), 6.09 (ddt, *J* = 16.6, 10.0, 6.5 Hz, 1H), 5.31 – 5.14 (m, 2H), 3.66 (dt, *J* = 6.7, 1.8 Hz, 2H). ^{13}C NMR (101 MHz, $CDCl_3$) δ 136.9, 135.4, 128.1, 124.0, 122.2, 122.1, 120.1, 119.3, 116.5, 103.0, 37.0. HRMS $(M+H)^+$ calcd for $C_{11}H_{11}N$ 158.0964, found 158.0961.

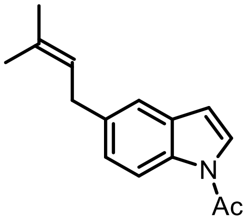

1-(5-(3-Methylbut-2-en-1-yl)-1*H*-indol-1-yl)ethan-1-one (22): The compound was prepared following the acetylation part in the general procedure C and was obtained as a liquid 79%. ^{1}H NMR (400 MHz, chloroform-*d*) δ 8.32 (d, *J* = 8.5 Hz, 1H), 7.40 – 7.37 (m, 1H), 7.36 (dd, *J* = 1.8, 0.9 Hz, 1H), 7.18 (dd, *J* =

8.5, 1.8 Hz, 1H), 6.58 (dd, J = 3.7, 0.8 Hz, 1H), 5.37 (tdt, J = 7.3, 2.9, 1.4 Hz, 1H), 3.44 (d, J = 7.4 Hz, 2H), 2.62 (s, 3H), 1.76 (s, 6H). ^{13}C NMR (101 MHz, $CDCl_3$) δ 168.6, 137.4, 134.1, 132.5, 130.8, 125.9, 125.4, 123.7, 120.1, 116.4, 109.2, 34.4, 25.9, 24.0, 18.0. HRMS $(M+H)^+$ calcd for $C_{15}H_{17}NO$ 228.1382, found 228.1377.

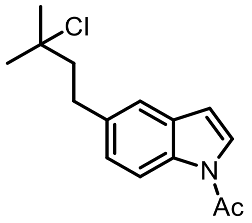

1-(5-(3-Chloro-3-methylbutyl)-1*H*-indol-1-yl)ethan-1-one (23): To a dry round-bottomed flask, 1-(5-(3-methylbut-2-en-1-yl)-1*H*-indol-1-yl)ethan-1-one (38 mg, 0.167 mmol) and dry dioxane (3 mL) were added under an argon atmosphere. The reaction mixture was cooled to 0 °C, and 4 M HCl (10 equiv) was added dropwise. The mixture was stirred at the same temperature until the starting material was completely consumed (≈3 h). After completion, the crude mixture was purified by silica gel chromatography (hexanes/EtOAc) to afford 1-(5-(3-chloro-3-methylbutyl)-1*H*-indol-1-yl)ethan-1-one as a sticky gel in 62% yield.^{1}H NMR (500 MHz, chloroform-*d*) δ 8.34 (d, J = 8.4 Hz, 1H), 7.43 – 7.36 (m, 2H), 7.20 (dd, J = 8.5, 1.8 Hz, 1H), 6.59 (dd, J = 3.8, 0.8 Hz, 1H), 2.95 – 2.87 (m, 2H), 2.63 (s, 3H), 2.13 – 2.05 (m, 2H), 1.66 (s, 6H). ^{13}C NMR (126 MHz, $CDCl_3$) δ 168.6, 137.4, 134.2, 130.9, 125.9, 125.6, 120.4, 116.6, 109.1, 70.8, 48.6, 32.6, 31.7, 24.0. HRMS $(M+H)^+$ calcd for $C_{15}H_{18}ClNO$ 264.1149, found 264.1148.

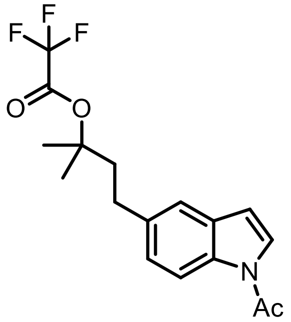

4-(1-Acetyl-1*H*-indol-5-yl)-2-methylbutan-2-yl 2,2,2-trifluoroacetate (24): To a dry round-bottom flask, 1-(5-(3-methylbut-2-en-1-yl)-1*H*-indol-1-yl)ethan-1-one (36 mg, 0.158 mmol) and dry CH_2Cl_2 (2.0 mL) were added under an argon atmosphere. The reaction mixture was cooled to 0 °C, and TFA (10 equiv) was added dropwise. The mixture was stirred at the same temperature until complete consumption of the starting material (≈5 h). After completion, the crude mixture was purified by silica gel chromatography (hexanes/EtOAc) to afford 1-(5-(3-chloro-3-methylbutyl)-1*H*-indol-1-yl)ethan-1-one as a white solid in 72% yield. ^{1}H NMR (400 MHz, acetone-d_6) δ 8.32 (d, J = 8.4 Hz, 1H), 7.71 (d, J = 3.7 Hz, 1H), 7.46 – 7.41 (m, 1H), 7.20 (dd, J = 8.5, 1.6 Hz, 1H), 6.64 (d, J = 3.7 Hz, 1H), 2.84 – 2.77 (m, 2H), 2.65 (s, 3H), 2.27 – 2.20 (m, 2H), 1.67 (s, 6H). ^{13}C NMR (101 MHz, acetone) δ 206.20, 169.65, 157.24, 156.84, 156.44, 156.03,

137.47, 135.01, 131.92, 127.53, 125.97, 120.95, 117.01, 108.93, 90.35, 43.45, 30.62, 25.71, 23.84. ^{19}F NMR (377 MHz, acetone) δ -76.52. HRMS $(M+NH_4)^+$ calcd for $C_{17}H_{18}F_3NO_3$ 359.1577, found 359.1575.

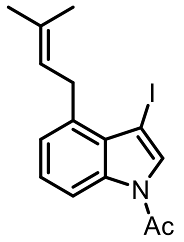

1-(3-Iodo-4-(3-methylbut-2-en-1-yl)-1*H*-indol-1-yl) ethan-1-one (25): The compound was prepared following the general procedure C and was obtained as a brownish liquid in 76% yield. ^{1}H NMR (400 MHz, Chloroform-*d*) δ 8.42 (d, *J* = 8.3 Hz, 1H), 7.61 (s, 1H), 7.32 (t, *J* = 7.9 Hz, 1H), 7.13 (d, *J* = 7.5 Hz, 1H), 5.41 (td, *J* = 6.3, 3.1 Hz, 1H), 4.03 (d, *J* = 7.0 Hz, 2H), 2.64 (s, 3H), 1.83 – 1.75 (m, 6H). ^{13}C NMR (101 MHz, $CDCl_3$) δ 167.6, 135.7, 135.1, 132.8, 131.3, 127.0, 126.2, 125.0, 123.2, 114.6, 62.5, 29.9, 25.9, 24.2, 18.3. HRMS $(M)^+$ calcd for $C_{15}H_{15}INO$ 352.0193, found 352.0181.

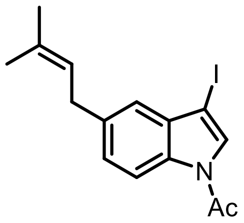

1-(3-Iodo-5-(3-methylbut-2-en-1-yl)-1*H*-indol-1-yl) ethan-1-one (26): The compound was prepared following the general procedure C and obtained as a brownish jelly in 68% yield. ^{1}H NMR (400 MHz, Chloroform-*d*) δ 8.27 (d, *J* = 8.5 Hz, 1H), 7.53 (s, 1H), 7.22 (dd, *J* = 8.4, 1.8 Hz, 1H), 7.17 (d, *J* = 1.8 Hz, 1H), 5.45 – 5.28 (m, 1H), 3.47 (d, *J* = 7.3 Hz, 2H), 2.60 (s, 3H), 1.77 (d, *J* = 3.0 Hz, 6H). ^{13}C NMR (101 MHz, $CDCl_3$) δ 167.6, 138.3, 133.5, 132.8, 132.3, 129.6, 129.6, 127.0, 123.4, 120.7, 116.3, 68.1, 34.4, 25.9, 23.9, 18.0. HRMS $(M+H)^+$ calcd for $C_{15}H_{16}INO$ 354.0349, found 354.0354.

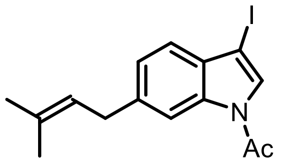

1-(3-Iodo-6-(3-methylbut-2-en-1-yl)-1*H*-indol-1-yl) ethan-1-one (27): The compound was prepared following the general procedure C and was obtained as a brownish liquid in 79% yield. ^{1}H NMR (400 MHz, chloroform-*d*) δ 8.24 (s, 1H), 7.51 (s, 1H), 7.30 (d, *J* = 8.0 Hz, 1H), 7.20 (dd, *J* = 8.1, 1.5 Hz, 1H), 5.36 (tdt, *J* = 7.4, 2.9, 1.5 Hz, 1H), 3.48 (d, *J* = 7.4 Hz, 2H), 2.62 (s, 3H), 1.75 (s, 6H). ^{13}C NMR (101 MHz, $CDCl_3$) δ 167.8, 140.9, 135.5, 132.8, 130.3, 129.1, 125.2, 123.5, 121.3, 116.1, 68.0, 34.9, 25.9, 24.1, 18.0. HRMS $(M+H)^+$ calcd for $C_{15}H_{16}INO$ 354.0349, found 354.0352.

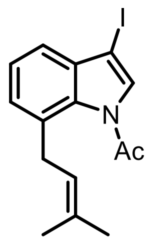

1-(3-Iodo-7-(3-methylbut-2-en-1-yl)-1*H*-indol-1-yl) ethan-1-one (28): The compound was prepared following the general procedure C and was obtained as a brownish liquid in 74% yield. ^{1}H NMR (400 MHz, chloroform-*d*) δ 7.53 (s, 1H), 7.34 – 7.26 (m, 3H), 5.12 (dddd, *J* = 6.9, 5.5, 2.8, 1.4 Hz, 1H), 3.67 (d, *J* = 6.9 Hz, 2H), 2.63 (s, 3H), 1.70 (s, 6H). ^{13}C NMR (101 MHz, $CDCl_3$) δ 166.9, 134.1, 133.8, 133.2, 130.8, 130.6, 128.1, 124.9, 122.7, 119.6, 67.7, 33.9, 25.9, 24.3, 18.1 HRMS $(M+H)^+$ calcd for $C_{15}H_{16}INO$ 354.0349, found 354.0354.

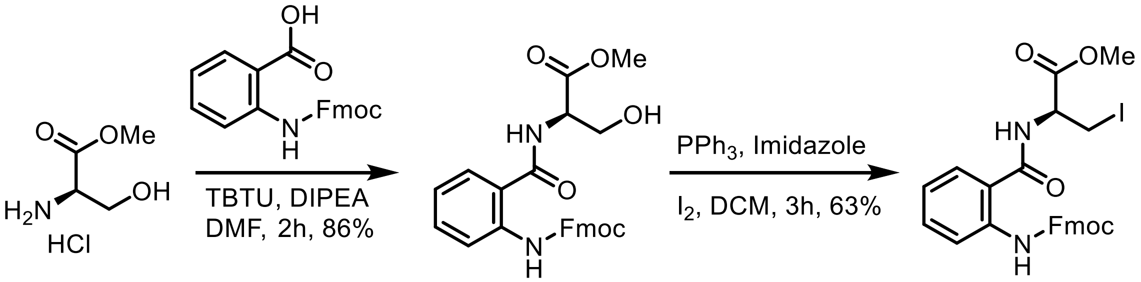

Methyl (*S*)-2-(2-((((9*H*-fluoren-9-yl)methoxy)carbonyl)amino)benzamido)-3-iodopropanoate (29):
Step 1:

D-Serine methyl ester hydrochloride (1.2 equiv), TBTU (1.2 equiv), 2-((((9*H*-fluoren-9-yl)methoxy)carbonyl)amino)benzoic acid (1.47 g, 4.09 mmol), and DIPEA (2.0 equiv) were dissolved in DMF (15 mL) and stirred for 3.5 h. TLC indicated complete consumption of the starting material. The solvent was removed under reduced pressure, and the residue was dissolved in water (10 mL) and extracted with EtOAc (3 × 10 mL). The combined organic layers were dried over Na_2SO_4, filtered, and purified by column chromatography (EtOAc/hexanes) to afford methyl (2-((((9*H*-fluoren-9-yl)methoxy)carbonyl)amino)benzoyl)-D-serinate as a colourless liquid (1.62 g, 86% yield). ^{1}H NMR (400 MHz, chloroform-*d*) δ 10.53 (s, 1H), 8.34 (d, *J* = 8.5 Hz, 1H), 7.80 (d, *J* = 7.5 Hz, 2H), 7.68 (d, *J* = 7.5 Hz, 2H), 7.62 (dd, *J* = 7.9, 1.5 Hz, 1H), 7.54 – 7.46 (m, 1H), 7.43 (t, *J* = 7.4 Hz, 2H), 7.39 – 7.31 (m, 2H), 7.28 (d, *J* = 2.3 Hz, 1H), 7.07 (td, *J* = 7.6, 1.2 Hz, 1H), 4.89 (dt, *J* = 7.2, 3.4 Hz, 1H), 4.51 – 4.41 (m, 2H), 4.33 (t, *J* = 7.4 Hz, 1H), 4.20 – 4.02 (m, 2H), 3.84 (s, 3H), 2.78 (s, 1H). ^{13}C NMR (101 MHz, $CDCl_3$) δ 170.9, 169.1, 153.8, 143.9, 141.4, 139.9, 133.2, 127.9, 127.2, 127.2, 125.4, 122.3, 120.3, 120.1, 119.4, 67.4, 63.1, 55.0, 53.0, 47.1. HRMS $(M+H)^+$ calcd for $C_{26}H_{24}N_2O_6$ 461.1707, found 461.1711.

Step 2: To a solution of triphenylphosphine (1.3 equiv) and imidazole (1.3 equiv) in dry CH_2Cl_2 (20 mL) at 0 °C, iodine (1.3 equiv.) was added in three portions over 30 min. A solution of methyl (2-((((9*H*-fluoren-9-

yl)methoxy)carbonyl)amino)benzoyl)-D-serinate (1.00 g, 2.2 mmol) in dry CH_2Cl_2 (20 mL) was then added dropwise. The resulting solution was stirred at 0 °C for 1 h and then warmed to rt for 1 h. Methanol (10 mL) was added, and the solvent was removed *i*n vacuo. The crude product was purified by flash column chromatography to afford the iodide as a white foam (0.8 g, 63%). The purification should be performed quickly, as the iodide is prone to β-elimination. ^{1}H NMR (500 MHz, chloroform-*d*) δ 10.48 (s, 1H), 8.38 (d, *J* = 8.5 Hz, 1H), 7.78 (dt, *J* = 7.6, 0.9 Hz, 2H), 7.70 – 7.61 (m, 3H), 7.53 (ddd, *J* = 8.6, 7.3, 1.5 Hz, 1H), 7.45 – 7.37 (m, 2H), 7.33 (tt, *J* = 7.5, 1.1 Hz, 2H), 7.12 (ddd, *J* = 8.2, 7.5, 1.2 Hz, 1H), 7.02 (d, *J* = 6.9 Hz, 1H), 5.00 (dt, *J* = 7.2, 3.7 Hz, 1H), 4.48 – 4.38 (m, 2H), 4.31 (t, *J* = 7.5 Hz, 1H), 3.88 (s, 3H), 3.79 (dd, *J* = 10.5, 3.9 Hz, 1H), 3.73 (dd, *J* = 10.5, 3.5 Hz, 1H). ^{13}C NMR (126 MHz, $CDCl_3$) δ 169.9, 168.5, 153.7, 144.0, 144.0, 141.4, 140.2, 133.5, 127.9, 127.3, 125.4, 122.4, 120.3, 120.1, 118.9, 67.5, 53.6, 52.8, 47.2, 7.1. HRMS $(M+Na)^+$ calcd. for $C_{26}H_{23}IN_2O_5$ 593.0543, found 593.0530.

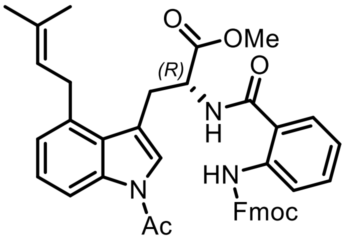

Methyl (*R*)-2-(2-((((9*H*-fluoren-9-yl)methoxy)carbonyl)amino)benzamido)-3-(1-acetyl-4-(3-methylbut-2-en-1-yl)-1*H*-indol-3-yl)propanoate (30): The compound was prepared following the general procedure D and was obtained as a brownish liquid in 42% yield. $[\alpha]_D^{25}$ -54.5 (*c* 1.2, $CHCl_3$). ^{1}H NMR (400 MHz, chloroform-*d*) δ 10.48 (s, 1H), 8.35 (t, *J* = 9.1 Hz, 2H), 7.78 (d, *J* = 7.5 Hz, 2H), 7.69 – 7.62 (m, 2H), 7.49 – 7.44 (m, 1H), 7.44 – 7.36 (m, 5H), 7.35 – 7.28 (m, 3H), 7.25 (s, 2H), 7.11 – 7.06 (m, 1H), 7.00 (td, *J* = 7.6, 1.1 Hz, 1H), 6.77 (d, *J* = 8.3 Hz, 1H), 5.28 – 5.19 (m, 2H), 4.48 – 4.38 (m, 2H), 4.31 (t, *J* = 7.4 Hz, 1H), 3.81 (s, 3H), 3.71 (t, *J* = 7.6 Hz, 2H), 3.61 (ddd, *J* = 15.6, 5.1, 1.2 Hz, 1H), 3.46 – 3.34 (m, 1H), 2.53 (s, 3H), 1.74 (s, 6H). ^{13}C NMR (101 MHz, $CDCl_3$) δ 172.3, 168.8, 168.4, 153.6, 144.0, 143.9, 141.4, 141.4, 140.1, 136.7, 134.7, 133.3, 133.2, 128.0, 127.9, 127.3, 126.8, 125.8, 125.4, 124.9, 123.6, 123.3, 122.2, 120.3, 120.1, 119.2, 117.6, 114.9, 67.5, 52.9, 52.7, 47.1, 32.1, 30.3, 25.8, 24.2, 18.2. HRMS $(M+H)^+$ calcd for $C_{41}H_{39}N_3O_6$ 670.2912, found 670.2902.

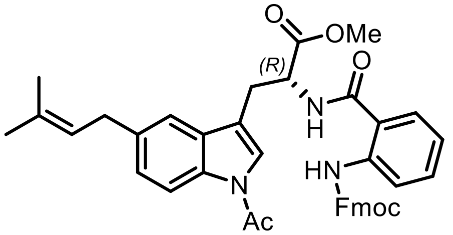

Methyl (*R*)-2-(2-((((9*H*-fluoren-9-yl)methoxy)carbonyl)amino)benzamido)-3-(1-acetyl-5-(3-methylbut-2-en-1-yl)-1*H*-indol-3-yl) propanoate (31): The compound was prepared following the general procedure D and was obtained as a brownish liquid in 54% yield. $[\alpha]_D^{25}$ -45.6 (*c* 2.5, $CHCl_3$). ^{1}H NMR (500 MHz,

chloroform-*d*) δ 10.66 (s, 1H), 8.47 – 8.23 (m, 2H), 7.80 (dd, *J* = 7.5, 1.1 Hz, 2H), 7.70 (ddq, *J* = 7.4, 5.5, 1.0 Hz, 2H), 7.50 (ddd, *J* = 8.7, 7.3, 1.5 Hz, 1H), 7.43 (tt, *J* = 7.5, 0.9 Hz, 3H), 7.35 (tt, *J* = 7.5, 1.4 Hz, 3H), 7.31 (dd, *J* = 7.9, 1.5 Hz, 2H), 7.28 – 7.25 (m, 2H), 7.20 (dd, *J* = 8.5, 1.7 Hz, 1H), 7.00 (ddd, *J* = 8.1, 7.4, 1.2 Hz, 1H), 6.79 (d, *J* = 7.4 Hz, 1H), 5.26 (tp, *J* = 7.3, 1.4 Hz, 1H), 5.20 (dt, *J* = 7.4, 5.3 Hz, 1H), 4.51 – 4.42 (m, 2H), 4.35 (t, *J* = 7.5 Hz, 1H), 3.80 (s, 3H), 3.49 (ddd, *J* = 14.9, 5.5, 0.9 Hz, 1H), 3.44 – 3.32 (m, 3H), 2.57 (d, *J* = 3.1 Hz, 3H), 1.77 – 1.68 (m, 6H).^{13}C NMR (126 MHz, $CDCl_3$) δ 171.8, 168.6, 168.2, 153.7, 144.0, 144.0, 141.4, 140.2, 137.7, 133.3, 132.7, 127.9, 127.3, 126.9, 126.4, 125.4, 125.4, 123.8, 123.4, 122.2, 120.3, 120.1, 119.1, 118.0, 116.7, 67.5, 53.1, 52.9, 47.2, 34.4, 27.53, 25.9, 24.0, 17.9. HRMS $(M+H)^+$ calcd for $C_{41}H_{39}N_3O_6$ 670.2911, found 670.2907

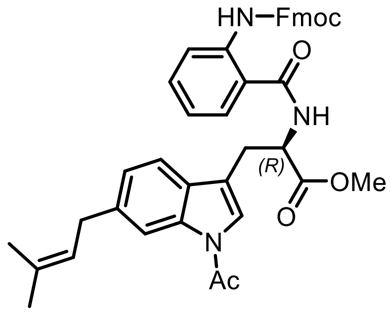

Methyl (*R*)-2-(2-((((9*H*-fluoren-9-yl)methoxy)carbonyl)amino)benzamido)-3-(1-acetyl-6-(3-methylbut-2-en-1-yl)-1*H*-indol-3-yl) propanoate (32): The compound was prepared following the general procedure D and was obtained as a light yellow gelly in 52% yield. $[\alpha]_D^{25}$ -45.0 (*c* 2.5, $CHCl_3$). ^{1}H NMR (400 MHz, chloroform-*d*) δ 10.63 (s, 1H), 8.38 (d, *J* = 8.2 Hz, 1H), 8.29 (s, 1H), 7.80 (d, *J* = 7.5 Hz, 2H), 7.74 – 7.67 (m, 2H), 7.49 (t, *J* = 7.9 Hz, 1H), 7.46 – 7.40 (m, 4H), 7.35 (t, *J* = 7.4 Hz, 3H), 7.24 (s, 1H), 7.13 (dd, *J* = 8.1, 1.4 Hz, 1H), 7.03 – 6.96 (m, 1H), 6.89 (d, *J* = 7.4 Hz, 1H), 5.44 – 5.31 (m, 1H), 5.24 – 5.14 (m, 1H), 4.49 – 4.43 (m, 2H), 4.35 (t, *J* = 7.5 Hz, 1H), 3.80 (s, 3H), 3.47 (m, 3H), 3.37 (dd, *J* = 14.8, 5.5 Hz, 1H), 2.55 (s, 3H), 1.83 – 1.70 (m, 6H). ^{13}C NMR (101 MHz, $CDCl_3$) δ 171.9, 168.6, 168.5, 153.6, 143.9, 141.4, 140.1, 140.0, 136.3, 133.2, 132.6, 128.5, 127.9, 127.2, 126.9, 125.4, 124.5, 123.6, 123.1, 122.1, 120.2, 120.1, 119.2, 118.5, 116.8, 67.4, 52.9, 52.9, 47.1, 34.9, 27.6, 25.9, 24.1, 18.0. HRMS $(M+H)^+$ calcd for $C_{41}H_{39}N_3O_6$ 670.2911, found 670.2907.

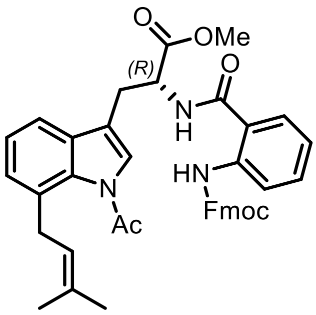

Methyl(*R*)-2-(2-((((9*H*-fluoren-9-yl)methoxy)carbonyl)amino)benzamido)-3-(1-acetyl-7-(3-methylbut-2-en-1-yl)-1*H*-indol-3-yl) propanoate (33): The compound was prepared following the general procedure

D and was obtained as a brown solid in 55% yield. $[\alpha]_D^{25}$ -59.3 (*c* 2.8, $CHCl_3$). ^{1}H NMR (400 MHz, chloroform-*d*) δ 10.66 (s, 1H), 8.38 (d, *J* = 8.1 Hz, 1H), 7.81 (d, *J* = 7.5 Hz, 2H), 7.71 (dd, *J* = 7.4, 2.1 Hz, 2H), 7.46 (dt, *J* = 19.1, 7.4 Hz, 3H), 7.41 – 7.32 (m, 4H), 7.28 – 7.21 (m, 3H), 7.00 (t, *J* = 7.2 Hz, 1H), 6.86 (d, *J* = 7.4 Hz, 1H), 5.24 – 5.15 (m, 2H), 4.48 (dd, *J* = 7.4, 2.5 Hz, 2H), 4.35 (t, *J* = 7.4 Hz, 1H), 3.80 (s, 3H), 3.71 (d, *J* = 6.9 Hz, 2H), 3.54 – 3.35 (m, 2H), 2.58 (s, 3H), 1.75 (s, 6H). ^{13}C NMR (101 MHz, $CDCl_3$) δ 171.8, 168.5, 167.6, 153.6, 143.9, 141.3, 140.0, 134.7, 133.2, 132.9, 132.2, 130.8, 127.8, 127.4, 127.2, 126.9, 125.3, 124.9, 124.2, 122.8, 122.1, 120.2, 120.0, 119.1, 116.4, 116.2, 67.4, 52.8, 52.8, 47.0, 33.9, 27.4, 25.8, 24.3, 18.0. HRMS $(M+H)^+$ calcd for $C_{41}H_{39}N_3O_6$ 670.2911, found 670.2907.

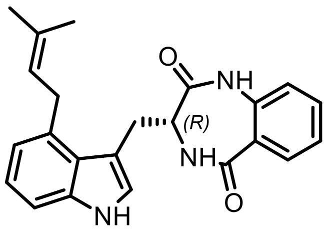

(*R*)-3-((4-(3-Methylbut-2-en-1-yl)-1*H*-indol-3-yl)methyl)-3,4-dihydro-1*H*-benzo[e][1,4]diazepine-2,5-dione (1)[5]**:** The compound was synthesized following the general procedure E and was obtained as a white solid in 59% yield. $[\alpha]_D^{25}$ -51.37 (c 5.4, MeOH) [lit. $[\alpha]_D^{25}$ -50.9 (*c* 0.1, MeOH)[5]]; ^{1}H NMR (400 MHz, acetone-d_6) δ 10.11 (s, 1H), 9.47 (s, 1H), 7.82 (dd, *J* = 8.1, 1.7 Hz, 1H), 7.58 – 7.40 (m, 2H), 7.32 (d, *J* = 2.5 Hz, 1H), 7.28 – 7.16 (m, 3H), 6.98 (dd, *J* = 8.1, 7.1 Hz, 1H), 6.76 (dd, *J* = 7.2, 0.9 Hz, 1H), 5.23 (dddd, *J* = 8.1, 6.6, 2.9, 1.4 Hz, 1H), 4.16 (dt, *J* = 8.7, 5.5 Hz, 1H), 3.68 (dd, *J* = 15.4, 5.4 Hz, 3H), 3.32 (dd, *J* = 15.5, 8.8 Hz, 1H), 1.70 (s, 3H), 1.65 (s, 3H). ^{13}C NMR (101 MHz, acetone) δ 172.3, 168.7, 138.6, 137.9, 134.7, 133.3, 132.6, 131.9, 127.5, 126.1, 125.4, 125.1, 125.0, 122.4, 122.0, 120.5, 111.6, 110.6, 54.5, 32.9, 26.9, 25.9, 18.2. HRMS $(M+H)^+$ calcd. for $C_{23}H_{23}N_3O_2$ 374.1863, found 374.1859.

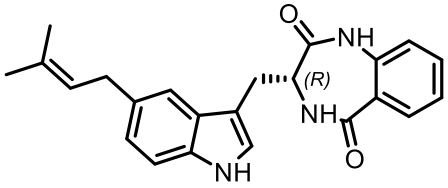

(*R*)-3-((5-(3-Methylbut-2-en-1-yl)-1*H*-indol-3-yl) methyl)-3,4-dihydro-1*H*-benzo[e][1,4]diazepine-2,5-dione (2)[5]**:** The compound was synthesized following the general procedure E and was obtained as a white solid in 54% yield. $[\alpha]_D^{25}$ -46.6 (c 7.8, MeOH) [lit. $[\alpha]_D^{25}$ -45.9 (*c* 0.1, MeOH)[5]]; ^{1}H NMR (400 MHz, acetone-d_6) δ 9.96 (s, 1H), 9.47 (s, 1H), 7.78 (d, *J* = 7.9 Hz, 1H), 7.54 – 7.49 (m, 1H), 7.41 (s, 1H), 7.36 (s, 1H), 7.30 (s, 1H), 7.27 (d, *J* = 8.3 Hz, 1H), 7.21 (dd, *J* = 7.6, 5.5 Hz, 3H), 6.91 (d, *J* = 8.3 Hz, 1H), 5.30 (t, *J* = 7.3 Hz, 1H), 4.23 – 4.15 (m, 1H), 3.44 (dd, *J* = 15.2, 5.3 Hz, 1H), 3.34 (d, *J* = 7.2 Hz, 2H), 3.19 (dd, *J* = 15.1, 9.2 Hz, 1H), 1.67 (s, 6H). ^{13}C NMR (101 MHz, acetone) δ 172.4, 168.6, 137.8, 136.2, 133.2, 132.9, 131.7, 131.5, 128.6, 127.3, 125.7, 125.1, 124.9, 123.2, 121.8, 118.1, 112.1, 110.6, 53.6, 35.1, 25.9, 24.9, 17.8. HRMS $(M+H)^+$ calcd. for $C_{23}H_{23}N_3O_2$ 374.1863, found 374.1859

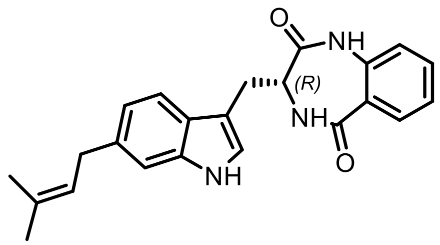

***(R)*-3-((6-(3-Methylbut-2-en-1-yl)-1*H*-indol-3-yl) methyl)-3,4-dihydro-1*H*-benzo[e][1,4]diazepine-2,5-dione (3)[5]:** The compound was synthesized following the general procedure E and was obtained as a light-yellow solid in 55% yield. $[\alpha]_D^{25}$ -43.06 (*c* 9.8, MeOH) [lit. $[\alpha]_D^{25}$ -42.9 (*c* 0.1, MeOH)[5]]; ^{1}H NMR (400 MHz, acetone-d_6) δ 9.93 (s, 1H), 9.46 (s, 1H), 7.81 – 7.71 (m, 1H), 7.55 – 7.43 (m, 3H), 7.27 (d, *J* = 2.4 Hz, 1H), 7.24 – 7.12 (m, 4H), 6.81 (dd, *J* = 8.2, 1.5 Hz, 1H), 5.32 (m, 1H), 4.23 – 4.11 (m, 1H), 3.48 – 3.33 (m, 3H), 3.18 (dd, *J* = 15.1, 9.2 Hz, 1H), 1.70 (dd, *J* = 3.8, 1.4 Hz, 6H). ^{13}C NMR (101 MHz, acetone) δ 172.4, 168.6, 138.1, 137.8, 134.0, 133.2, 131.8, 131.7, 127.3, 126.5, 125.3, 124.9, 124.5, 121.8, 120.8, 118.9, 111.5, 110.8, 53.6, 35.1, 25.8, 25.0, 17.8. HRMS $(M+H)^+$ calcd. for $C_{23}H_{23}N_3O_2$ 374.1863, found 374.1859.

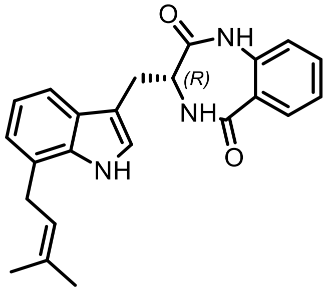

(*R*)-3-((7-(3-Methylbut-2-en-1-yl)-1*H*-indol-3-yl)methyl)-3,4-dihydro-1*H*-benzo[e][1,4]diazepine-2,5-dione (4)[5]: The compound was synthesized following the general procedure E and was obtained as a light brown solid in 65% yield. $[\alpha]_D^{25}$ -47.3 (*c* 3.55, MeOH) [lit. $[\alpha]_D^{25}$ -53.2 (*c* 0.1, MeOH)[5]]; ^{1}H NMR (400 MHz, acetone-d_6) δ 9.92 (s, 1H), 9.52 (s, 1H), 7.78 (dd, *J* = 8.0, 1.6 Hz, 1H), 7.55 – 7.49 (m, 2H), 7.44 (dt, *J* = 5.1, 3.5 Hz, 1H), 7.35 (d, *J* = 2.5 Hz, 1H), 7.21 (td, *J* = 7.8, 1.0 Hz, 2H), 6.94 – 6.90 (m, 2H), 5.42 (tdt, *J* = 7.2, 2.9, 1.5 Hz, 1H), 4.21 (dt, *J* = 9.2, 5.4 Hz, 1H), 3.57 (dd, *J* = 7.3, 1.5 Hz, 2H), 3.52 – 3.44 (m, 1H), 3.22 (dd, *J* = 15.1, 9.3 Hz, 1H), 1.74 (d, *J* = 6.5 Hz, 6H). ^{13}C NMR (101 MHz, acetone) δ 172.4, 168.7, 137.8, 136.4, 133.2, 131.7, 128.3, 127.3, 125.3, 124.9, 124.9, 123.0, 121.8, 121.6, 120.0, 116.9, 111.4, 53.6, 30.32, 25.8, 25.0, 17.9. HRMS $(M+H)^+$ calcd. for $C_{23}H_{23}N_3O_2$ 374.1863, found 374.1859.

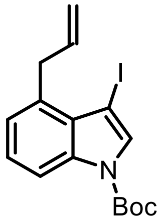

***tert*-Butyl 4-allyl-3-iodo-1*H*-indole-1-carboxylate (34):** To a solution of 4-allylindole (222 mg, 1.41 mmol) in DMF (0.8 mL) was added KOH pellets (2.5 equiv). A solution of iodine (1.0 equiv) in DMF (3 mL) was added dropwise over 30 min under stirring. The reaction mixture was stirred at rt for 1 h, then poured into ice–water containing 0.5% NH_3 and 0.1% $Na_2S_2O_5$. The mixture was extracted with a 1:1 mixture of EtOAc/hexanes until no product remained in the aqueous phase. The combined organic layers were washed with cold water, dried over anhydrous Na_2SO_4, and concentrated in vacuo. The crude product was dissolved in CH_2Cl_2 (10 mL), and Boc_2O (1.2 equiv), K_2CO_3 (1.2 equiv), and DMAP (10 mol %) were added. The mixture was stirred at rt for 18 h and then filtered. The crude product was purified by column chromatography (hexanes) to afford the title compound as a colorless oil (0.389 g, 72% yield). ^{1}H NMR (400 MHz, chloroform-*d*) δ 7.67 (s, 1H), 7.36 – 7.20 (m, 3H), 5.99 – 5.85 (m, 1H), 5.05 – 4.91 (m, 2H), 3.91 (dt, *J* = 6.3, 1.7 Hz, 2H), 1.64 (s, 9H). ^{13}C NMR (101 MHz, $CDCl_3$) δ 148.6, 138.1, 135.5, 133.3, 132.0, 127.1, 125.2, 124.9, 116.0, 113.6, 84.4, 60.1, 35.1, 28.3. HRMS $(M+H)^+$ calcd for $C_{16}H_{18}INO_2$ 384.0455, found 384.0457.

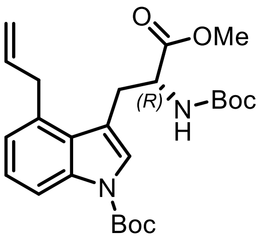

***tert*-Butyl (*R*)-4-allyl-3-(2-((*tert*-butoxycarbonyl)amino)-3-methoxy-3-oxopropyl)-1*H*-indole-1-carboxylate (36):** An oven-dried two-neck round-bottom flask was flame-dried twice under vacuum. After cooling to room temperature, Zn dust (70 mg, 1.02 mmol) was added under an argon atmosphere. The flask was evacuated and backfilled with argon three times. Dry DMF (6 mL) was added via syringe, followed by 1,2-dibromoethane (0.6 equiv.) to the stirred suspension. The mixture was stirred at 60 °C in an oil bath for 45 min, then cooled to rt. Chlorotrimethylsilane (0.2 equiv.) was added via syringe (gas evolution observed), and the mixture was stirred for 40 min at rt. A solution of methyl (*S*)-2-((tert-butoxycarbonyl)amino)-3-iodopropanoate[6] (1.50 equiv.) in dry DMF (3 mL) was added to the activated zinc suspension at rt, and the mixture was stirred at 35 °C for 1 h. The reaction mixture was cooled to rt, and under an argon atmosphere, $Pd_2(dba)_3$ (2.5 mol %) and 2-dicyclohexylphosphino-2′,6′-dimethoxybiphenyl (5 mol %) were added. A solution of *tert*-butyl 7-allyl-3-iodo-1*H*-indole-1-carboxylate (50 mg, 0.13 mmol, 1.0 equiv) in DMF (1 mL) was added immediately, and the mixture was stirred at 35 °C for 12 h. The reaction

mixture was filtered through a pad of Celite, diluted with EtOAc, washed with H_2O and brine, dried over anhydrous Na_2SO_4, filtered, and concentrated under reduced pressure. The product (39 mg, 65% yield) was purified by silica gel chromatography as a colorless liquid (5–8% alkene isomerization observed). ^{1}H NMR (500 MHz, chloroform-*d*) δ 8.09 (d, *J* = 8.5 Hz, 1H), 7.41 (s, 1H), 7.23 (d, *J* = 7.4 Hz, 1H), 7.02 (d, *J* = 7.4 Hz, 1H), 6.07 (ddt, *J* = 17.2, 10.2, 5.7 Hz, 1H), 5.16 – 4.81 (m, 3H), 4.64 (q, *J* = 8.2, 7.5 Hz, 1H), 3.83 – 3.66 (m, 5H), 3.38 (ddd, *J* = 15.3, 4.9, 1.1 Hz, 1H), 3.12 (dd, *J* = 15.3, 8.3 Hz, 1H), 1.65 (s, 9H), 1.41 (s, 9H). ^{13}C NMR (126 MHz, $CDCl_3$) δ 172.9, 155.3, 149.5, 137.7, 132.4, 129.2, 128.4, 124.7, 124.6, 124.5, 116.2, 115.7, 113.7, 83.7, 80.1, 54.1, 52.4, 37.2, 30.2, 28.3, 18.8. HRMS $(M+H)^+$ calcd for $C_{25}H_{34}N_2O_6$ 459.2489, found 459.2485.

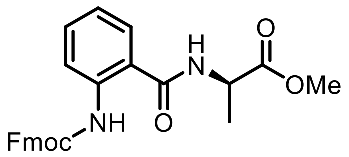

Methyl (2-((((9*H*-fluoren-9-yl)methoxy)carbonyl)amino)benzoyl)-ᴅ-alaninate (37): $[\alpha]_D^{25}$ -5.26 (*c* 11.4, $CHCl_3$). ^{1}H NMR (400 MHz, chloroform-*d*) δ 10.61 (s, 1H), 8.35 (d, *J* = 8.3 Hz, 1H), 7.78 (d, *J* = 7.5 Hz, 2H), 7.67 (d, *J* = 7.5 Hz, 2H), 7.56 (dd, *J* = 7.9, 1.3 Hz, 1H), 7.52 – 7.45 (m, 1H), 7.41 (t, *J* = 7.3 Hz, 2H), 7.36 – 7.29 (m, 2H), 7.10 – 7.02 (m, 1H), 6.88 (d, *J* = 6.8 Hz, 1H), 4.81 (p, *J* = 7.2 Hz, 1H), 4.47 – 4.39 (m, 2H), 4.31 (t, *J* = 7.5 Hz, 1H), 3.82 (s, 3H), 1.57 (d, *J* = 7.2 Hz, 3H). ^{13}C NMR (101 MHz, $CDCl_3$) δ 173.5, 168.5, 153.7, 144.00, 141.4, 140.0, 133.1, 127.8, 127.2, 126.9, 125.4, 122.2, 120.1, 119.5, 67.4, 52.8, 48.6, 47.1, 18.5. HRMS $(M+H)^+$ calcd. for $C_{26}H_{24}N_2O_5$ 445.1758, found 445.1759.

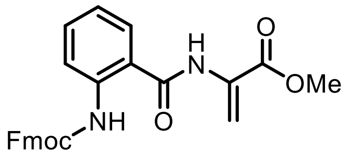

Methyl 2-(2-((((9*H*-fluoren-9-yl)methoxy)carbonyl)amino)benzamido)acrylate (38): ^{1}H NMR (400 MHz, chloroform-*d*) δ 10.51 (s, 1H), 8.59 (s, 1H), 8.39 (d, *J* = 8.4 Hz, 1H), 7.79 (d, *J* = 7.5 Hz, 2H), 7.68 (d, *J* = 7.4 Hz, 2H), 7.59 (dd, *J* = 7.9, 1.5 Hz, 1H), 7.53 (ddd, *J* = 8.6, 7.3, 1.5 Hz, 1H), 7.42 (td, *J* = 7.5, 1.2 Hz, 2H), 7.38 – 7.31 (m, 2H), 7.11 (ddd, *J* = 8.3, 7.4, 1.2 Hz, 1H), 6.82 (s, 1H), 6.08 (d, *J* = 1.2 Hz, 1H), 4.45 (d, *J* = 7.5 Hz, 2H), 4.37 – 4.29 (m, 1H), 3.91 (s, 3H). ^{13}C NMR (101 MHz, $CDCl_3$) δ 167.6, 164.7, 153.6, 143.9, 141.4, 140.2, 133.4, 130.9, 127.9, 127.2, 126.7, 125.4, 122.4, 120.6, 120.1, 119.9, 109.8, 67.5, 53.3, 47.1. HRMS $(M+Na)^+$ calcd. for $C_{26}H_{22}N_2O_5$ 465.1421, found 465.1441.

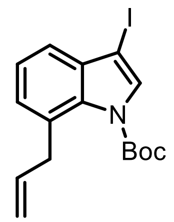

***tert*-Butyl 7-allyl-3-iodo-1*H*-indole-1-carboxylate (39):** To a solution of 7-allylindole (533 mg, 3.39 mmol) in DMF (12 mL) were added KOH pellets (2.5 equiv). A solution of iodine (1.0 equiv.) in DMF (8 mL) was added dropwise over 30 min under stirring. The reaction mixture was stirred at rt for 1 h, then poured into ice–water containing 0.5% NH_3 and 0.1% $Na_2S_2O_5$. The mixture was extracted with a 1:1 mixture of EtOAc/hexanes until no product remained in the aqueous phase. The combined organic layers were washed with cold water, dried over anhydrous Na_2SO_4, and concentrated in vacuo. The crude product was dissolved in CH_2Cl_2 (10 mL), and Boc_2O (1.2 equiv.), K_2CO_3 (1.2 equiv.), and DMAP (10 mol%) were added. The mixture was stirred at rt for 18 h and filtered. The crude product was purified by column chromatography (hexanes) to afford the title compound as a colorless oil (1.09 g, 85% yield). ^1H NMR (400 MHz, Chloroform-*d*) δ 7.67 (s, 1H), 7.36 – 7.20 (m, 3H), 5.99 – 5.85 (m, 1H), 5.05 – 4.91 (m, 2H), 3.91 (dt, *J* = 6.3, 1.7 Hz, 2H), 1.64 (s, 9H). ^{13}C NMR (101 MHz, $CDCl_3$) δ 148.5, 137.2, 133.7, 133.6, 132.3, 128.3, 127.5, 124.0, 120.2, 115.5, 84.1, 65.7, 39.1, 28.1. HRMS $(M+H)^+$ calcd for $C_{16}H_{18}INO_2$ 384.0455, found 384.0458.

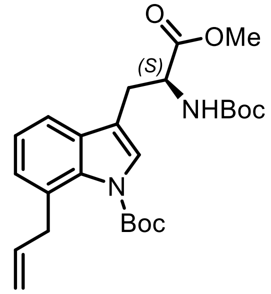

***tert*-Butyl (*S*)-7-allyl-3-(2-((*tert*-butoxycarbonyl)amino)-3-methoxy-3-oxopropyl)-1*H*-indole-1-carboxylate (40):** An oven-dried two-neck round-bottom flask was flame-dried twice under vacuum. After cooling to room temperature, Zn dust (132 mg, 1.98 mmol) was added under an argon atmosphere. The flask was evacuated and backfilled with argon three times. Dry DMF (6 mL) was added via syringe, followed by 1,2-dibromoethane (0.6 equiv.) to the stirred suspension. The mixture was stirred at 60 °C in an oil bath for 45 min, then cooled to rt. Chlorotrimethylsilane (0.2 equiv) was added via syringe (gas evolution observed), and the mixture was stirred for 40 min at rt. A solution of methyl (*R*)-2-((*tert*-butoxycarbonyl)amino)-3-iodopropanoate[6] (1.50 equiv) in dry DMF (3 mL) was added to the activated zinc suspension at rt, and the mixture was stirred at 35 °C for 1 h. The reaction mixture was cooled to rt, and under an argon atmosphere, $Pd_2(dba)_3$ (2.5 mol %) and 2-dicyclohexylphosphino-2′,6′-dimethoxybiphenyl (5 mol %) were added. A solution of *tert*-butyl 7-allyl-3-iodo-1*H*-indole-1-carboxylate (0.25 mmol, 1.0 equiv) in DMF (1 mL) was added immediately, and the mixture was stirred at 40 °C for 30 h. The reaction mixture

was filtered through a pad of Celite, diluted with EtOAc, washed with H_2O and brine, dried over anhydrous Na_2SO_4, filtered, and concentrated under reduced pressure. The product (108 mg, 95% yield) was purified by silica gel chromatography as a colorless liquid. ^{1}H NMR (400 MHz, Chloroform-*d*) δ 7.36 (d, *J* = 7.6 Hz, 1H), 7.31 (s, 1H), 7.23 – 7.13 (m, 2H), 5.93 (ddt, *J* = 16.7, 10.2, 6.3 Hz, 1H), 5.10 (d, *J* = 8.1 Hz, 1H), 5.04 – 4.91 (m, 2H), 4.64 (q, *J* = 6.4 Hz, 1H), 3.88 (dt, *J* = 6.4, 1.7 Hz, 2H), 3.68 (s, 3H), 3.19 (qd, *J* = 14.5, 5.5 Hz, 2H), 1.62 (s, 9H). ^{13}C NMR (101 MHz, $CDCl_3$) δ 172.5, 155.2, 149.4, 137.5, 134.4, 132.3, 127.6, 127.3, 126.5, 123.3, 117.2, 115.4, 114.9, 83.4, 80.0, 53.7, 52.4, 39.2, 28.4, 28.2, 27.9. HRMS $(M+H)^+$ calcd for $C_{25}H_{34}N_2O_6$ 459.2489, found 459.2485.

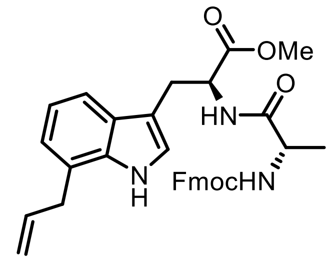

Methyl (*S*)-2-((*S*)-2-((((9H-fluoren-9-yl)methoxy)carbonyl)amino)propanamido)-3-(7-allyl-1H-indol-3-yl)propanoate (41): Trifluoroacetic acid (30 equiv.) was added via syringe to a solution of *tert*-butyl (*S*)-7-allyl-3-(2-((*tert*-butoxycarbonyl)amino)-3-methoxy-3-oxopropyl)-1*H*-indole-1-carboxylate (0.043 mmol, 1 equiv) in CH_2Cl_2 (3 mL) at rt. After 1.5 h, the brown solution was concentrated under reduced pressure to afford a viscous brown residue, which was dissolved in CH_2Cl_2 (5 mL) and cooled to 0 °C in an ice–water bath. Triethylamine (4.50 equiv) was added dropwise via syringe. HOBt (1.50 equiv) and Fmoc-L-Ala-OH (2.00 equiv) were sequentially added, followed by 1-ethyl-3-(3-dimethylaminopropyl)carbodiimide hydrochloride (1.50 equiv). The reaction mixture was allowed to warm to rt and stirred overnight. The reaction mixture was diluted with CH_2Cl_2 (20 mL) and washed with 1 N HCl (10 mL). The aqueous layer was extracted with CH_2Cl_2 (2 × 20 mL). The combined organic layers were washed with saturated $NaHCO_3$ (30 mL), and the resulting aqueous layer was extracted with CH_2Cl_2 (2 × 25 mL). The combined organic layers were dried over anhydrous Na_2SO_4, filtered, and concentrated. The product (20.3 mg, 86% yield over 2 steps) was purified by silica gel chromatography as a sticky liquid. ^{1}H NMR (400 MHz, Chloroform-*d*) δ 8.07 (s, 1H), 7.77 (d, *J* = 7.6 Hz, 2H), 7.58 (d, *J* = 7.6 Hz, 2H), 7.44 – 7.36 (m, 3H), 7.31 (t, *J* = 7.4 Hz, 2H), 7.03 (dd, *J* = 7.9, 7.1 Hz, 1H), 6.96 (d, *J* = 6.9 Hz, 2H), 6.43 (d, *J* = 8.0 Hz, 1H), 6.01 (ddt, *J* = 16.7, 10.0, 6.5 Hz, 1H), 5.28 (d, *J* = 7.8 Hz, 1H), 5.23 – 5.08 (m, 2H), 4.91 (dt, *J* = 7.9, 5.3 Hz, 1H), 4.33 (dt, *J* = 17.7, 9.0 Hz, 2H), 4.19 (t, *J* = 7.3 Hz, 2H), 3.69 (s, 3H), 3.55 (d, *J* = 6.5 Hz, 2H), 3.32 (d, *J* = 5.3 Hz, 2H), 1.34 (d, *J* = 7.0 Hz, 3H). ^{13}C NMR (101 MHz, $CDCl_3$) δ 172.1, 171.9, 155.9, 143.9, 141.4, 136.6, 135.6, 127.9, 127.8, 127.2, 125.2, 122.9, 122.5, 122.4, 120.2, 120.1, 117.0, 116.6, 110.1, 67.2, 53.6, 52.6, 50.6, 47.2, 36.7, 27.7, 18.8. HRMS $(M+H)^+$ calcd for $C_{33}H_{33}N_3O_5$ 552.24930, found 552.24858.

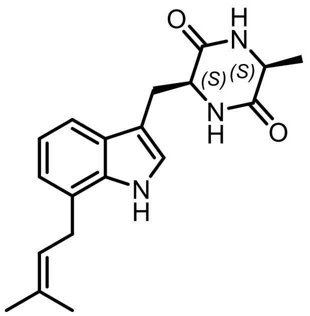

(3*S*,6*S*)-3-Methyl-6-((7-(3-methylbut-2-en-1-yl)-1*H*-indol-3-yl)methyl)piperazine-2,5-dione (6)[7]**:** To a solution of methyl (*S*)-2-((*S*)-2-((((9*H*-fluoren-9-yl)methoxy)carbonyl)amino)propanamido)-3-(7-allyl-1*H*-indol-3-yl)propanoate (30 mg, 0.0544 mmol, 1.0 equiv) in degassed CH_2Cl_2 (2 mL) was added 2-methyl-2-butene (30 equiv) at rt. Grubbs' 2nd generation catalyst (0.07 equiv.) was then added, and the reaction mixture was stirred at rt for 12 h. The solution was passed through a pad of Celite, and the solvent was removed in vacuo. The residue was dissolved in dry CH_2Cl_2 (0.8 mL), and piperidine (0.2 mL) was added to the stirred solution. The mixture was stirred for 12 h, after which the solvent was removed under reduced pressure. The crude product was purified by silica gel chromatography (5% MeOH/EtOAc) to afford the desired product as a white solid (70% yield, 12.3 mg). $[\alpha]_D^{25}$ +4.15 (*c* 5.3, MeOH) [lit. $[\alpha]_D^{24}$ +10.4 (*c* 0.11, MeOH)[7], $[\alpha]_D$ +7.0 (*c* 0.58, MeOH)[8]]. ^{1}H NMR (400 MHz, Methanol-d_4) δ 7.44 (dd, *J* = 7.9, 1.2 Hz, 1H), 7.07 (s, 1H), 6.93 (dd, *J* = 7.9, 7.1 Hz, 1H), 6.86 (dd, *J* = 7.1, 1.0 Hz, 1H), 5.39 (tp, *J* = 7.2, 1.4 Hz, 1H), 4.26 (td, *J* = 4.2, 1.3 Hz, 1H), 3.69 (qd, *J* = 7.0, 1.3 Hz, 1H), 3.52 (d, *J* = 7.2 Hz, 2H), 3.48 – 3.40 (m, 1H), 3.17 – 3.10 (m, 1H), 1.74 (q, *J* = 1.1 Hz, 6H), 0.36 (d, *J* = 7.1 Hz, 3H). ^{13}C NMR (101 MHz, MeOD) δ 170.6, 169.5, 136.5, 133.7, 129.2, 125.7, 125.6, 123.4, 121.7, 120.5, 117.8, 109.7, 57.5, 51.7, 30.9, 30.5, 25.9, 19.9, 17.9. HRMS $(M+H)^+$ calcd for $C_{19}H_{23}N_3O_2$ 326.1863, found 326.1867.

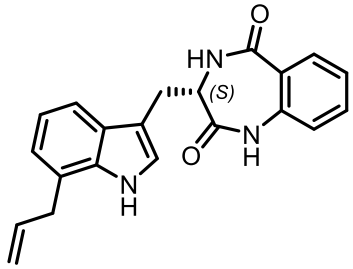

(*S*)-3-((7-Allyl-1*H*-indol-3-yl)methyl)-3,4-dihydro-1*H*-benzo[e][1,4] diazepine-2,5-dione (42): A solution of *tert*-butyl (S)-7-allyl-3-(2-((*tert*-butoxycarbonyl)amino)-3-methoxy-3-oxopropyl)-1*H*-indole-1-carboxylate (44.8 mg, 0.1 mmol) in MeOH (2 mL) at 0 °C was treated with 2 N aqueous KOH (100 µL). The reaction mixture was allowed to reach rt and stirred for 2 h. TLC indicated complete consumption of the starting material. The mixture was cooled to 0 °C, poured into 5% aqueous HCl (5 mL), and extracted with EtOAc (3 × 5 mL). The combined organic layers were dried over $MgSO_4$, filtered, and concentrated in vacuo. The crude residue was dissolved in CH_2Cl_2 (3 mL), and trifluoroacetic acid (164 µL, 30 equiv.) was added dropwise via syringe. The solution was stirred at rt for 1.5 h, then concentrated under reduced pressure to

afford a viscous brown residue. The resulting amino acid and isatoic anhydride (1.10 equiv.) were suspended in H_2O (2 mL). Triethylamine (3.0 equiv.) was added, and the mixture was stirred at rt for 12 h. The solvents were removed under reduced pressure, and glacial acetic acid (1.5 mL) was added. The mixture was heated at 120 °C for 8 h. After cooling, the solvent was removed under reduced pressure, and the residue was partitioned between EtOAc and H_2O (10 mL/10 mL). The organic phase was washed with saturated aqueous $NaHCO_3$, dried over Na_2SO_4, filtered, and concentrated. The crude product was purified by column chromatography to afford the title compound as a colorless oil (3.3 mg, 28% yield over 4 steps). ^1H NMR (500 MHz, Acetone-d_6) δ 10.01 (s, 1H), 9.46 (s, 1H), 7.80 – 7.76 (m, 1H), 7.52 (td, *J* = 7.5, 1.7 Hz, 1H), 7.46 (tt, *J* = 7.2, 3.2 Hz, 1H), 7.35 (d, *J* = 2.0 Hz, 1H), 7.22 (t, *J* = 7.3 Hz, 2H), 6.94 (q, *J* = 4.0, 3.5 Hz, 2H), 6.05 (ddt, *J* = 16.8, 10.0, 6.6 Hz, 1H), 5.16 (dq, *J* = 17.1, 1.8 Hz, 1H), 5.04 (dq, *J* = 10.0, 1.6 Hz, 1H), 4.24 – 4.17 (m, 1H), 3.63 (dt, *J* = 6.8, 1.6 Hz, 2H), 3.47 (dd, *J* = 15.2, 5.4 Hz, 1H), 3.22 (dd, *J* = 15.1, 9.2 Hz, 1H). ^{13}C NMR (126 MHz, Acetone) δ 172.4, 168.5, 137.8, 137.5, 136.4, 133.2, 131.7, 128.4, 124.9, 124.7, 123.8, 122.2, 121.8, 121.8, 120.0, 117.3, 116.0, 111.4, 53.6, 36.2, 25.01. HRMS $(M+H)^+$ calcd. for $C_{21}H_{19}N_3O_2$ 345.1477, found 345.1469.

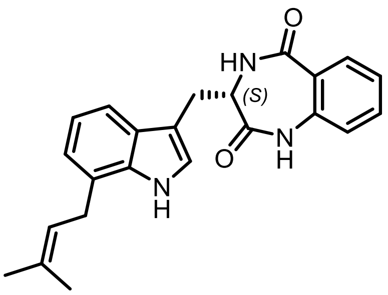

(*S*)-3-((7-(3-Methylbut-2-en-1-yl)-1*H*-indol-3-yl)methyl)-3,4-dihydro-1*H*-benzo[e][1,4]diazepine-2,5-dione (43): The compound was synthesized following general procedure F. (*S*)-3-((7-Allyl-1*H*-indol-3-yl)methyl)-3,4-dihydro-1*H*-benzo[e][1,4]diazepine-2,5-dione (3.0 mg, 8.69 μmol) was dissolved in dry CH_2Cl_2 (0.5 mL). To this stirring solution, Grubbs II catalyst (1.0 mg, 2 mol %) and 2-methyl-2-butene (0.1 mL) were added. The reaction mixture was stirred at rt for 12 h. Upon completion, the crude reaction mixture was filtered through a short pad of Celite, and the solvent was removed in vacuo. The crude product was purified by silica gel chromatography to afford (*S*)-3-((7-(3-methylbut-2-en-1-yl)-1*H*-indol-3-yl)methyl)-3,4-dihydro-1*H*-benzo[e][1,4]diazepine-2,5-dione (*ent*-asperdinone E) as a white solid (2.1 mg, 65% yield). $[\alpha]_D^{25}$ +69.5 (*c* 1.15, MeOH) [lit. Asperdinone E $[\alpha]_D^{25}$ -53.2 (*c* 0.1, MeOH)[5]];^1H NMR (500 MHz, Acetone-d_6) *δ* 9.93 (s, 1H), 9.42 (s, 1H), 7.78 (dd, *J* = 8.3, 1.6 Hz, 1H), 7.55 – 7.50 (m, 1H), 7.43 (dd, *J* = 5.4, 3.6 Hz, 1H), 7.34 (d, *J* = 2.5 Hz, 1H), 7.26 (d, *J* = 4.3 Hz, 1H), 7.22 (dd, *J* = 8.0, 6.7 Hz, 2H), 6.95 – 6.88 (m, 2H), 5.43 (tdt, *J* = 7.3, 2.9, 1.4 Hz, 1H), 4.20 (ddd, *J* = 9.2, 6.2, 4.7 Hz, 1H), 3.58 (d, *J* = 7.2 Hz, 2H), 3.47 (ddd, *J* = 15.1, 5.4, 1.0 Hz, 1H), 3.21 (dd, *J* = 15.1, 9.3 Hz, 1H), 1.78 – 1.72 (m, 6H). ^{13}C NMR (126 MHz, Acetone) δ 172.4, 168.4, 137.8, 136.4, 133.3, 133.2, 131.7, 128.3, 127.4, 125.3, 124.9, 124.8, 123.0, 121.8, 121.6, 120.0, 116.9, 111.4, 53.6, 30.3, 25.8, 25.0, 17.9. HRMS $(M+H)^+$ calcd. for $C_{23}H_{23}N_3O_2$ 374.1863, found 374.1862.

4. References:

1) Shi, S.; Yuan, K.; Jia, Y. *Chin. Chem. Lett.* **2020**, *31*, 401–403. doi:10.1016/j.cclet.2019.06.048
2) Kuttruff, C. A.; Zipse, H.; Trauner, D. *Angew. Chem. Int. Ed.* **2011**, *50*, 1402–1405. doi:10.1002/anie.201006154
3) Leonori, D.; Coldham, I. *Adv. Synth. Catal.* **2009**, *351*, 2619–2623. doi:10.1002/adsc.200900499
4) Kotha, S.; Meher, P. *Asian J. Org. Chem*. **2023**, *12*, e202300287. doi:10.1002/ajoc.202300287.

5) Lu, P.; Shi, Y.; Zhang, J.; Hong, K.; Xue, Y.; Liu, L. *Int. J. Biol. Macromol*. **2024**, *257*, 128808. doi:10.1016/j.ijbiomac.2023.128808
6) Trost, B. M.; Rudd, M. T.; *Org. Lett.* **2003**, *5*, 4599–4602. doi: 10.1021/ol035752n
7) Tanaka, S.; Shiomi, S.; Ishikawa, H. *J. Nat. Prod.* **2017**, *80*, 2371–2378. doi:10.1021/acs.jnatprod.7b00464
8) Wang, Y.; Gloer, J. B.; Scott, J. A.; Malloch, D. *J. Nat. Prod.* **1995**, *58*, 93–99. 216 doi:10.1021/np50115a011

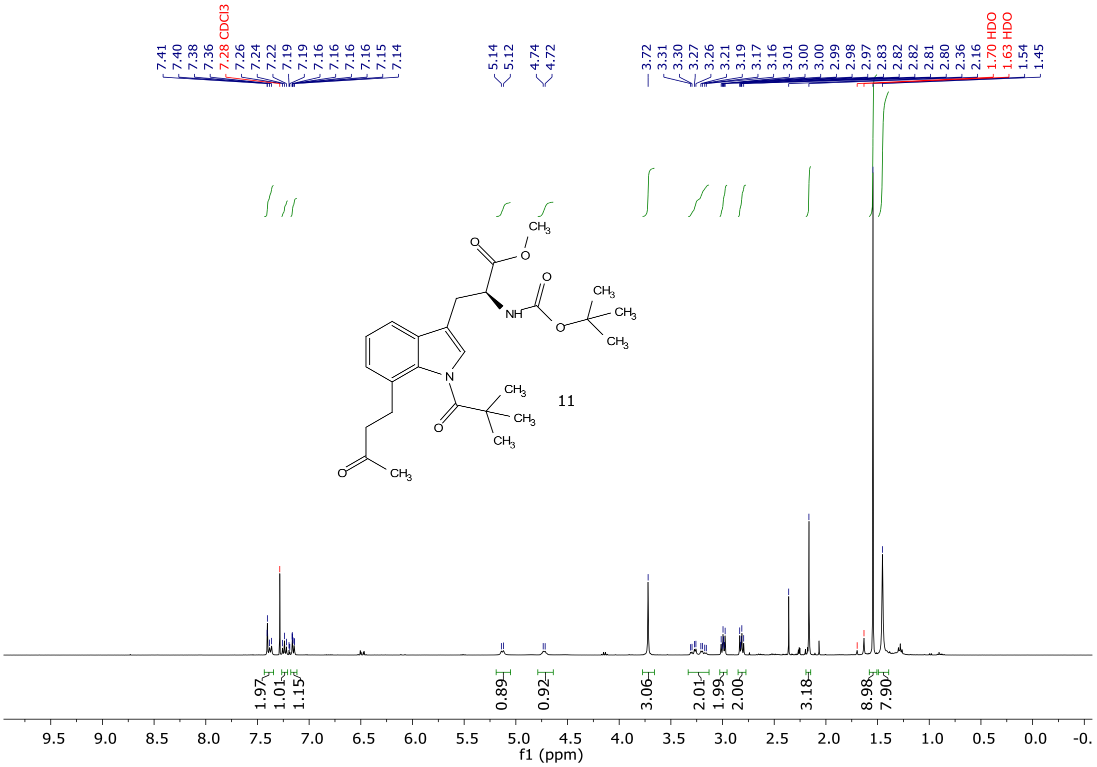

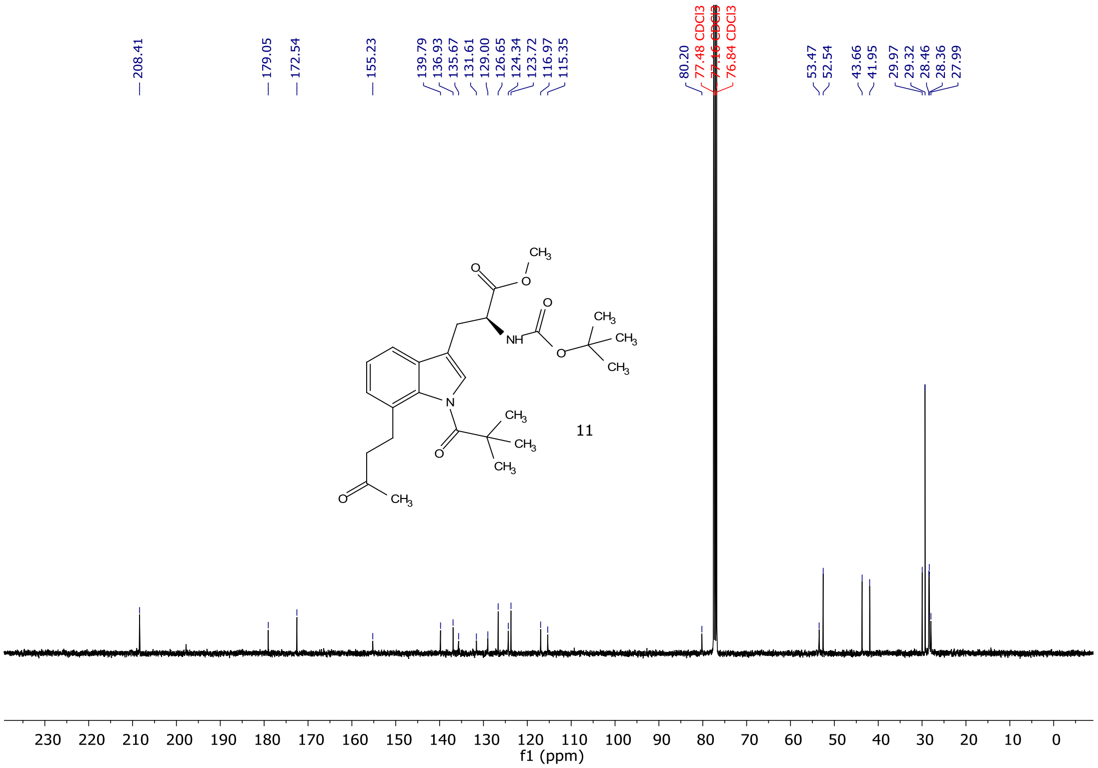

Figure S1: ^{1}H NMR (400 MHz, $CDCl_3$) spectrum (top) and ^{13}C {^{1}H} NMR (101 MHz, $CDCl_3$) spectrum (bottom) for **11**.

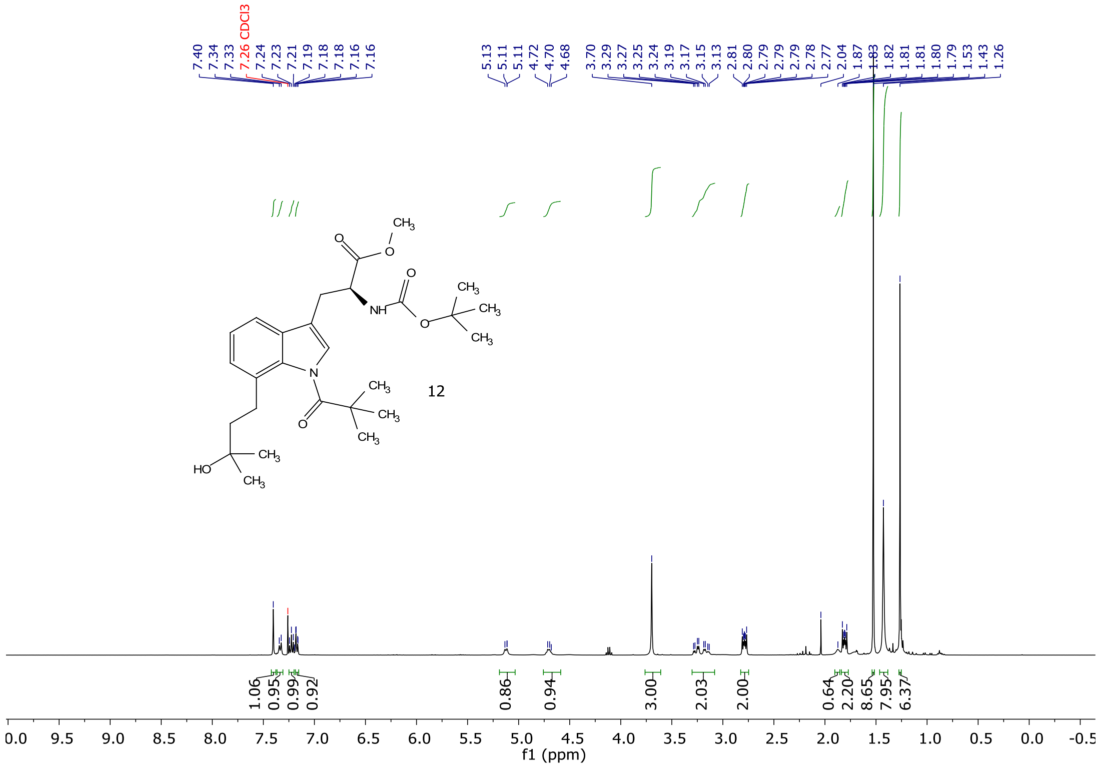

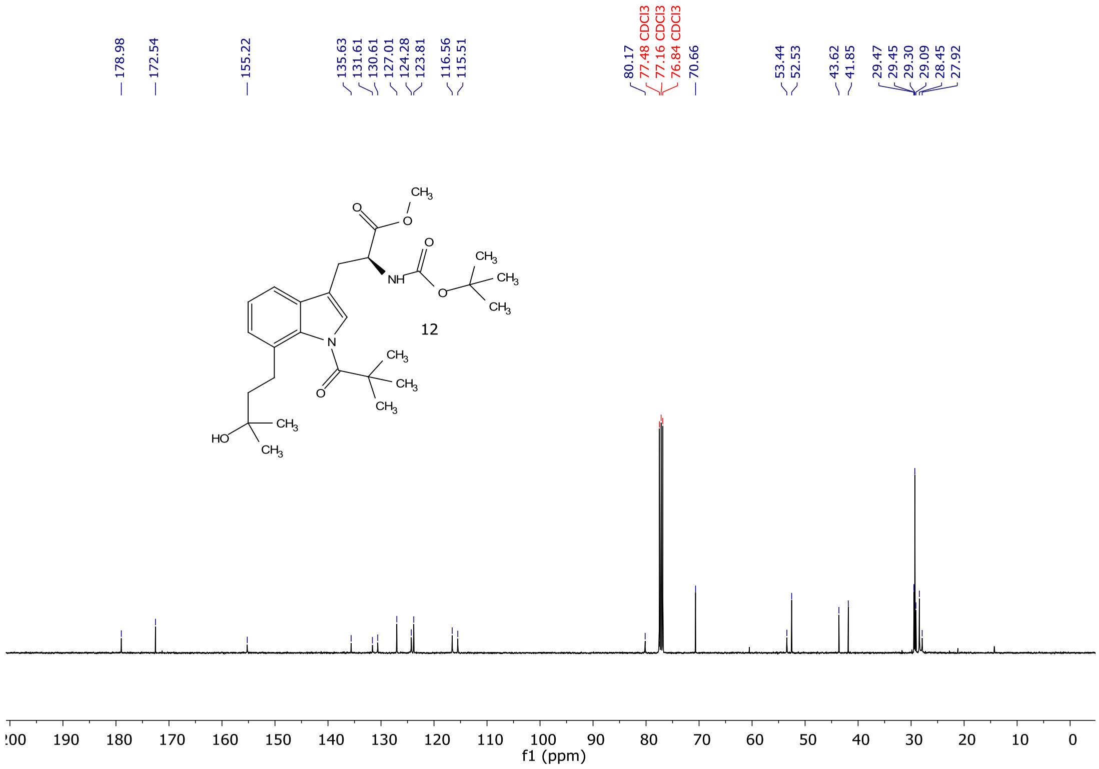

Figure S2: ^{1}H NMR (400 MHz, $CDCl_3$) spectrum (top) and ^{13}C {^{1}H} NMR (101 MHz, $CDCl_3$) spectrum (bottom) for **12**.

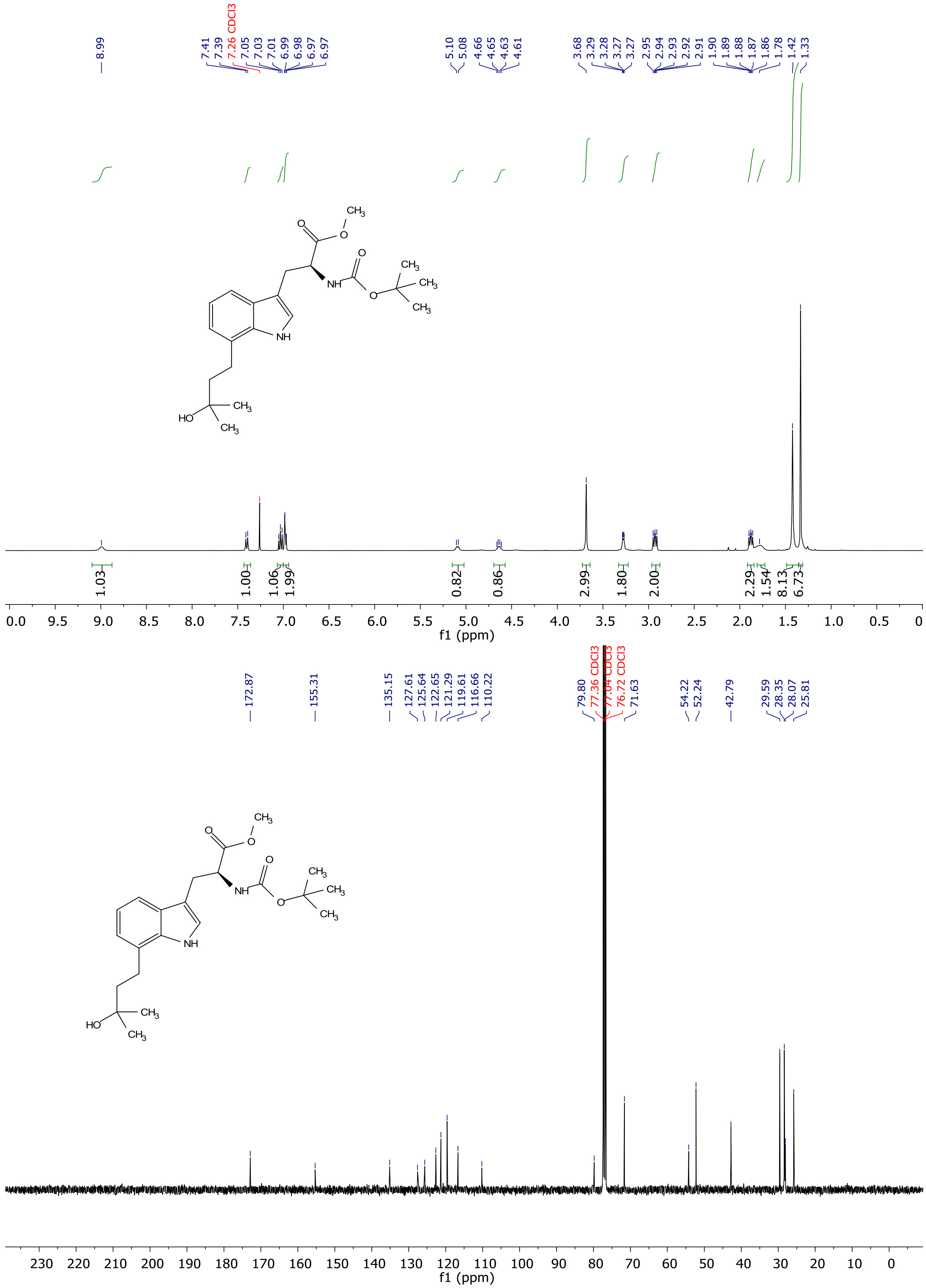

Figure S3: ^{1}H NMR (400 MHz, $CDCl_3$) spectrum (top) and $^{13}C\{^1H\}$ NMR (101 MHz, $CDCl_3$) spectrum (bottom) for methyl (*S*)-2-((*tert*-butoxycarbonyl)amino)-3-(7-(3-hydroxy-3-methylbutyl)-1*H*-indol-3-yl)propanoate.

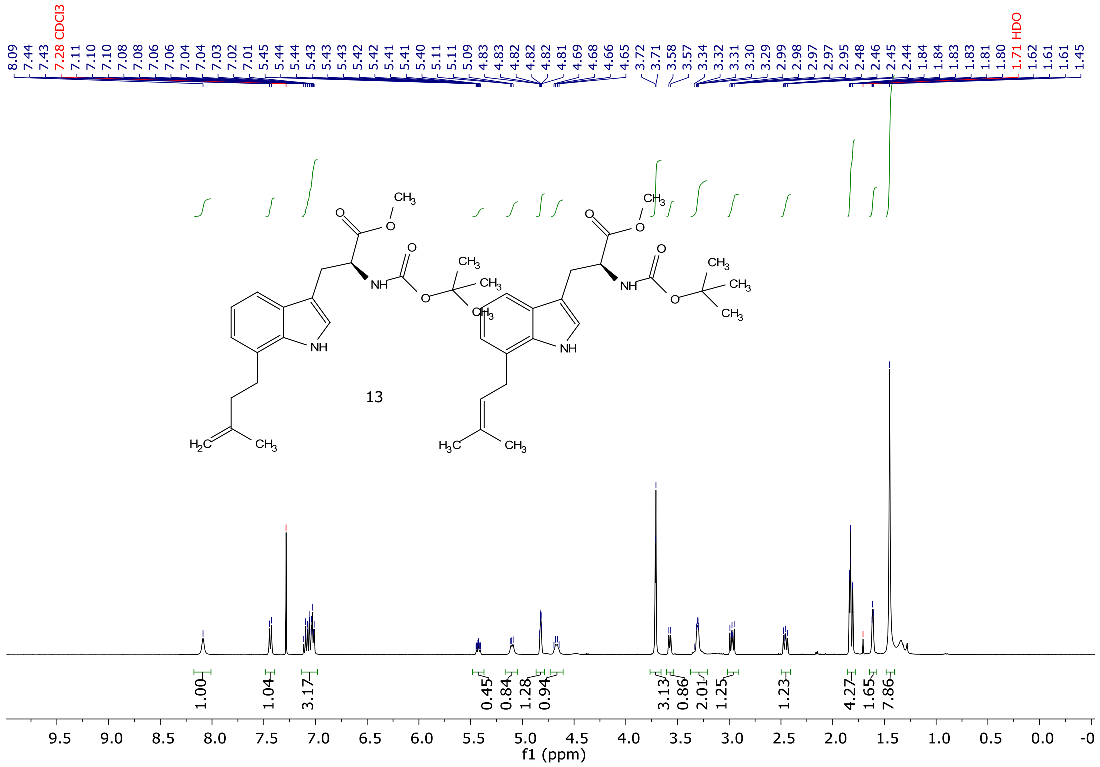

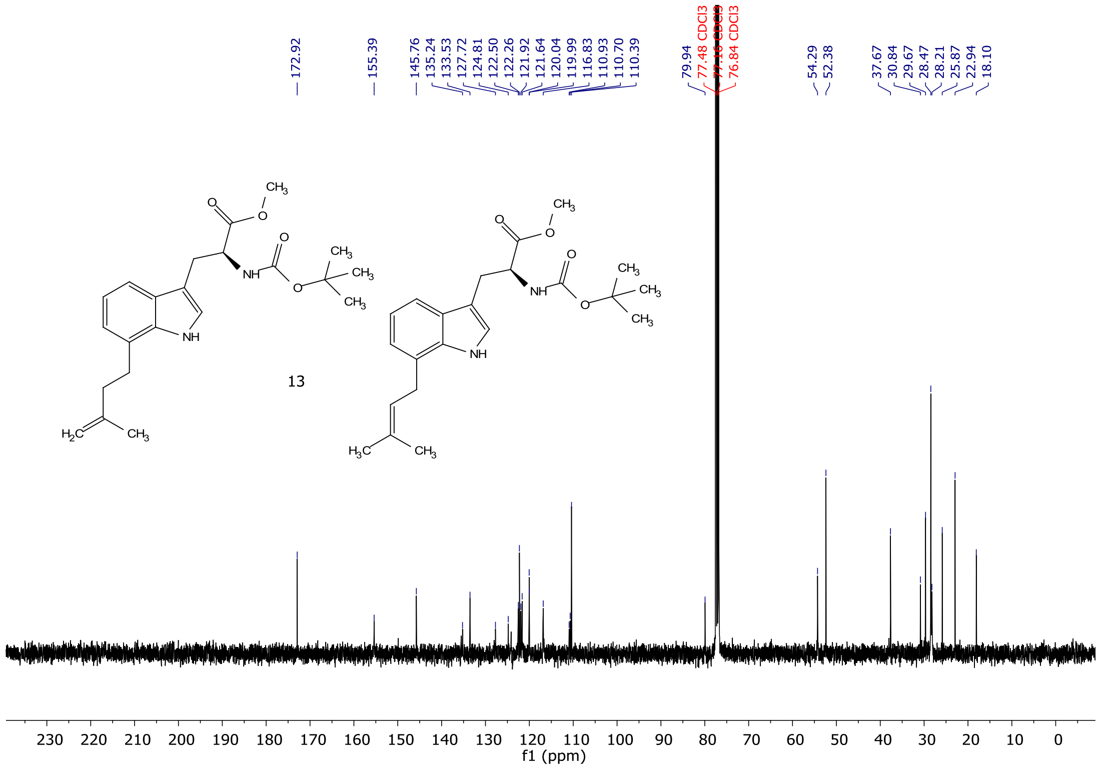

Figure S4: ^{1}H NMR (400 MHz, $CDCl_3$) spectrum (top) and ^{13}C {^{1}H} NMR (101 MHz, $CDCl_3$) spectrum (bottom) for **13**.

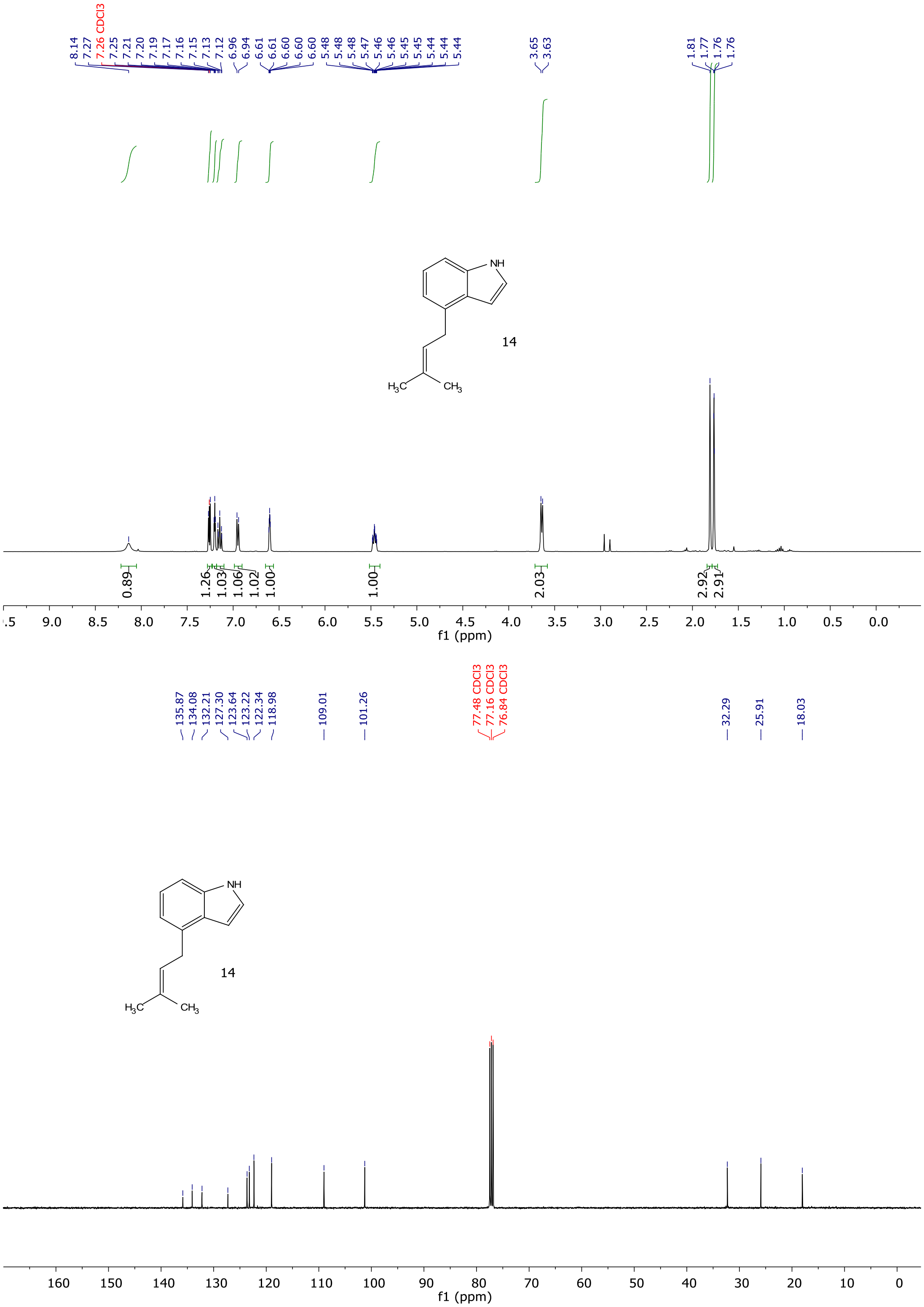

Figure S5: ^{1}H NMR (400 MHz, $CDCl_3$) spectrum (top) and ^{13}C {^{1}H} NMR (101 MHz, $CDCl_3$) spectrum (bottom) for **14**.

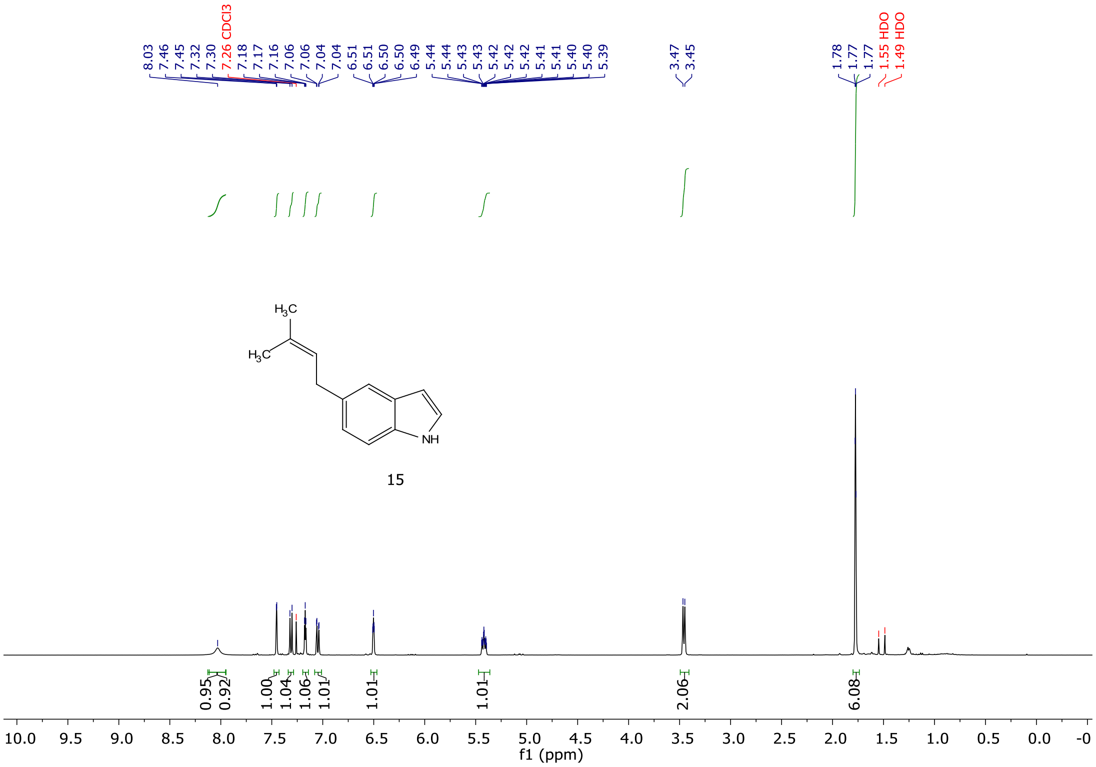

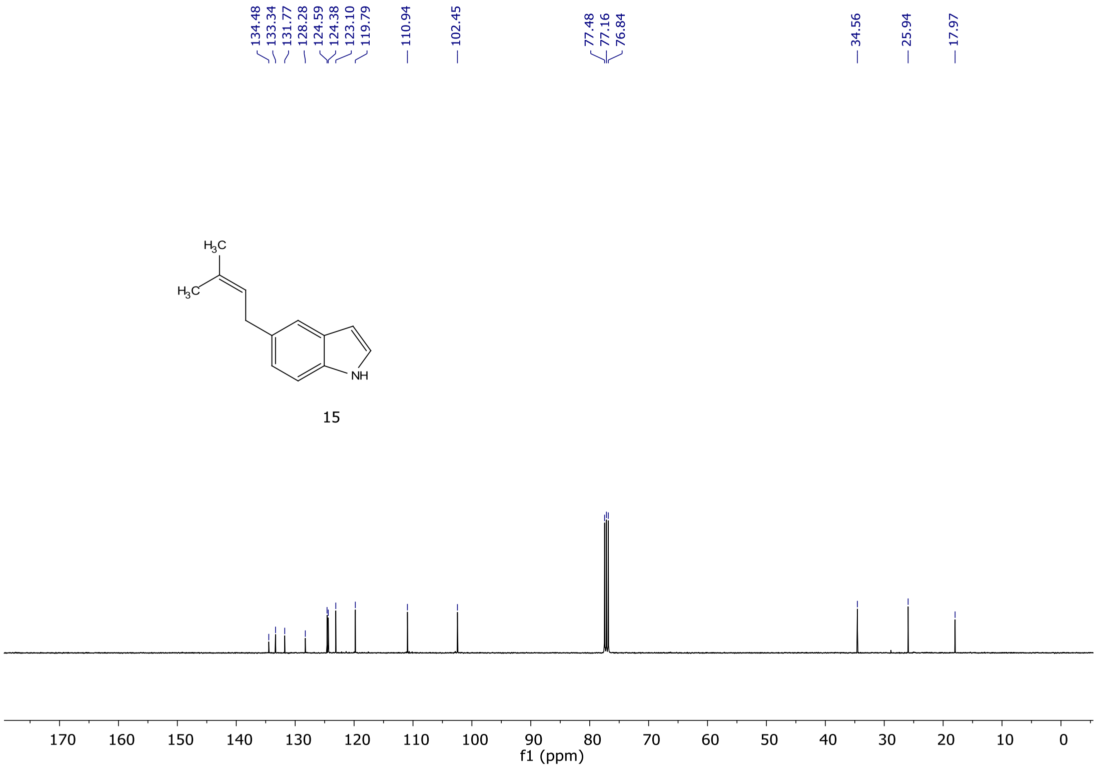

Figure S6: ^{1}H NMR (400 MHz, $CDCl_3$) spectrum (top) and ^{13}C {^{1}H} NMR (101 MHz, $CDCl_3$) spectrum (bottom) for **15**.

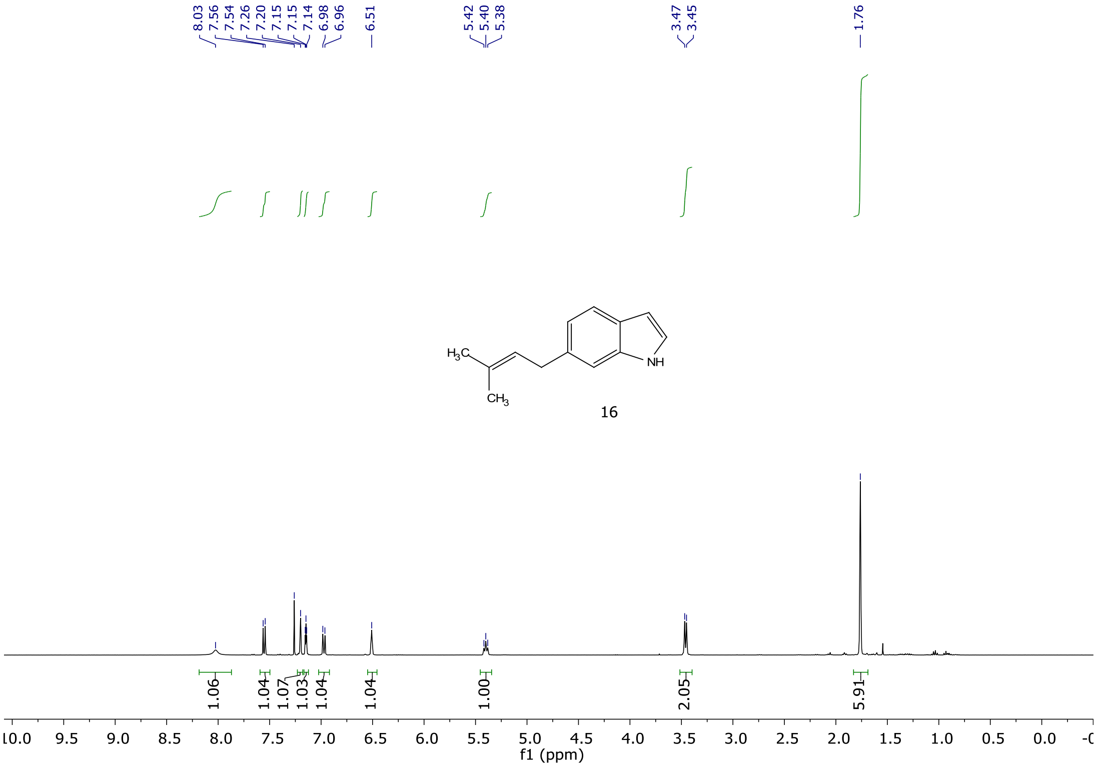

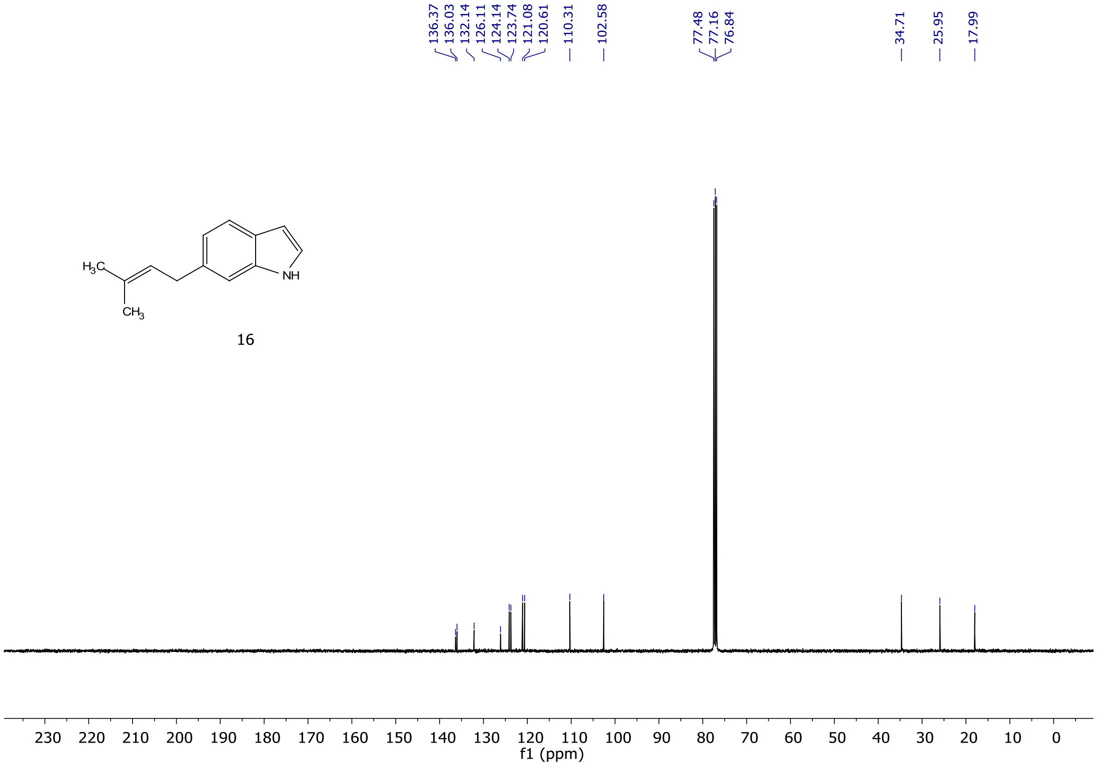

Figure S7: ^{1}H NMR (400 MHz, $CDCl_3$) spectrum (top) and ^{13}C {^{1}H} NMR (101 MHz, $CDCl_3$) spectrum (bottom) for **16**.

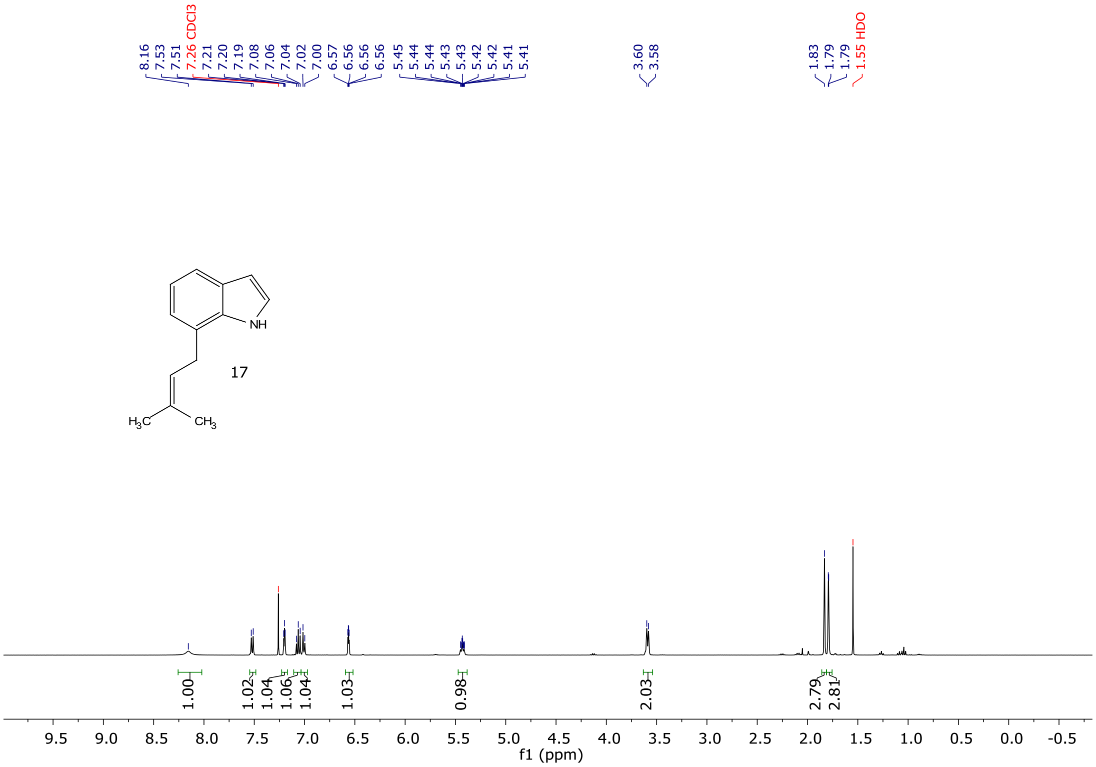

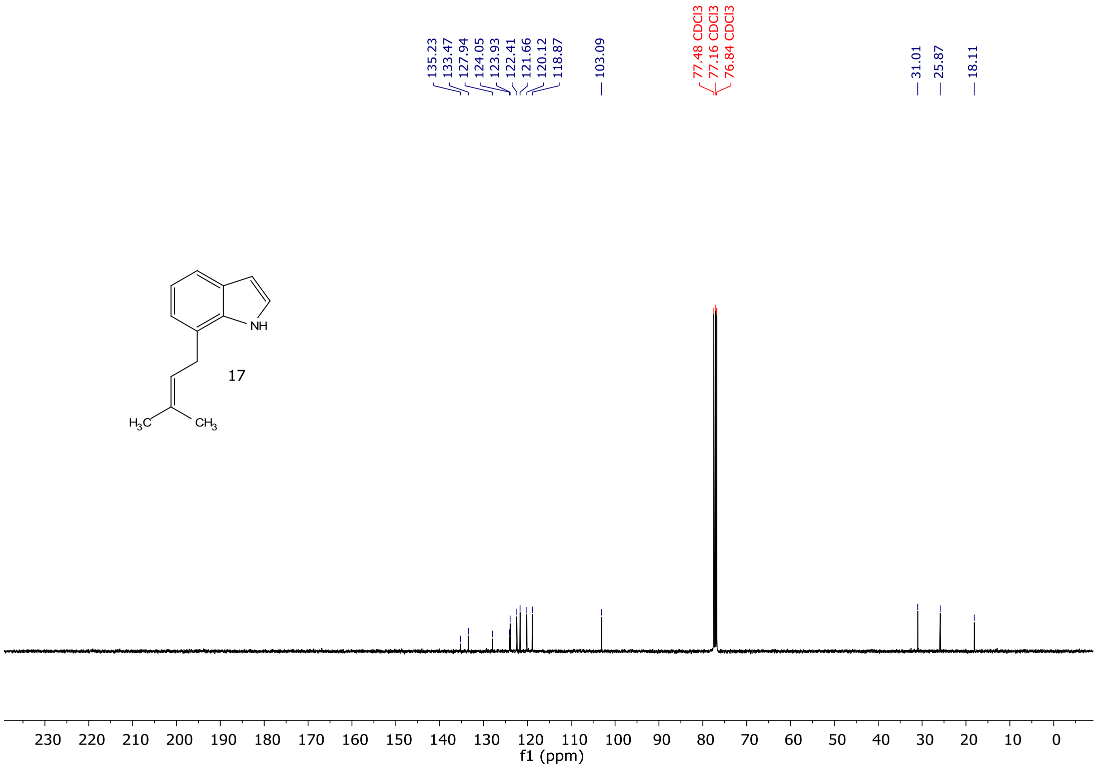

Figure S8: ^{1}H NMR (400 MHz, $CDCl_3$) spectrum (top) and ^{13}C {^{1}H} NMR (101 MHz, $CDCl_3$) spectrum (bottom) for **17**.

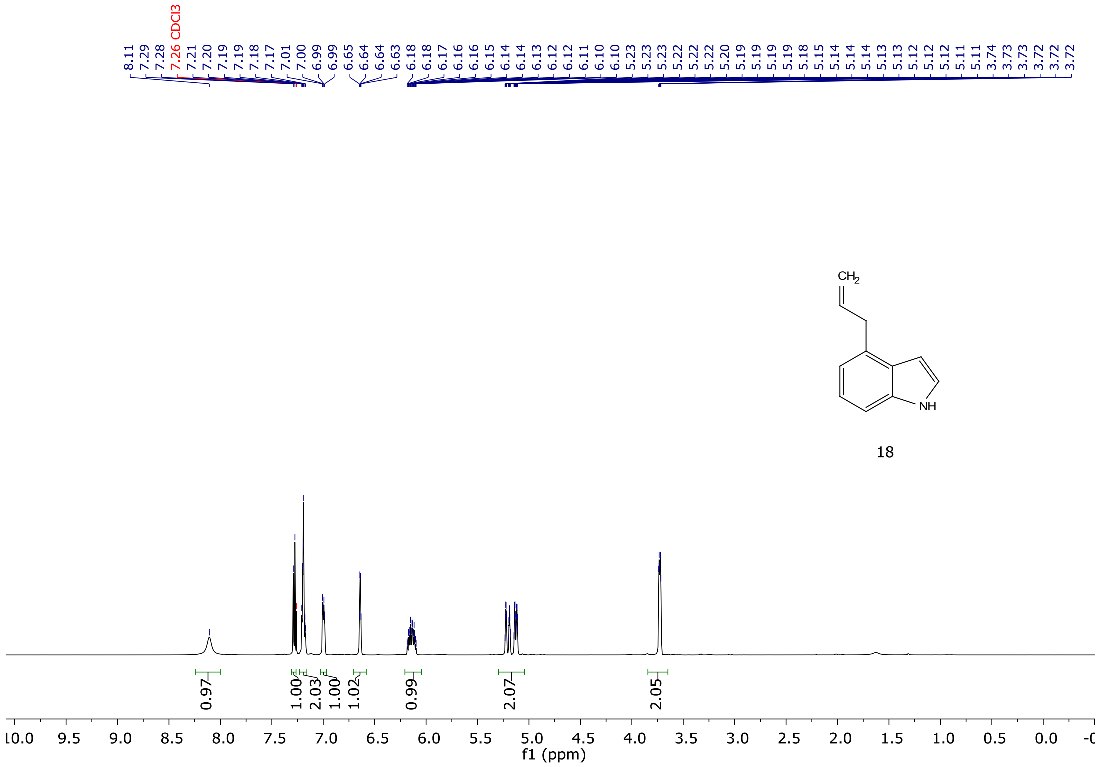

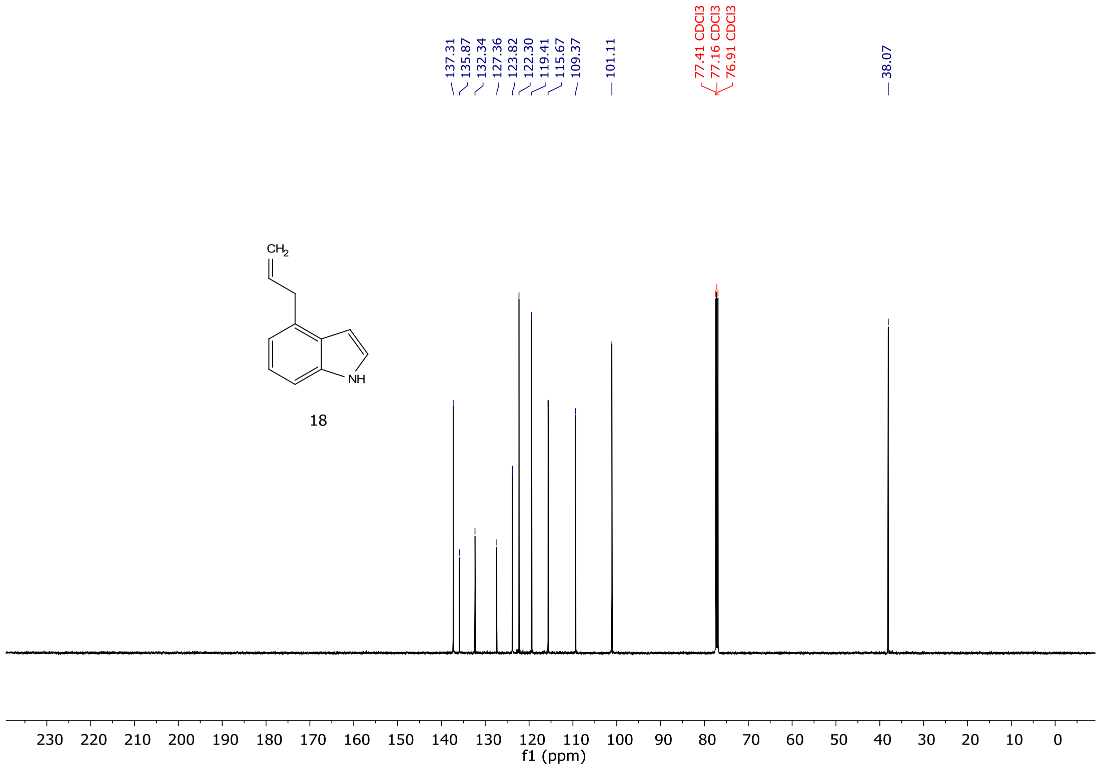

Figure S9: ^{1}H NMR (400 MHz, $CDCl_3$) spectrum (top) and ^{13}C {^{1}H} NMR (101 MHz, $CDCl_3$) spectrum (bottom) for **18**.

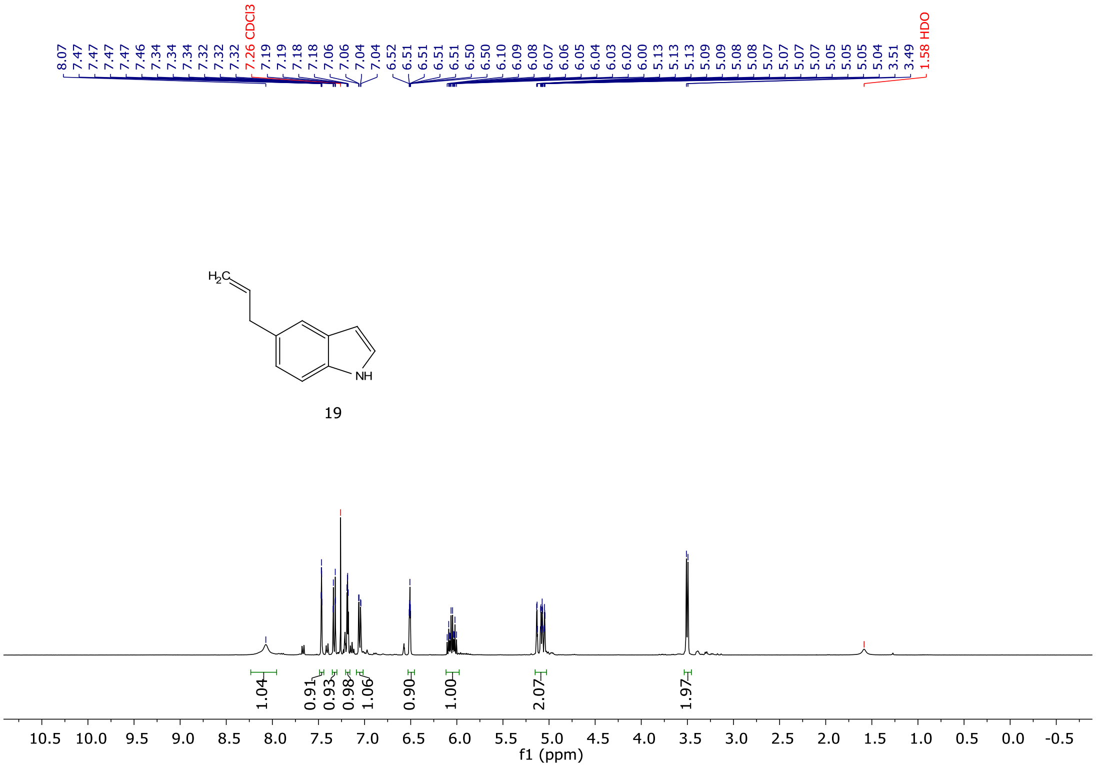

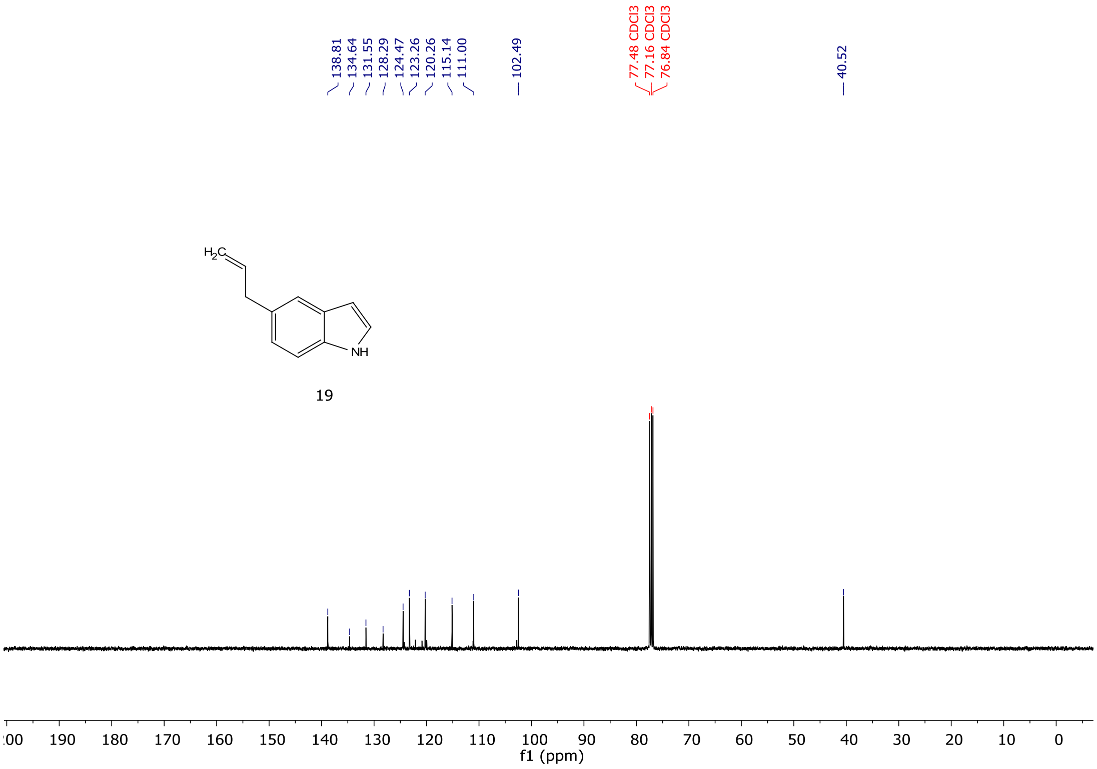

Figure S10: ^{1}H NMR (500 MHz, $CDCl_3$) spectrum (top) and ^{13}C {^{1}H} NMR (126 MHz, $CDCl_3$) spectrum (bottom) for **19**.

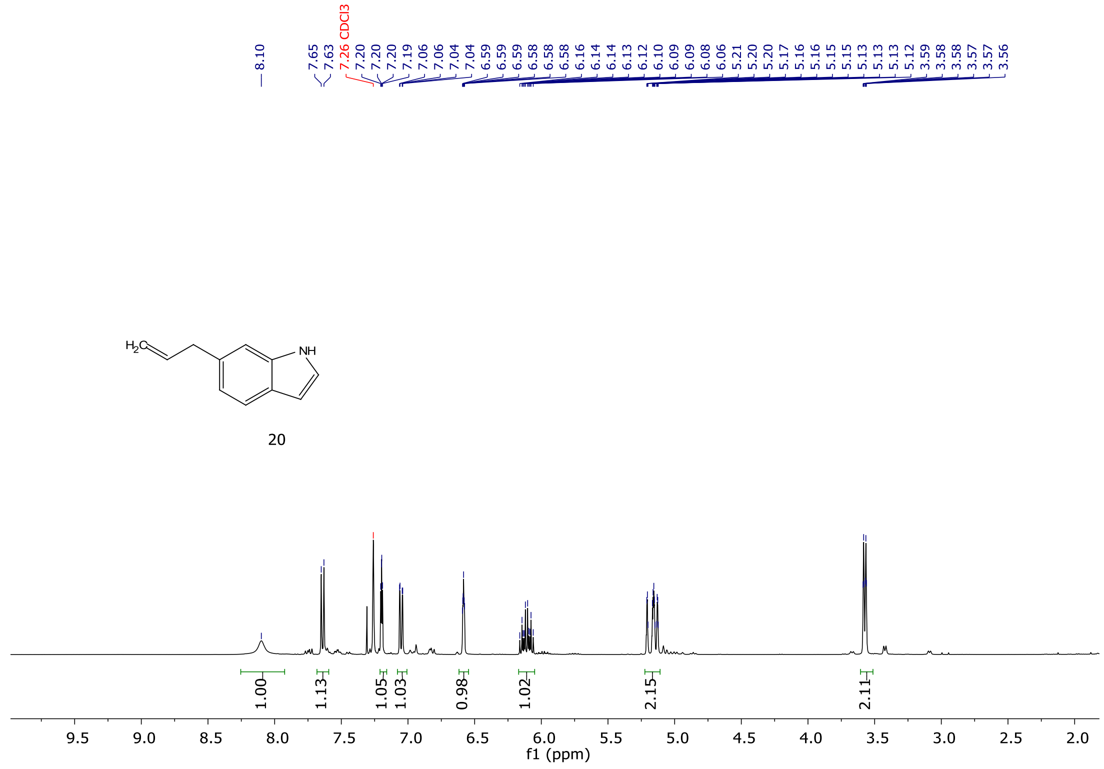

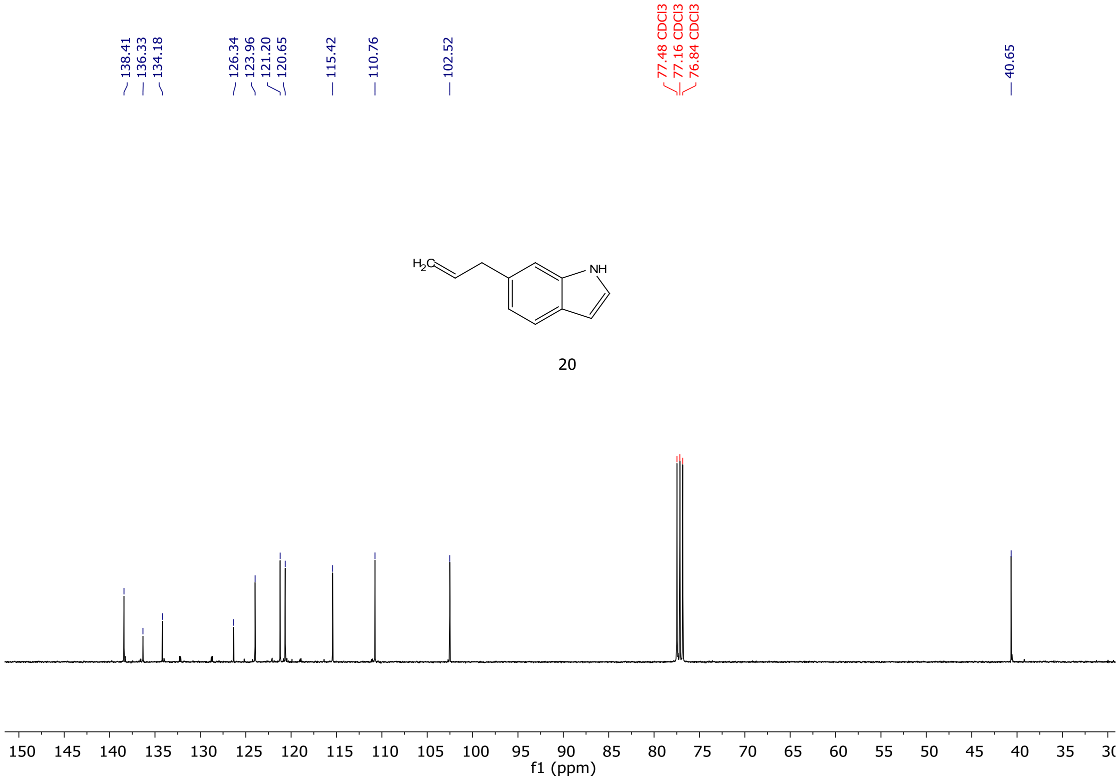

Figure S11: ^{1}H NMR (400 MHz, $CDCl_3$) spectrum (top) and ^{13}C {^{1}H} NMR (101 MHz, $CDCl_3$) spectrum (bottom) for **20**.

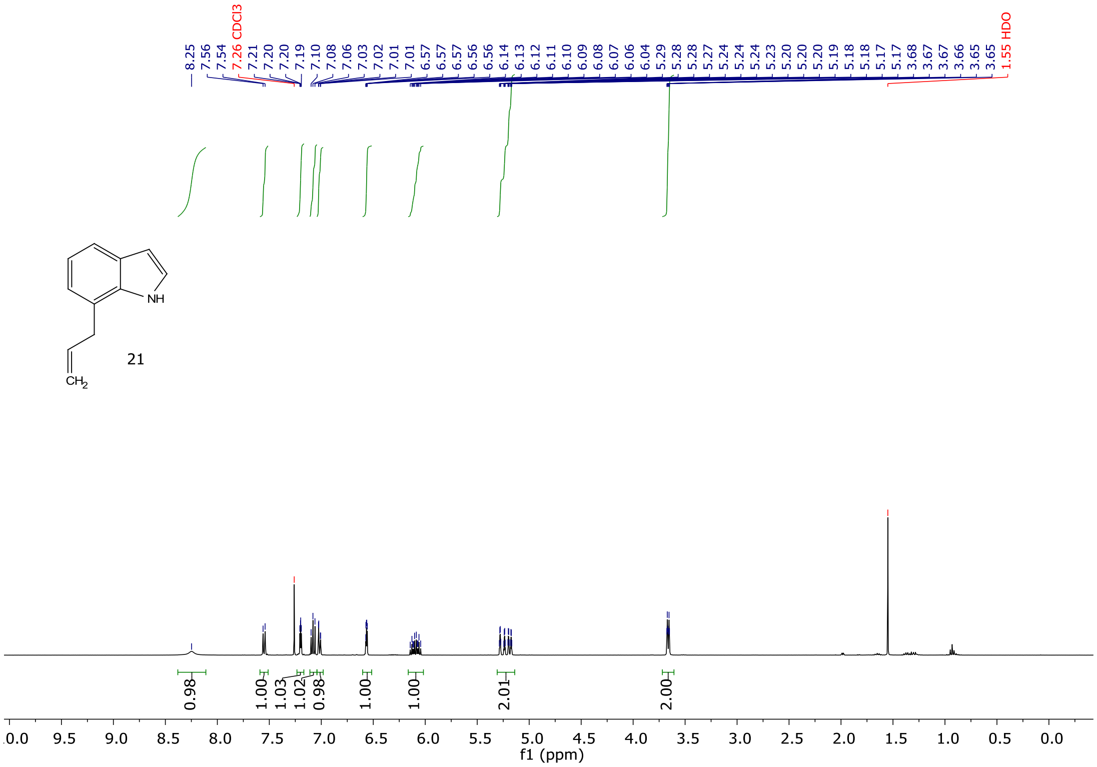

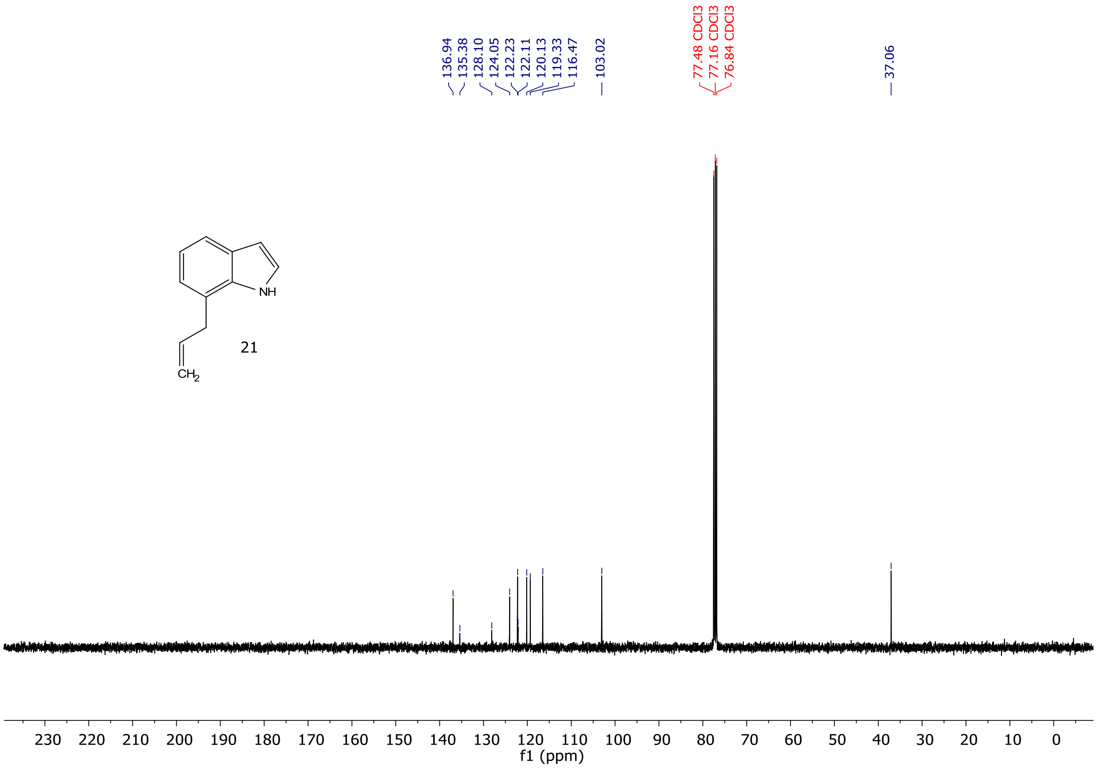

Figure S12: ^{1}H NMR (400 MHz, $CDCl_3$) spectrum (top) and ^{13}C {^{1}H} NMR (101 MHz, $CDCl_3$) spectrum (bottom) for **21**.

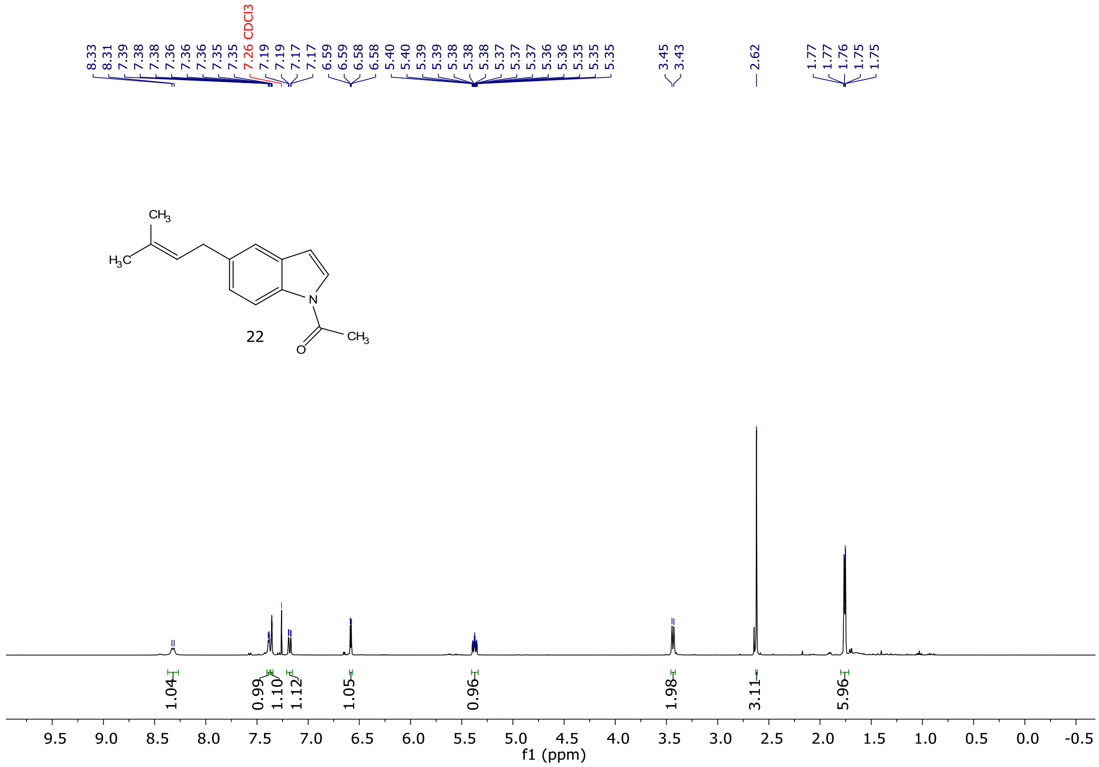

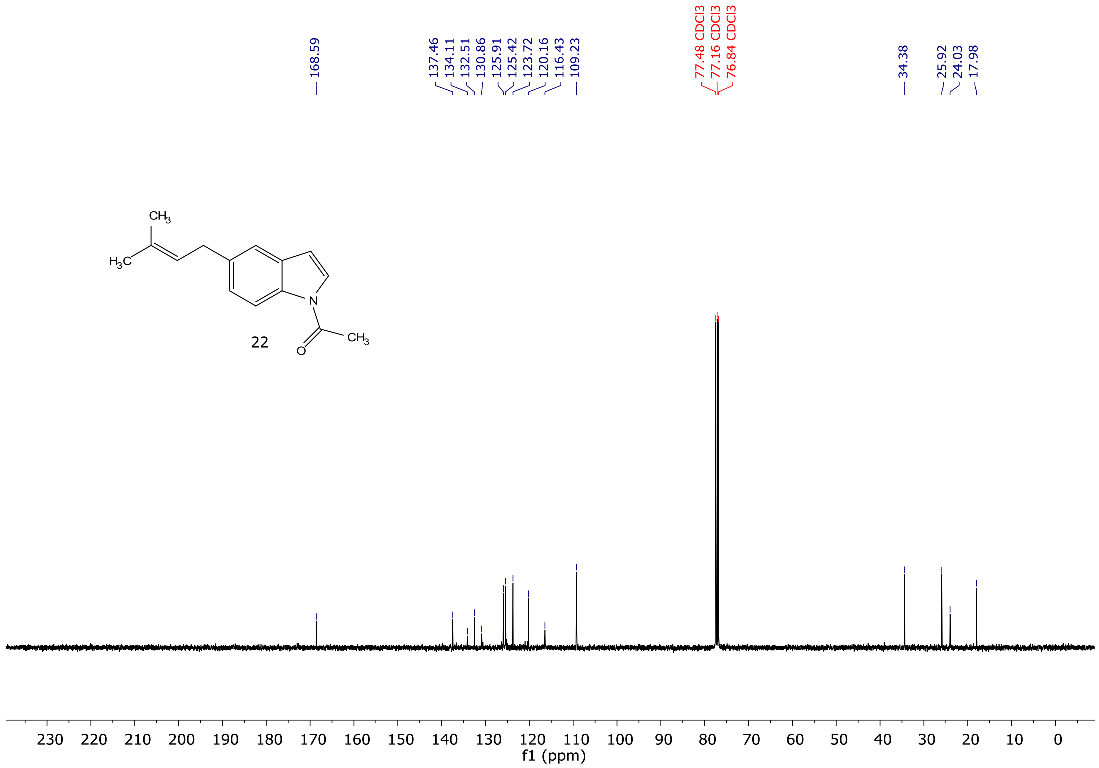

Figure S13: ^{1}H NMR (400 MHz, $CDCl_3$) spectrum (top) and $^{13}C\{^{1}H\}$ NMR (101 MHz, $CDCl_3$) spectrum (bottom) for **22**.

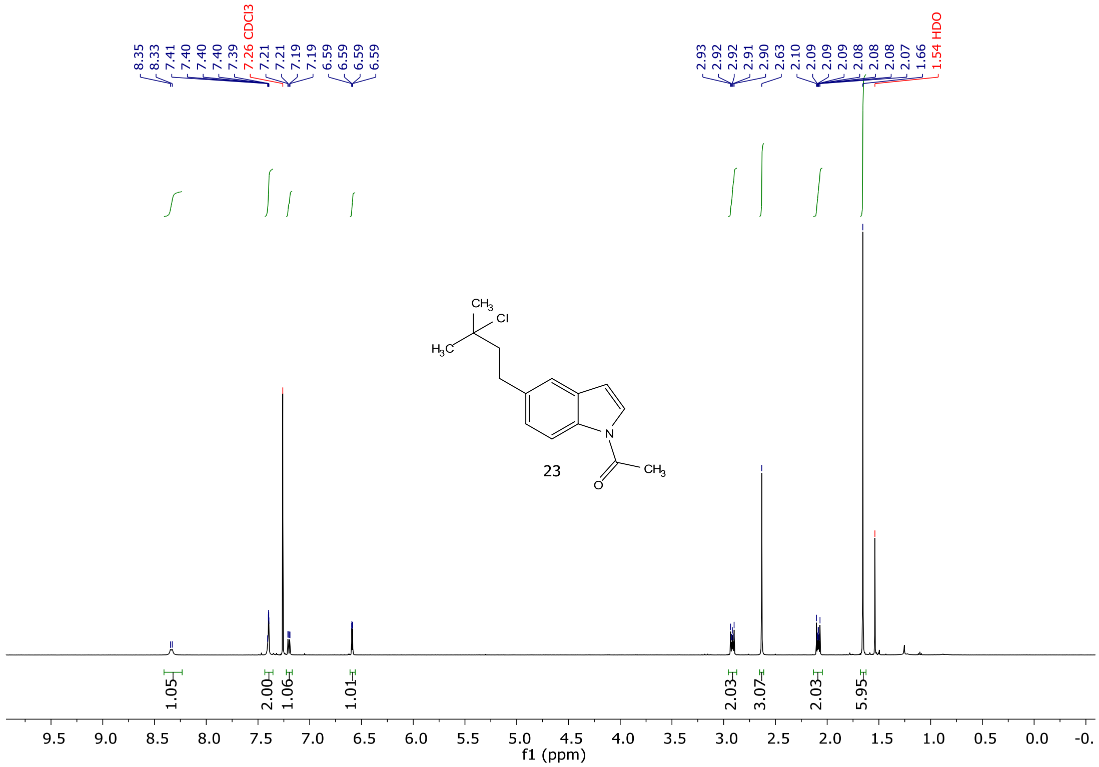

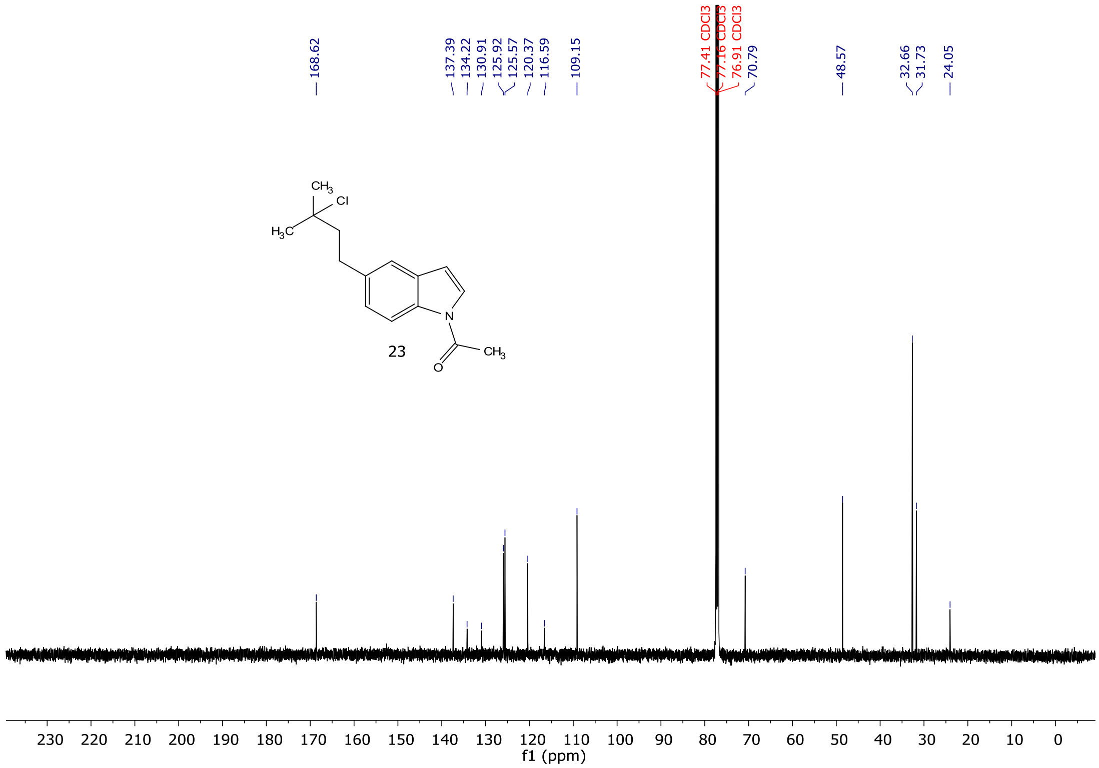

Figure S14: ^{1}H NMR (500 MHz, $CDCl_3$) spectrum (top) and $^{13}C\{^{1}H\}$ NMR (126 MHz, $CDCl_3$) spectrum (bottom) for **23**.

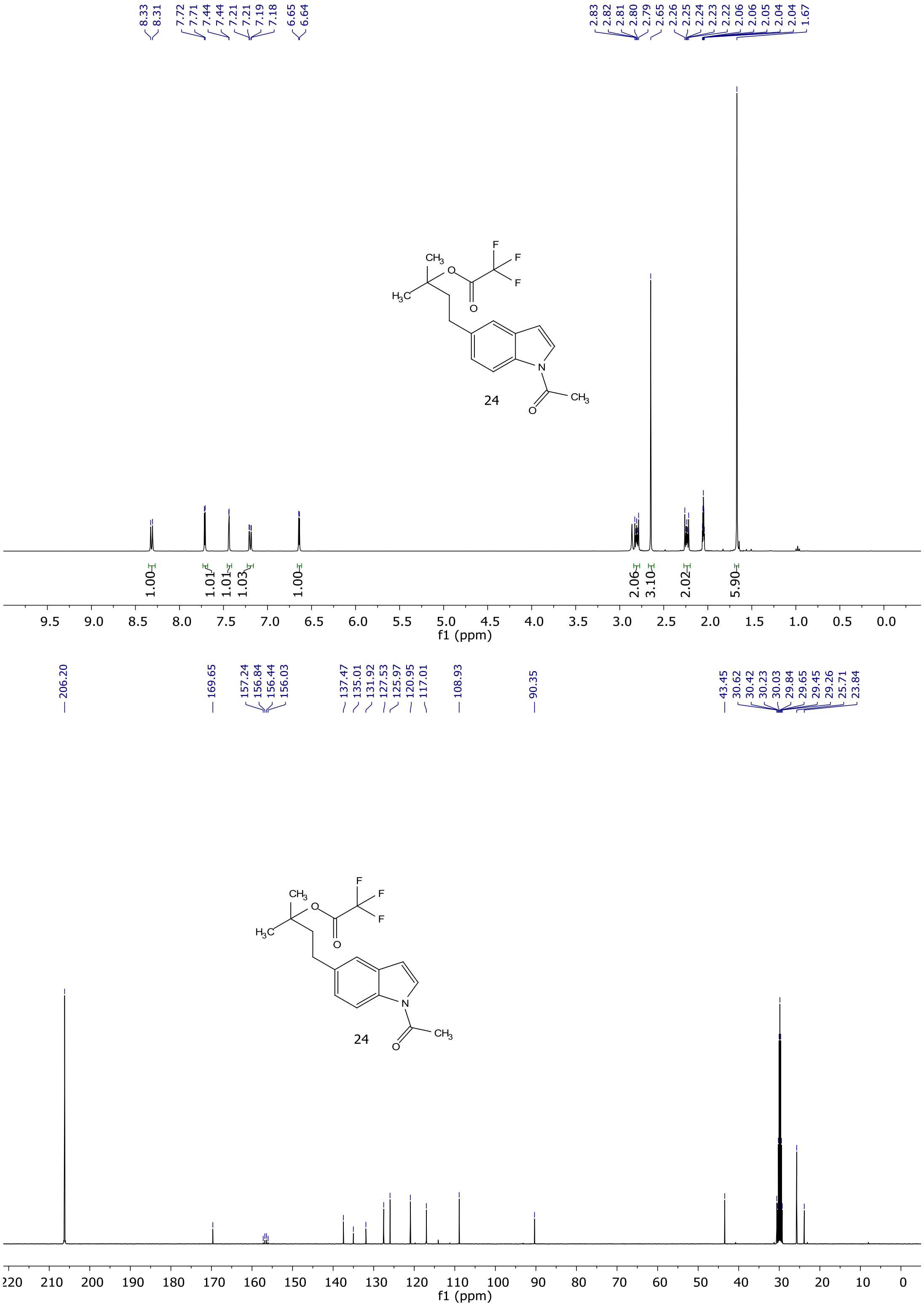

Figure S15: ^{1}H NMR (400 MHz, acetone-d_6) spectrum (top) and ^{13}C {^{1}H} NMR (101 MHz, acetone-d_6) spectrum (bottom) for **24**.

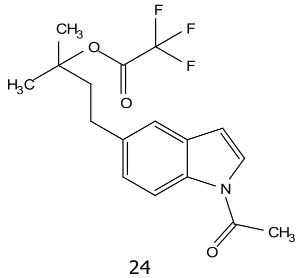

24

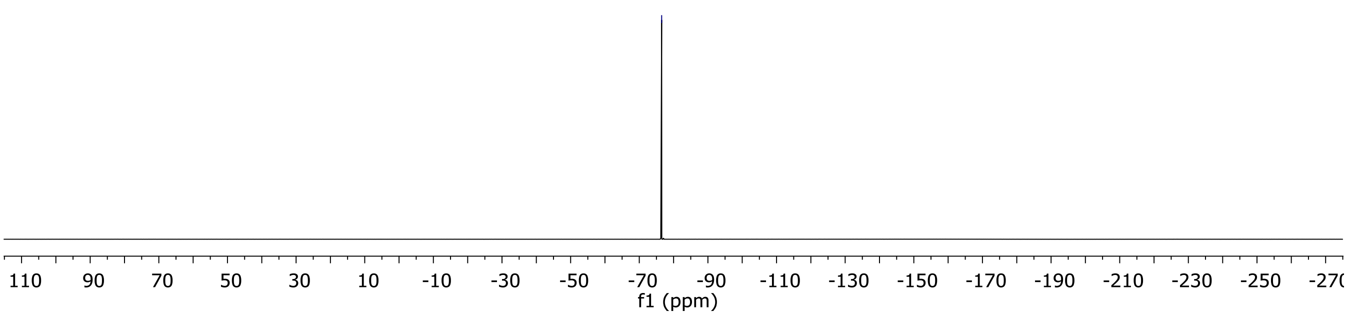

Figure S16:^{19}F NMR (377 MHz, acetone) spectrum for **24**.

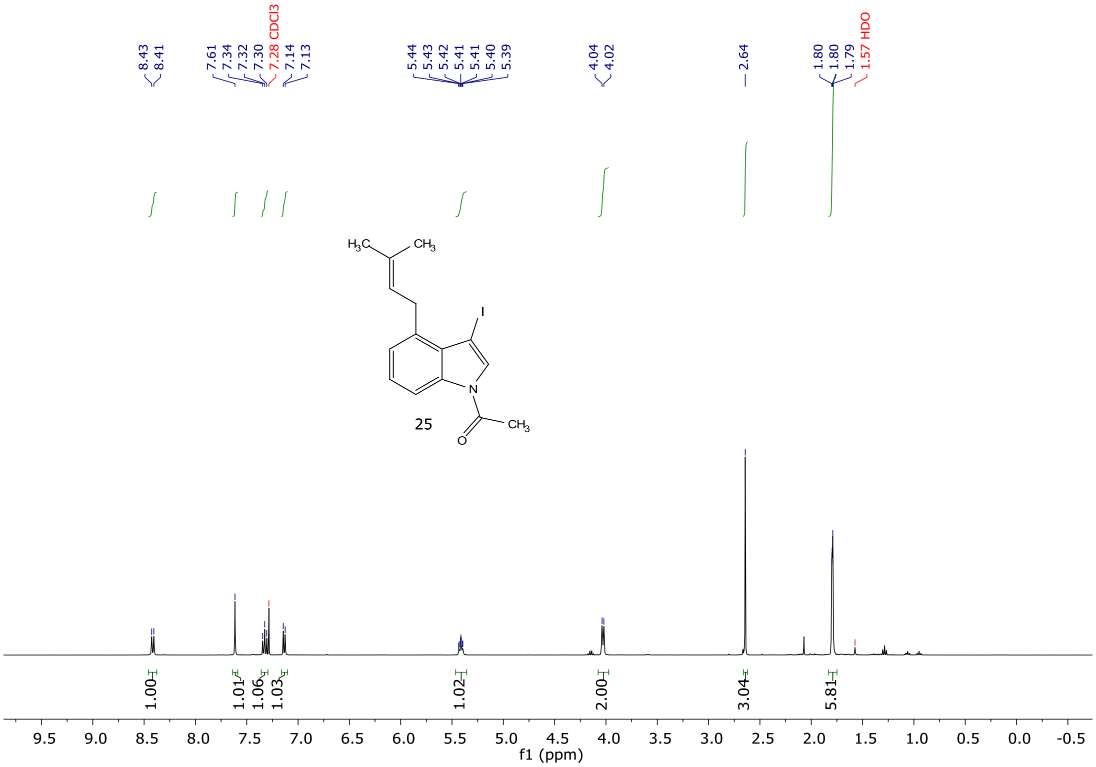

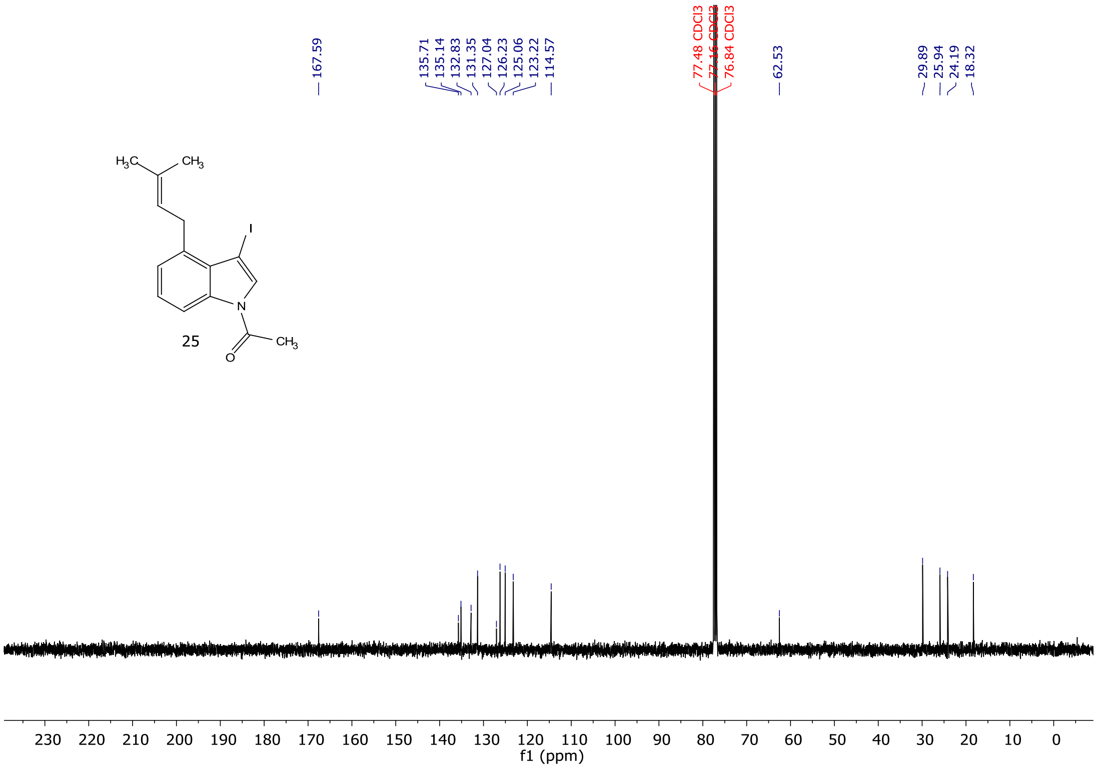

Figure S17: ^{1}H NMR (400 MHz, $CDCl_3$) spectrum (top) and $^{13}C\{^{1}H\}$ NMR (101 MHz, $CDCl_3$) spectrum (bottom) for **25**.

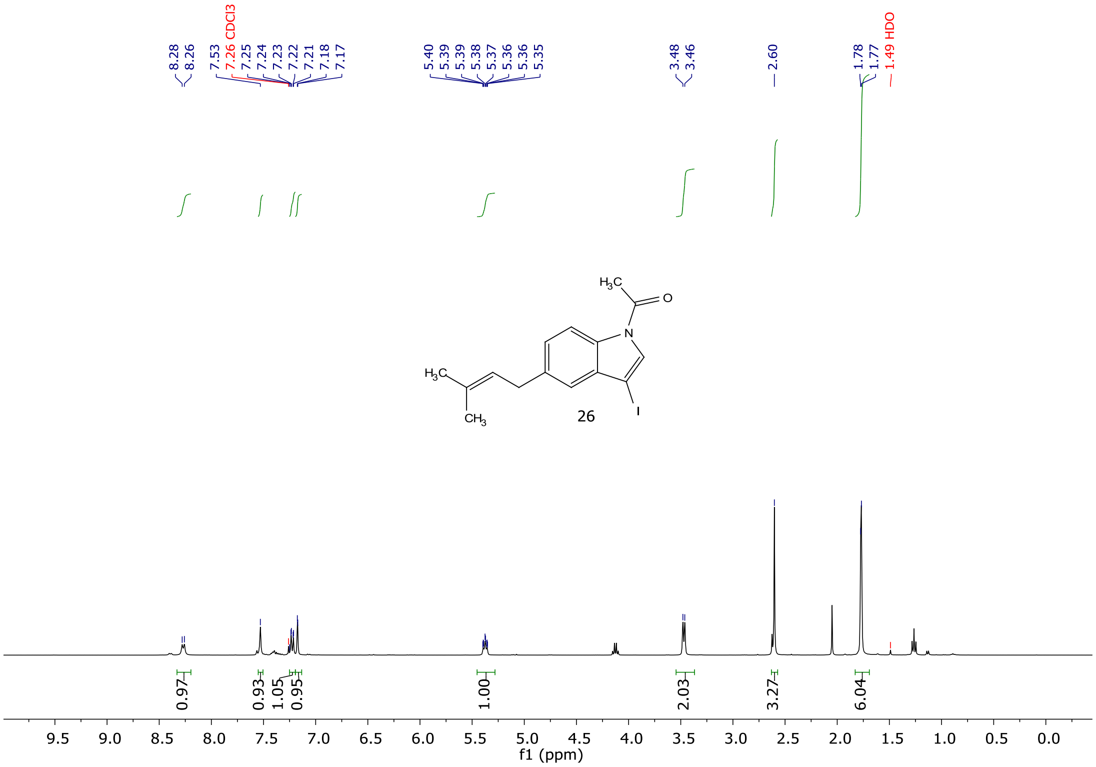

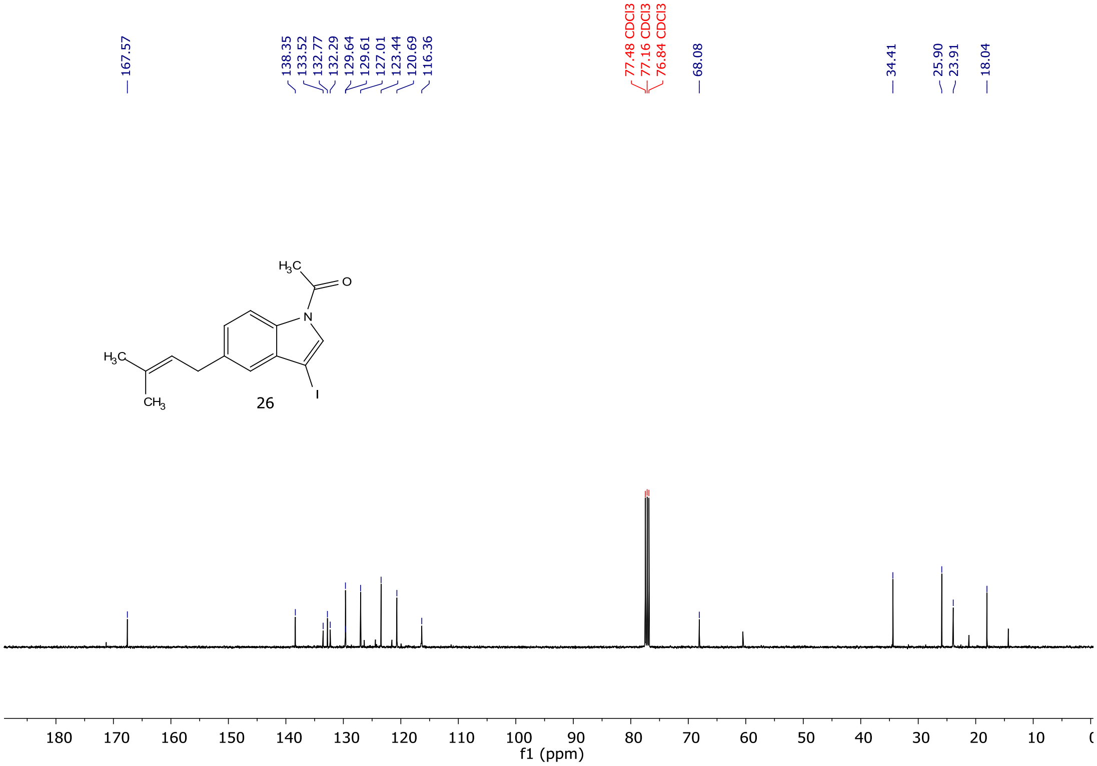

Figure S18: ^{1}H NMR (400 MHz, $CDCl_3$) spectrum (top) and ^{13}C {^{1}H} NMR (101 MHz, $CDCl_3$) spectrum (bottom) for **26**.

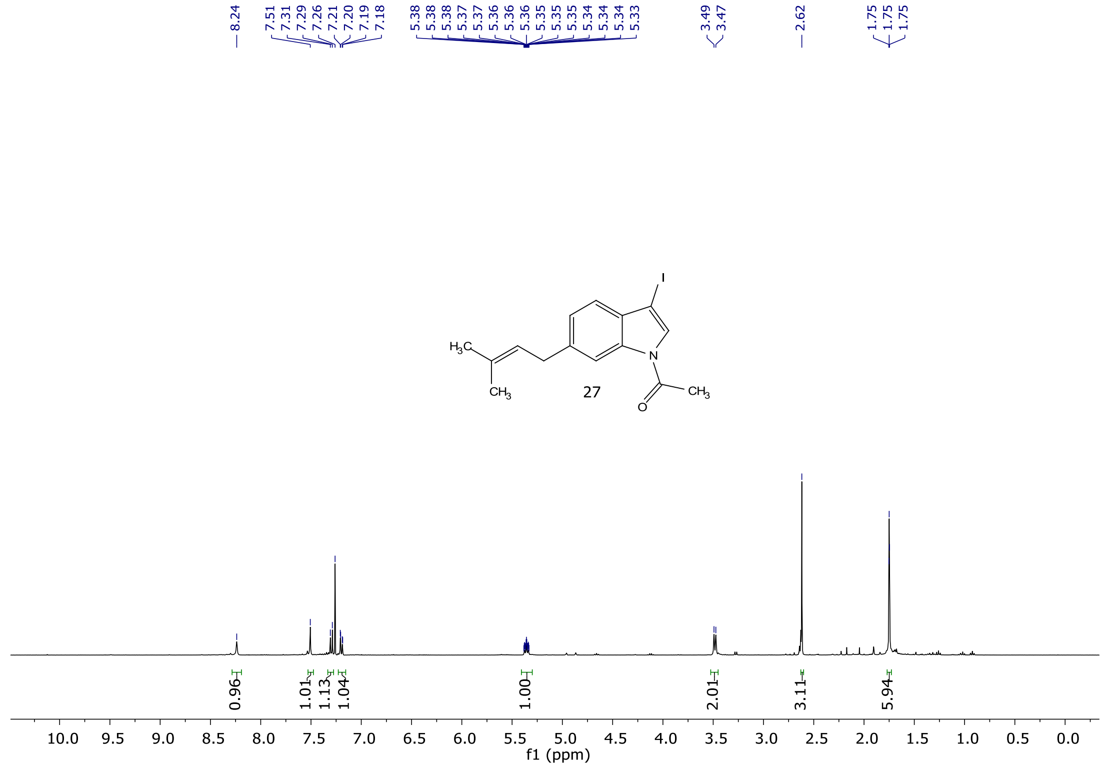

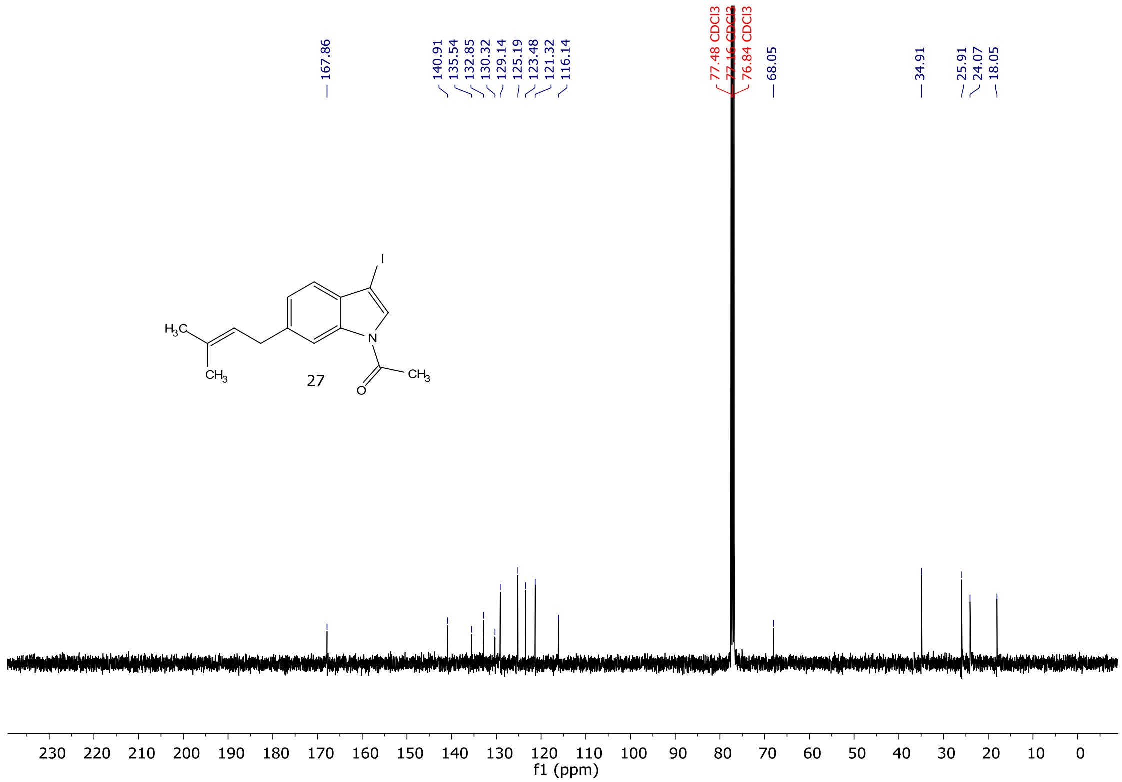

Figure S19: ^{1}H NMR (400 MHz, $CDCl_3$) spectrum (top) and ^{13}C {^{1}H} NMR (101 MHz, $CDCl_3$) spectrum (bottom) for **27**.

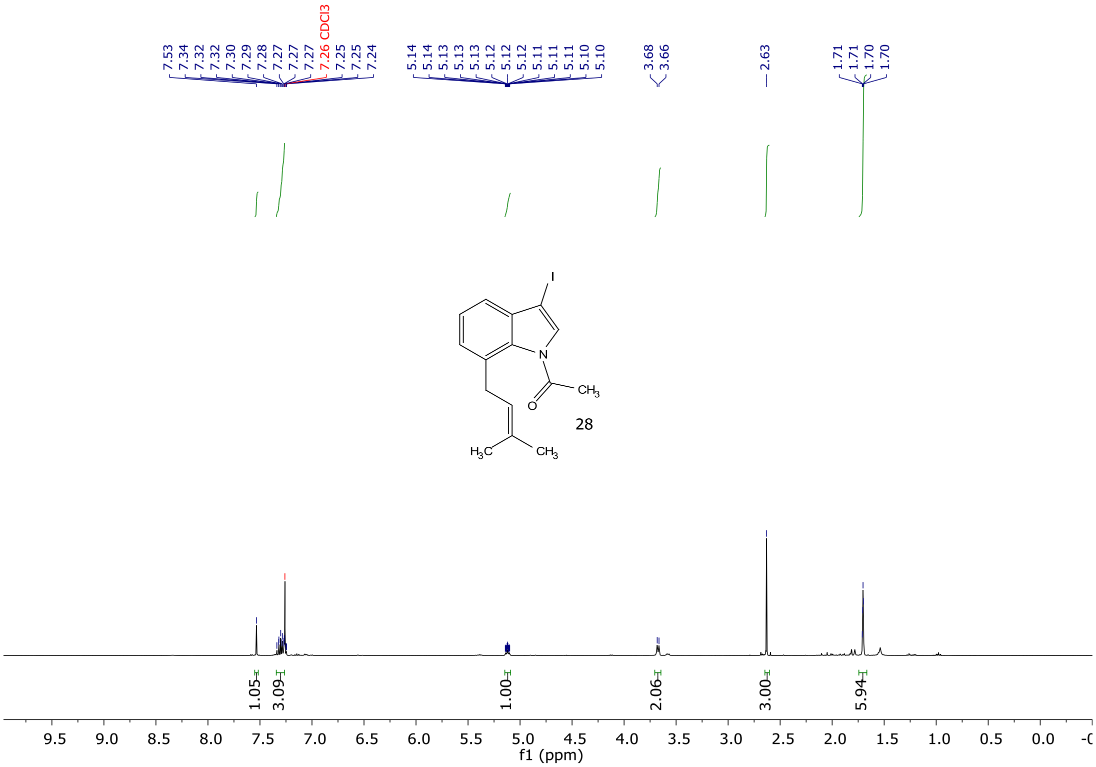

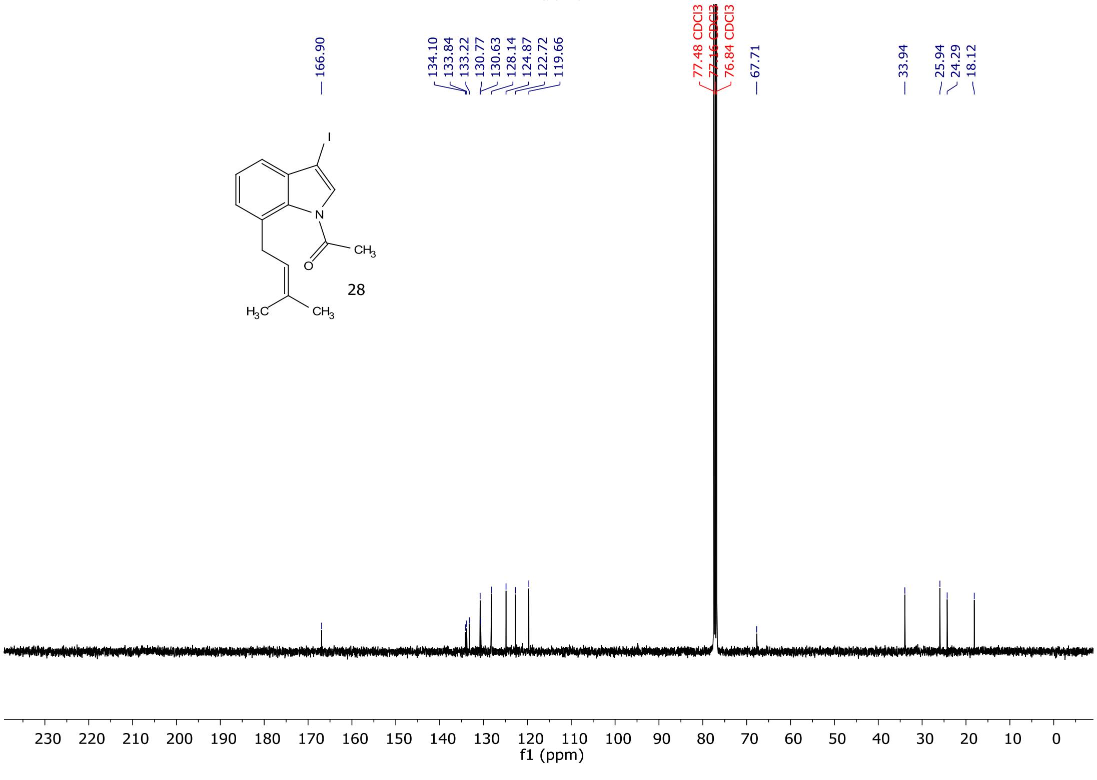

Figure S20:^{1}H NMR (400 MHz, $CDCl_3$) spectrum (top) and ^{13}C {^{1}H} NMR (101 MHz, $CDCl_3$) spectrum (bottom) for **28.**

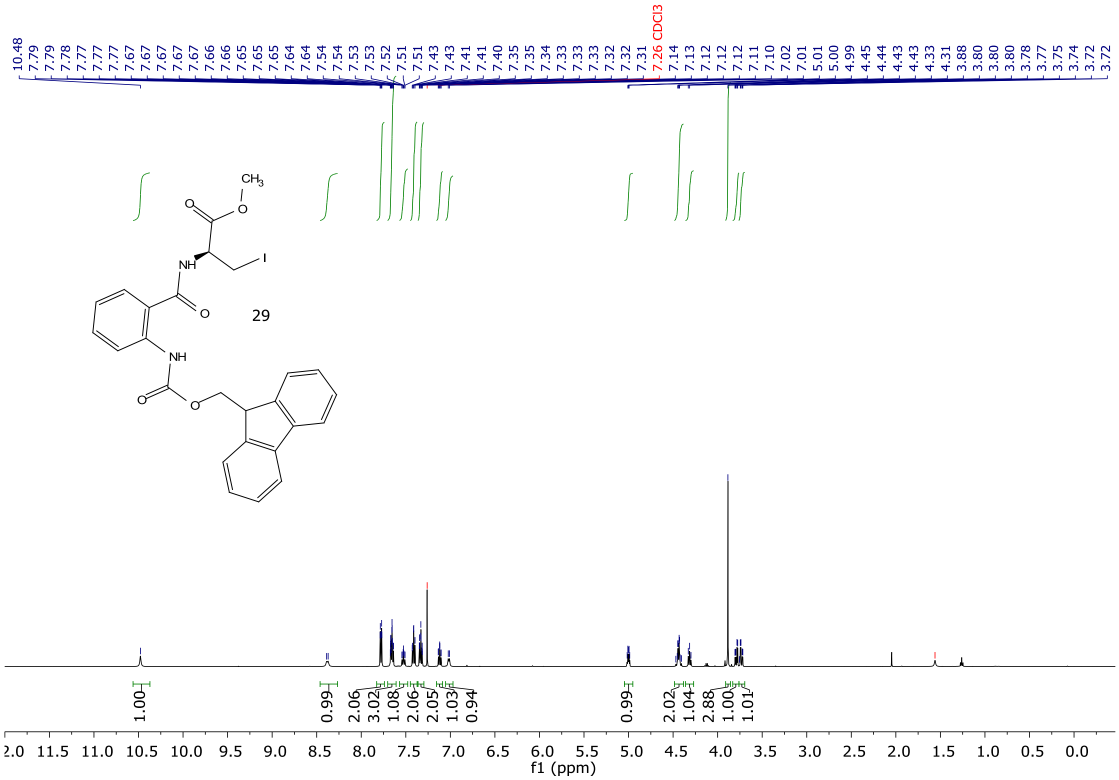

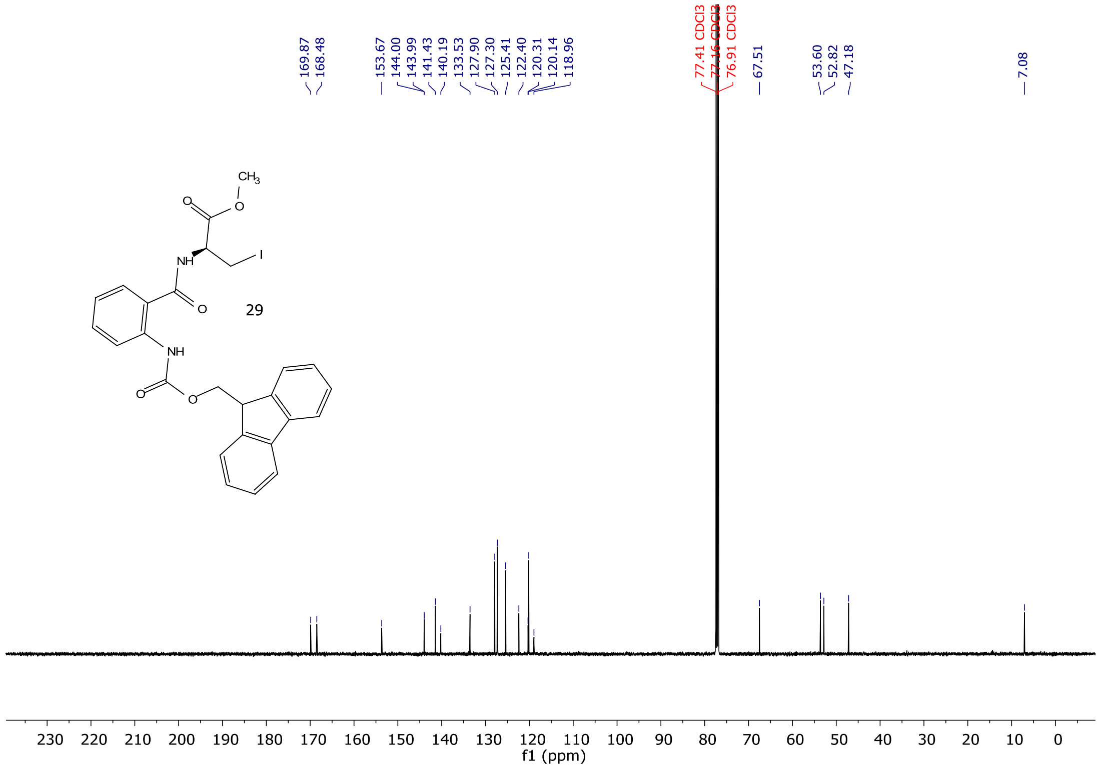

Figure S21: ^{1}H NMR (500 MHz, $CDCl_3$) spectrum (top) and ^{13}C {^{1}H} NMR (126 MHz, $CDCl_3$) spectrum (bottom) for **29**.

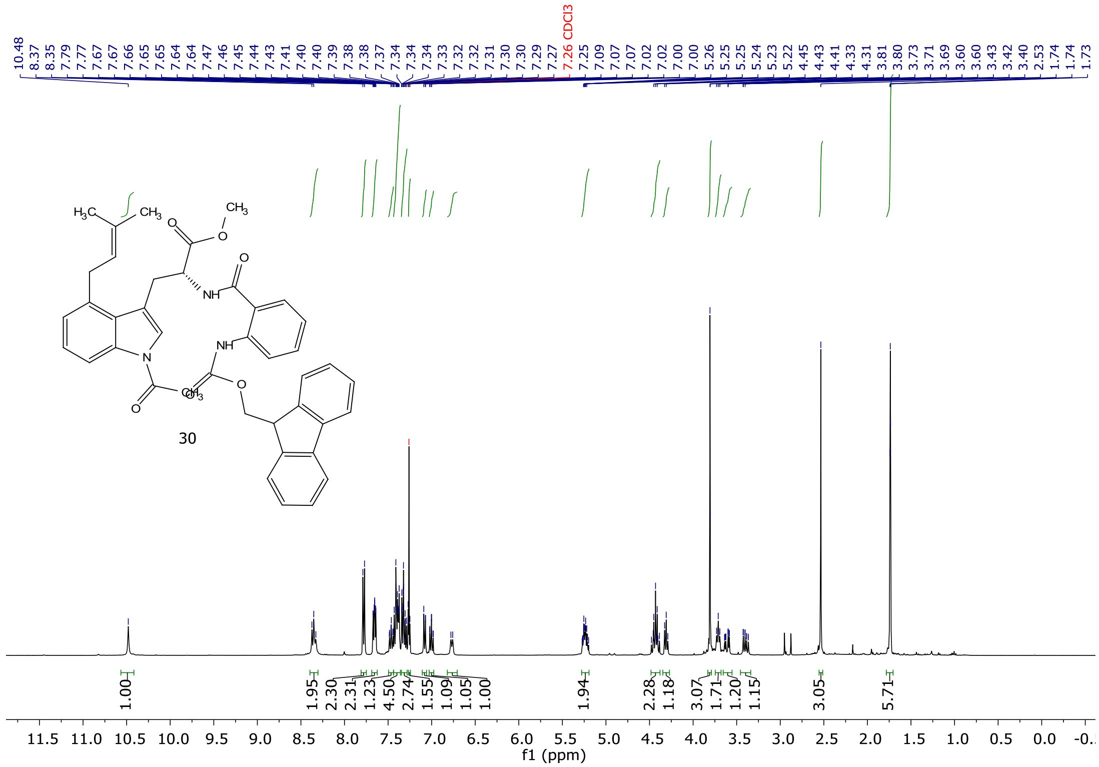

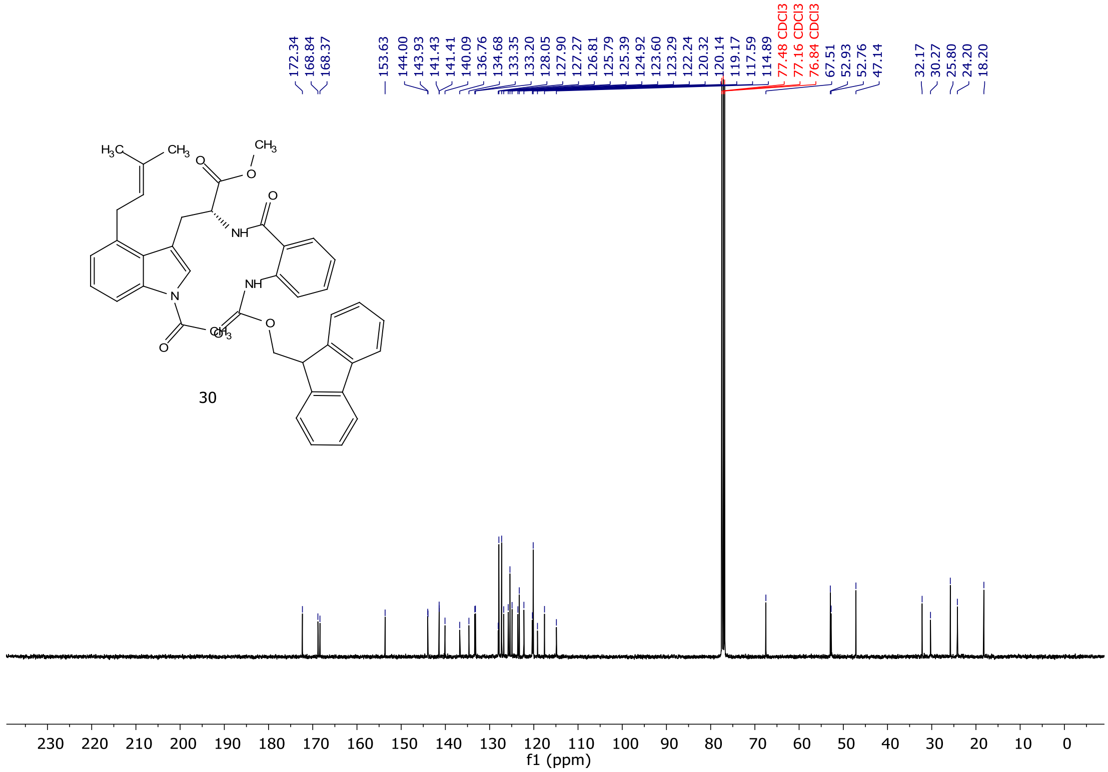

Figure S22: ^{1}H NMR (400 MHz, $CDCl_3$) spectrum (top) and ^{13}C {^{1}H} NMR (101 MHz, $CDCl_3$) spectrum (bottom) for **30**.

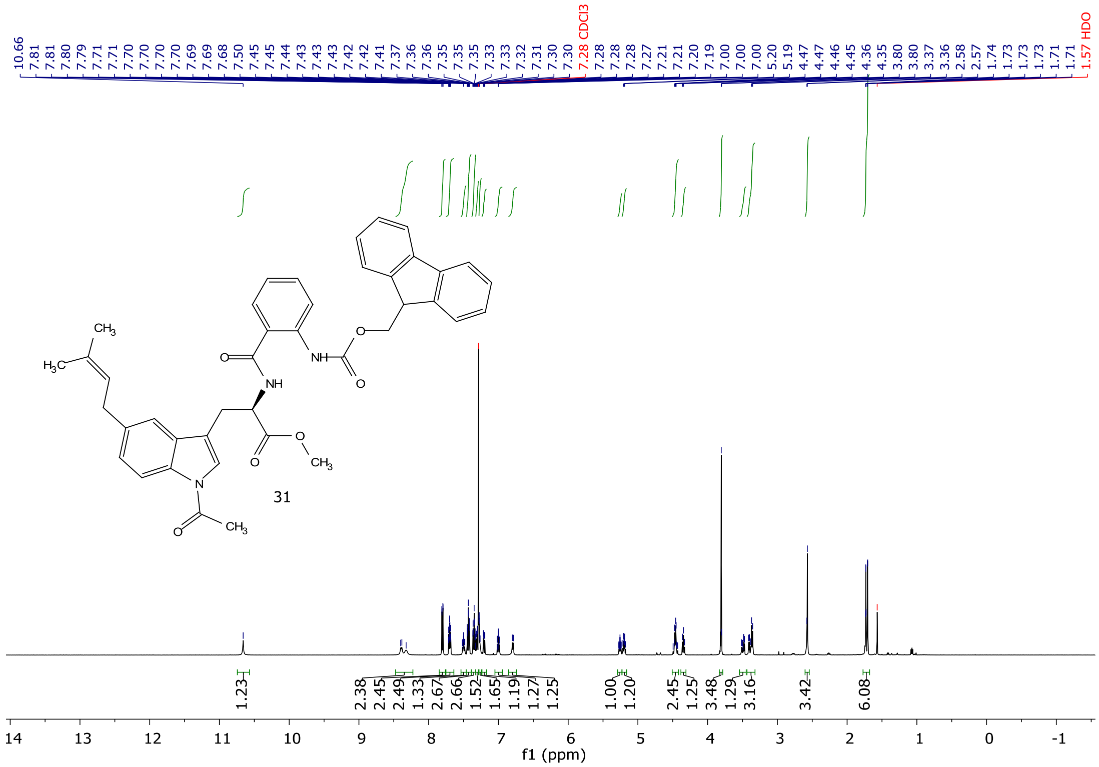

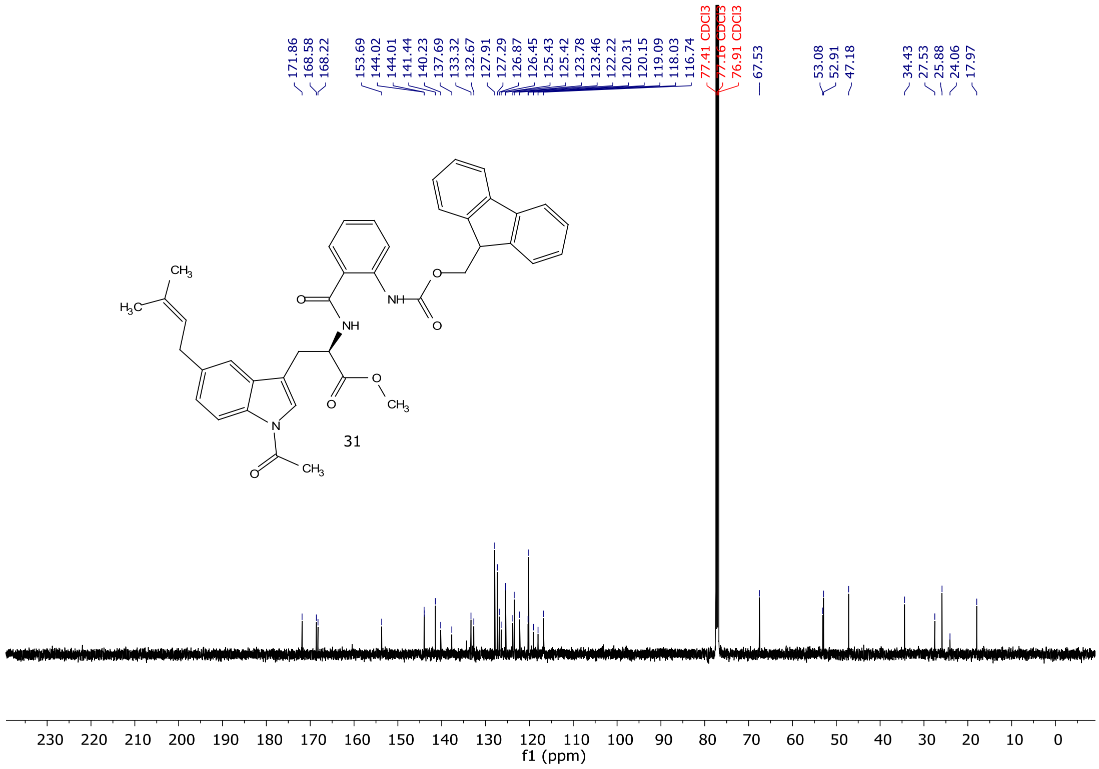

Figure S23: ^{1}H NMR (500 MHz, $CDCl_3$) spectrum (top) and ^{13}C {^{1}H} NMR (126 MHz, $CDCl_3$) spectrum (bottom) for **31**.

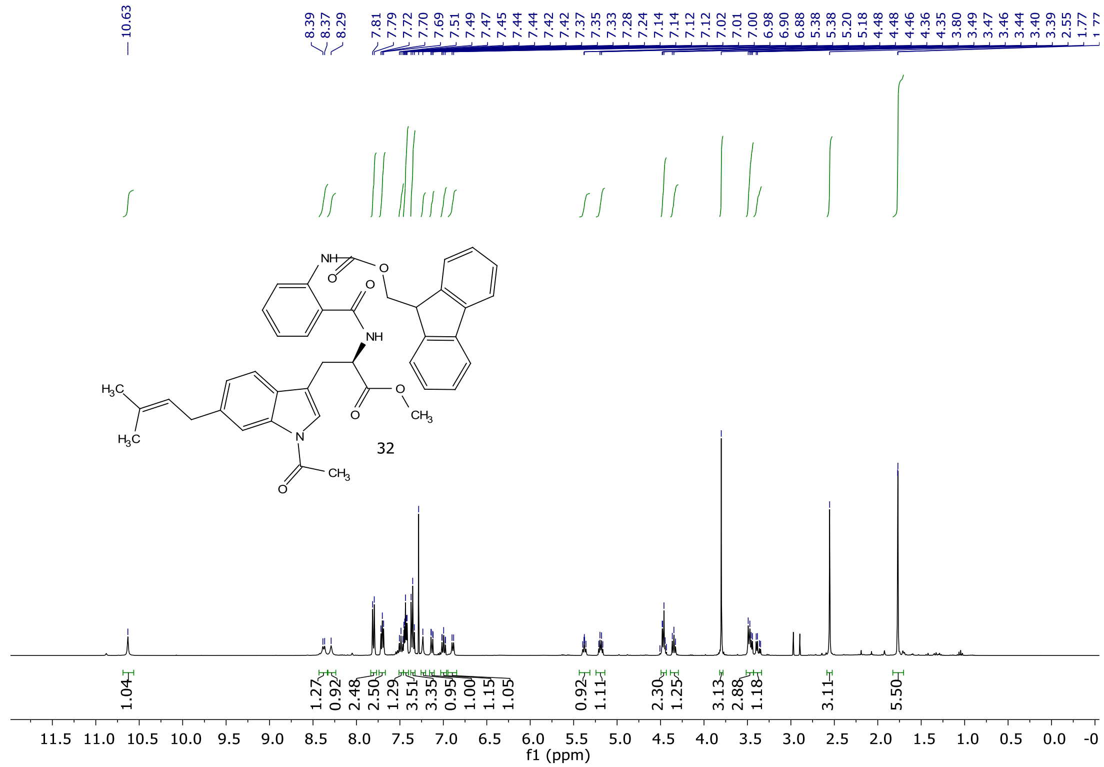

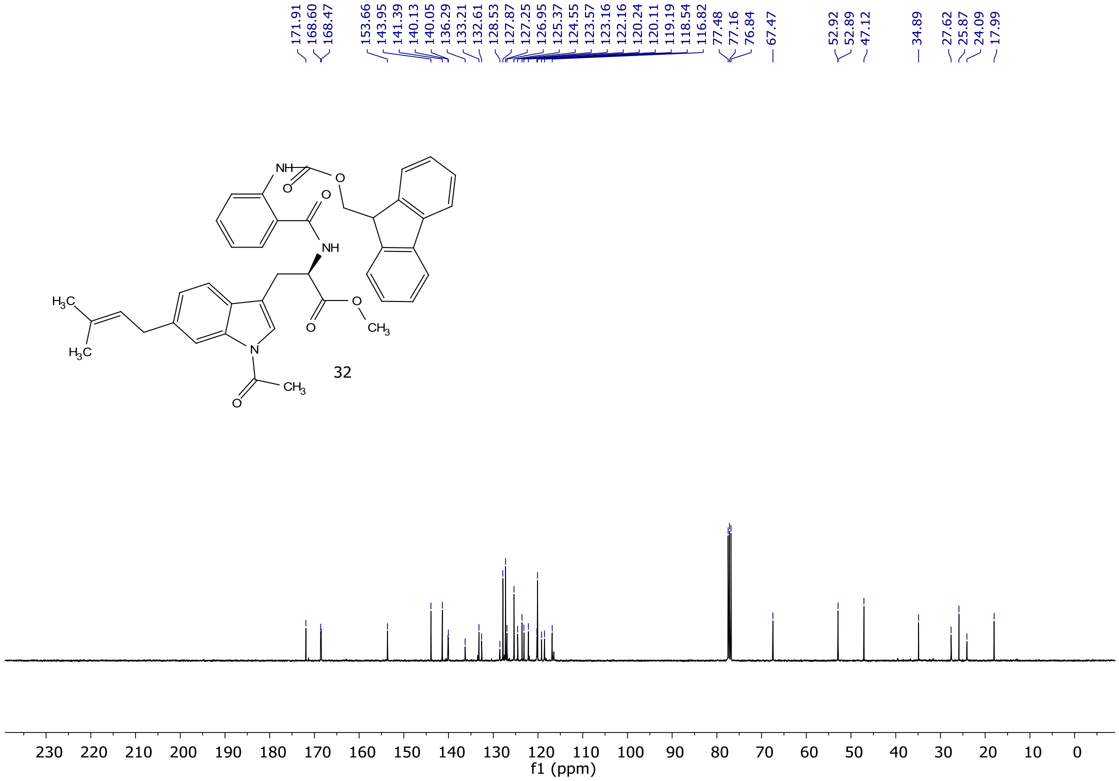

Figure S24:^{1}H NMR (400 MHz, $CDCl_3$) spectrum (top) and ^{13}C {^{1}H} NMR (101 MHz, $CDCl_3$) spectrum (bottom) for **32**.

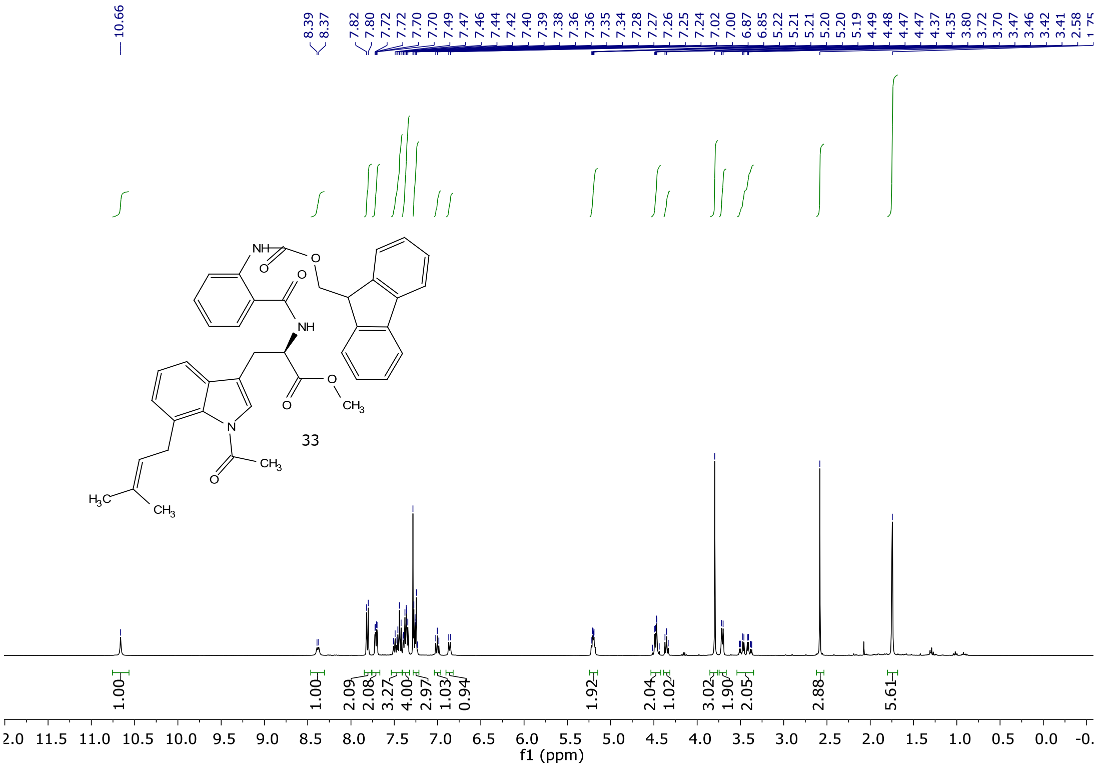

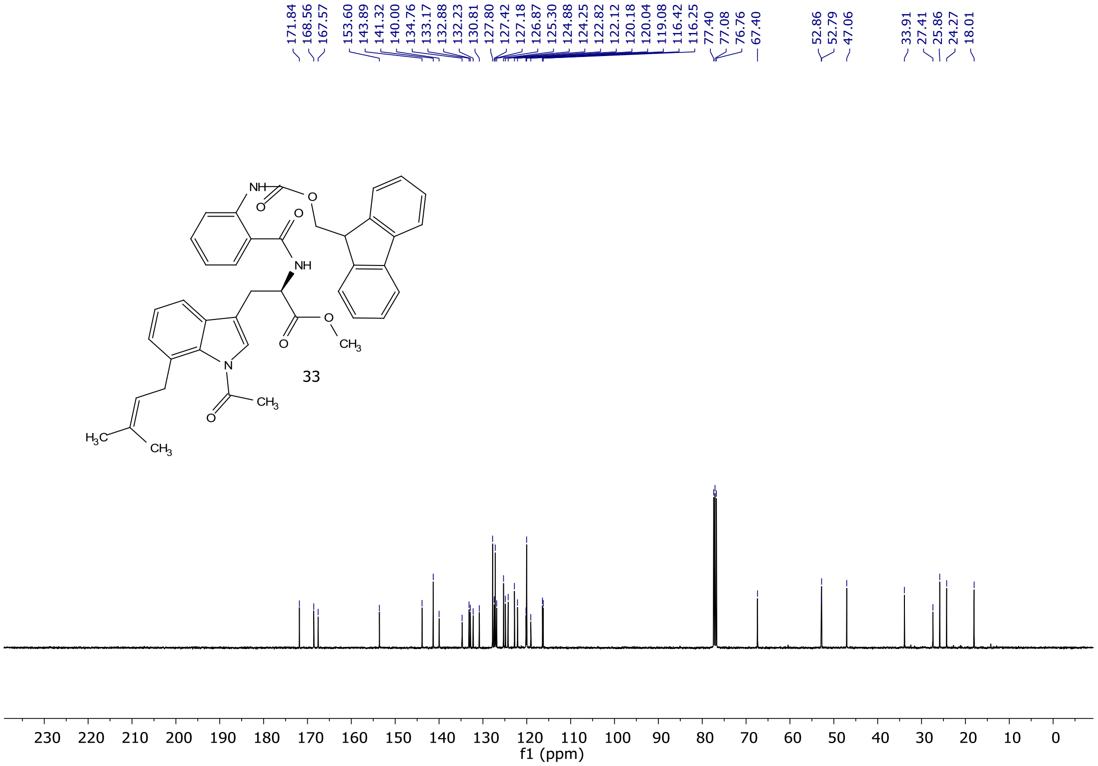

Figure S25: ^{1}H NMR (400 MHz, $CDCl_3$) spectrum (top) and ^{13}C {^{1}H} NMR (101 MHz, $CDCl_3$) spectrum (bottom) for **33**.

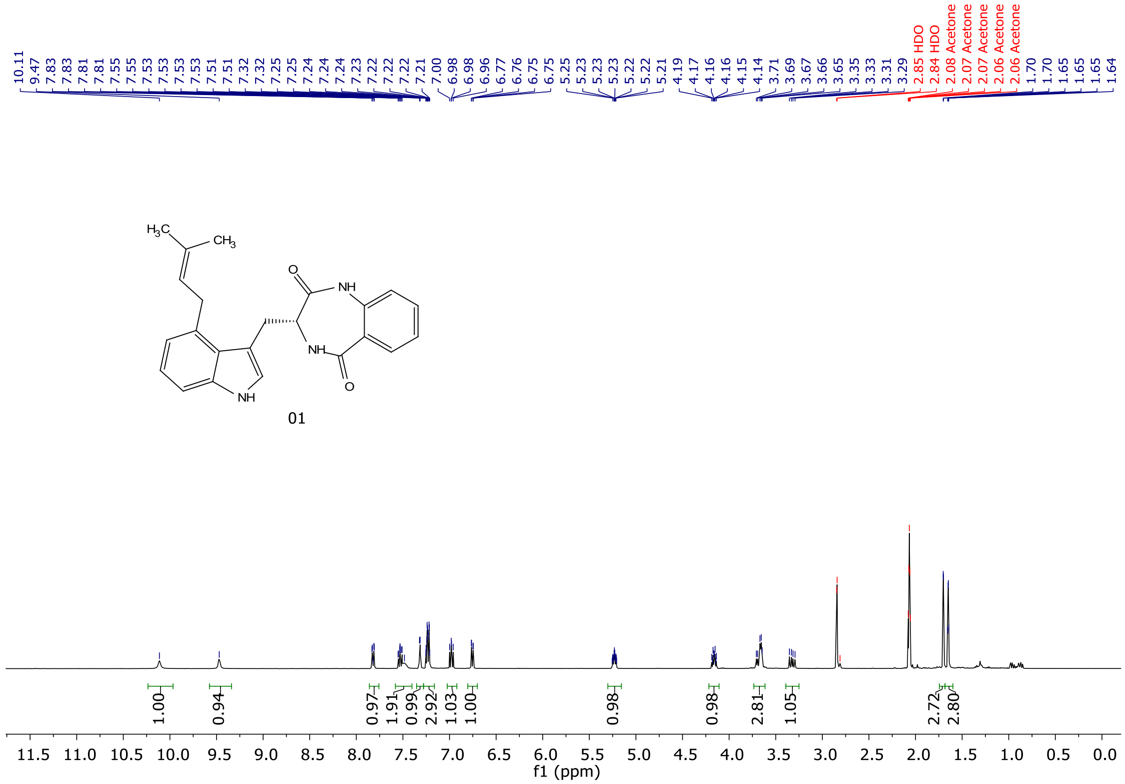

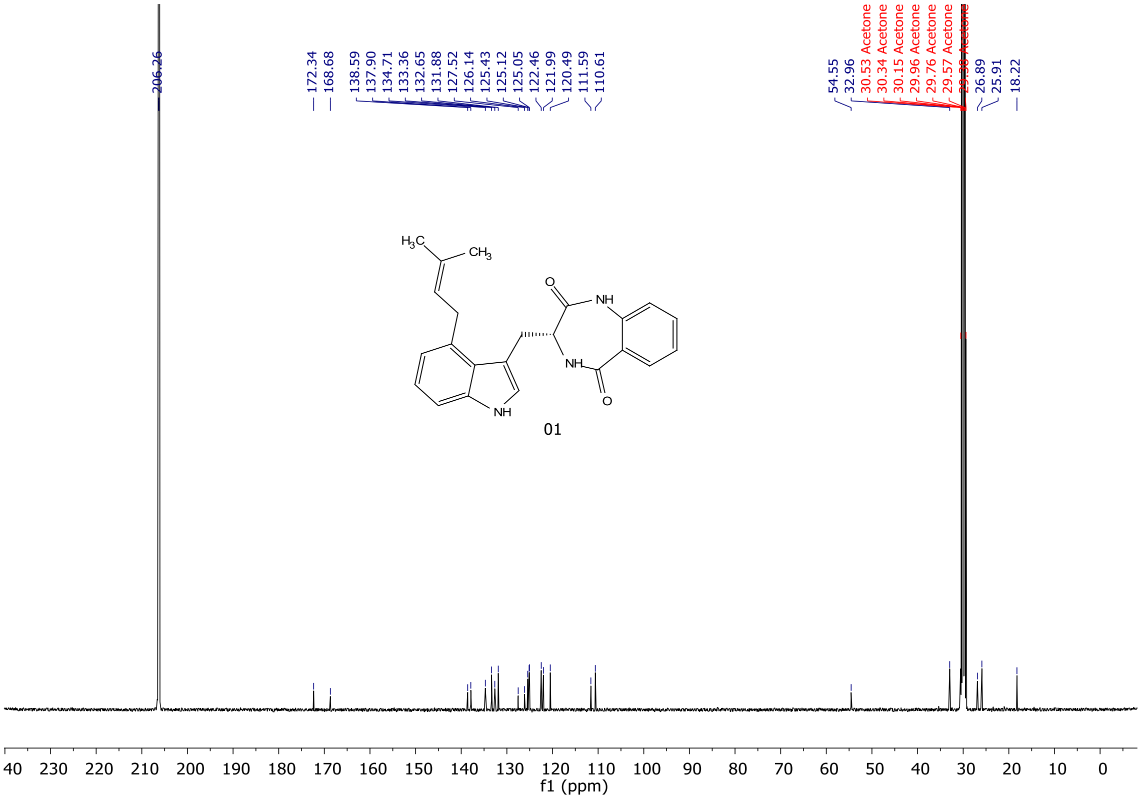

Figure S26: ^{1}H NMR (400 MHz, acetone-d_6) spectrum (top) and ^{13}C {^{1}H} NMR (101 MHz, acetone) spectrum (bottom) for **1**.

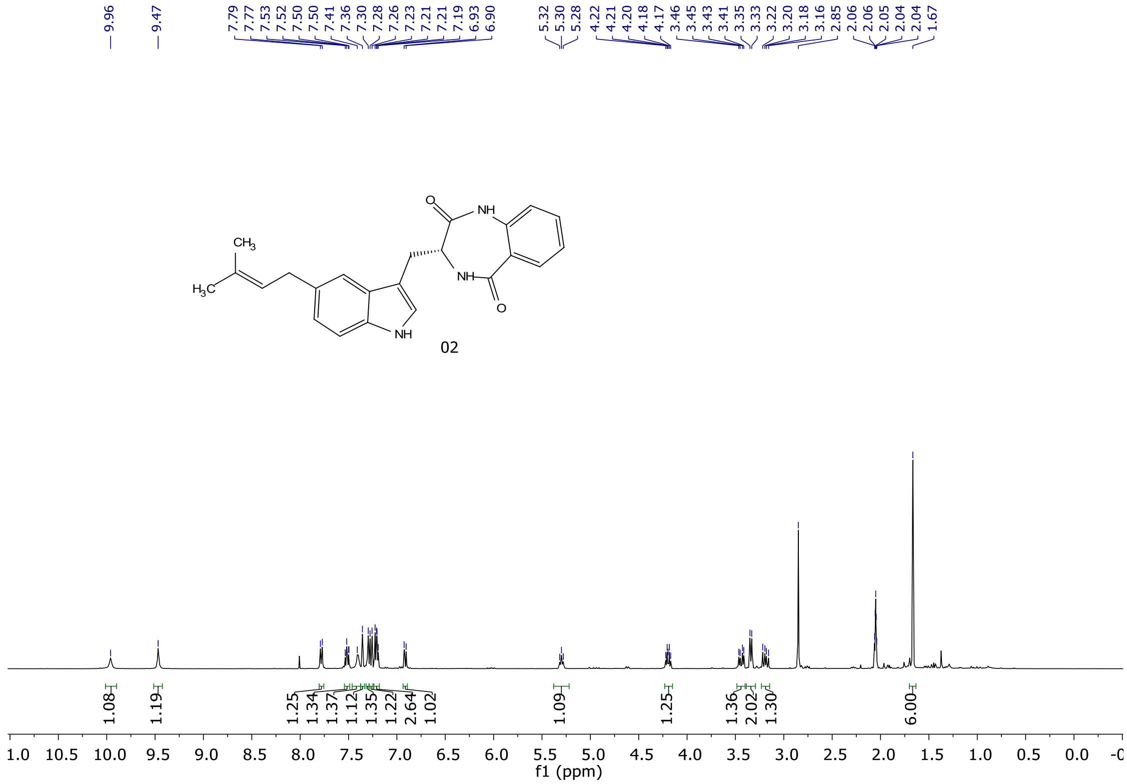

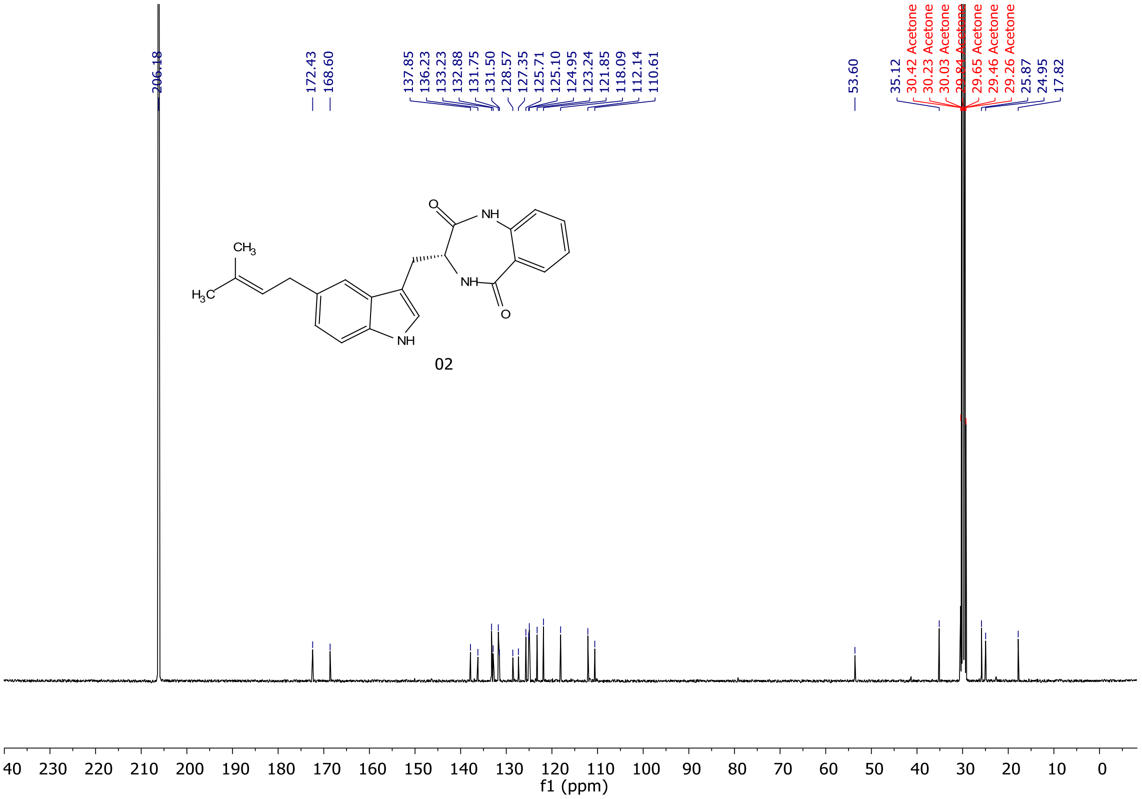

Figure S27:^{1}H NMR (400 MHz, acetone-d_6) spectrum (top) and ^{13}C {^{1}H} NMR (101 MHz, acetone) spectrum (bottom) for **2**.

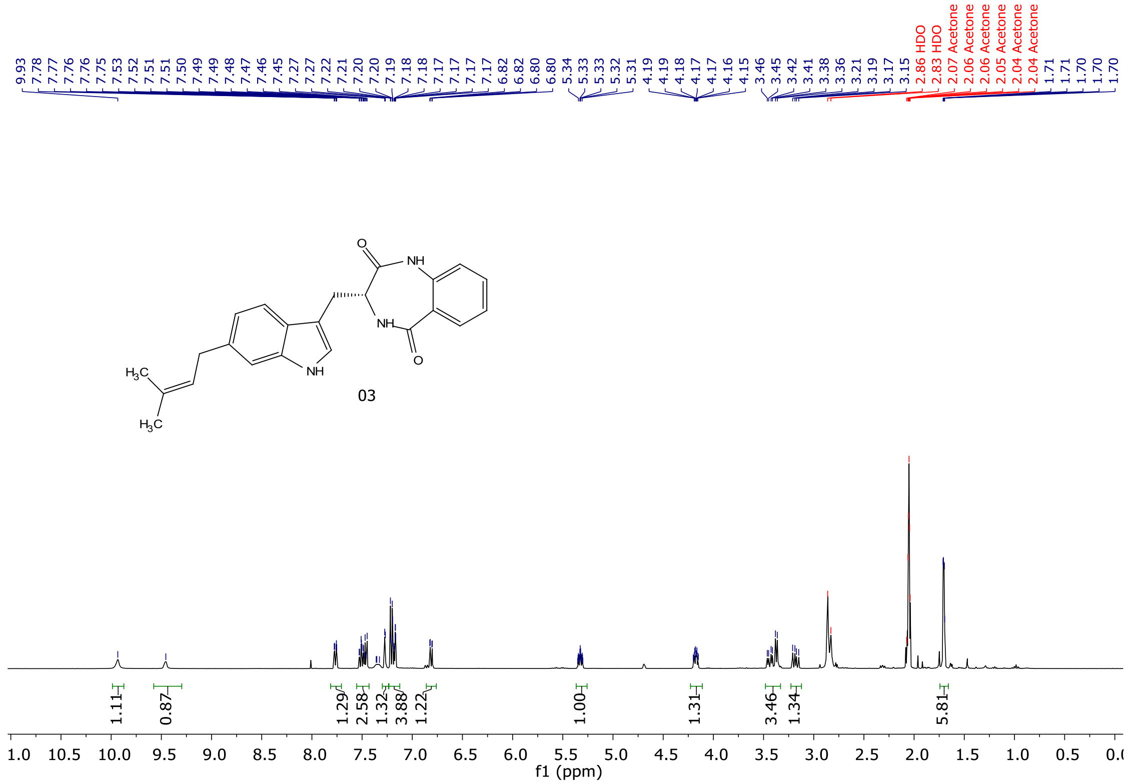

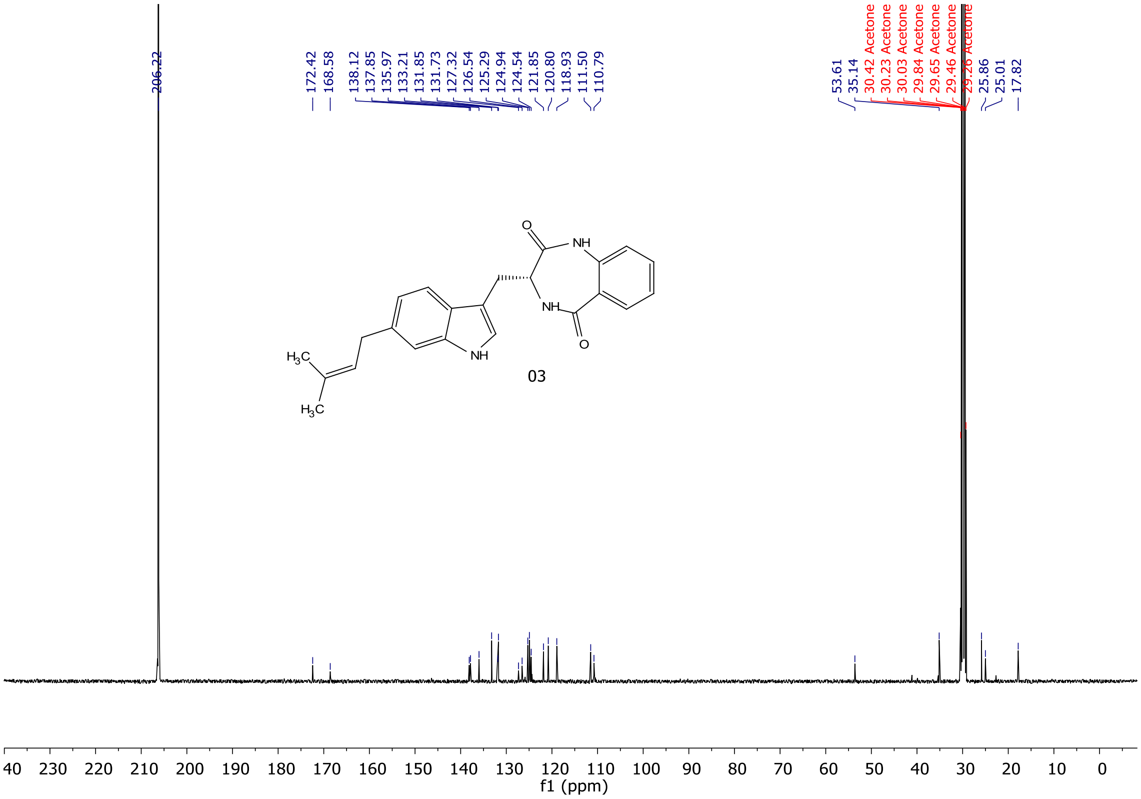

Figure S28:^{1}H NMR (400 MHz, acetone-d_6) spectrum (top) and ^{13}C {^{1}H} NMR (101 MHz, acetone) spectrum (bottom) for **3**.

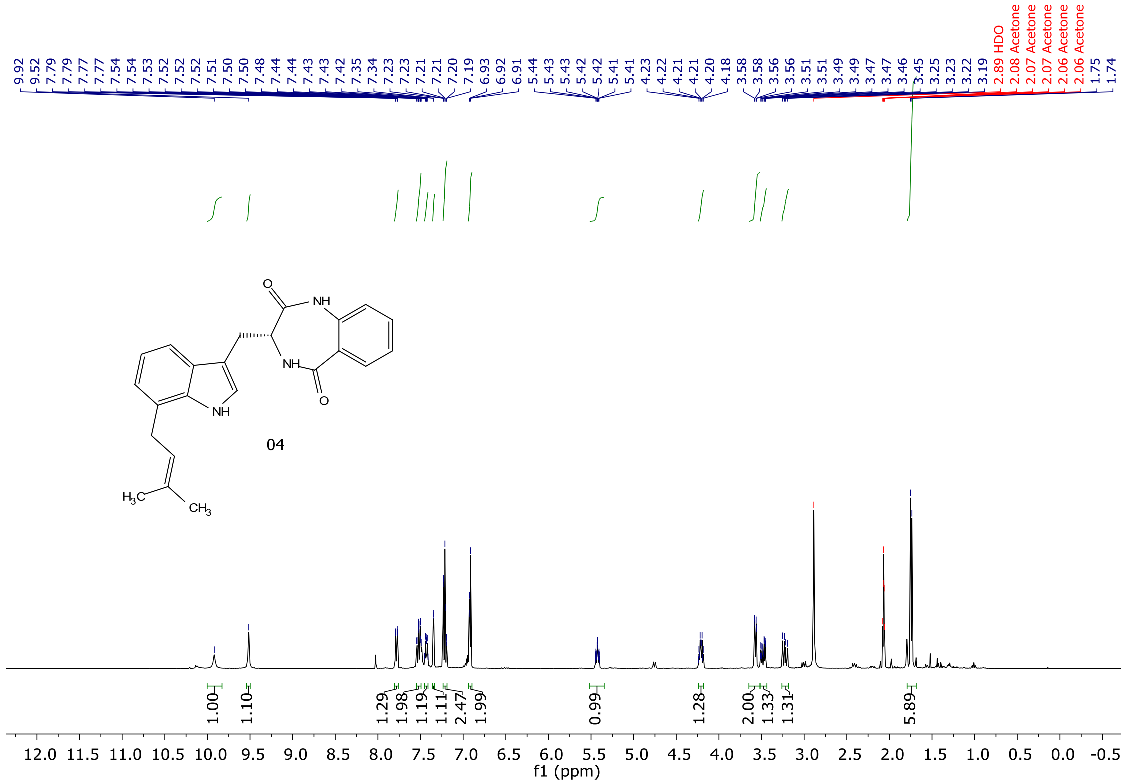

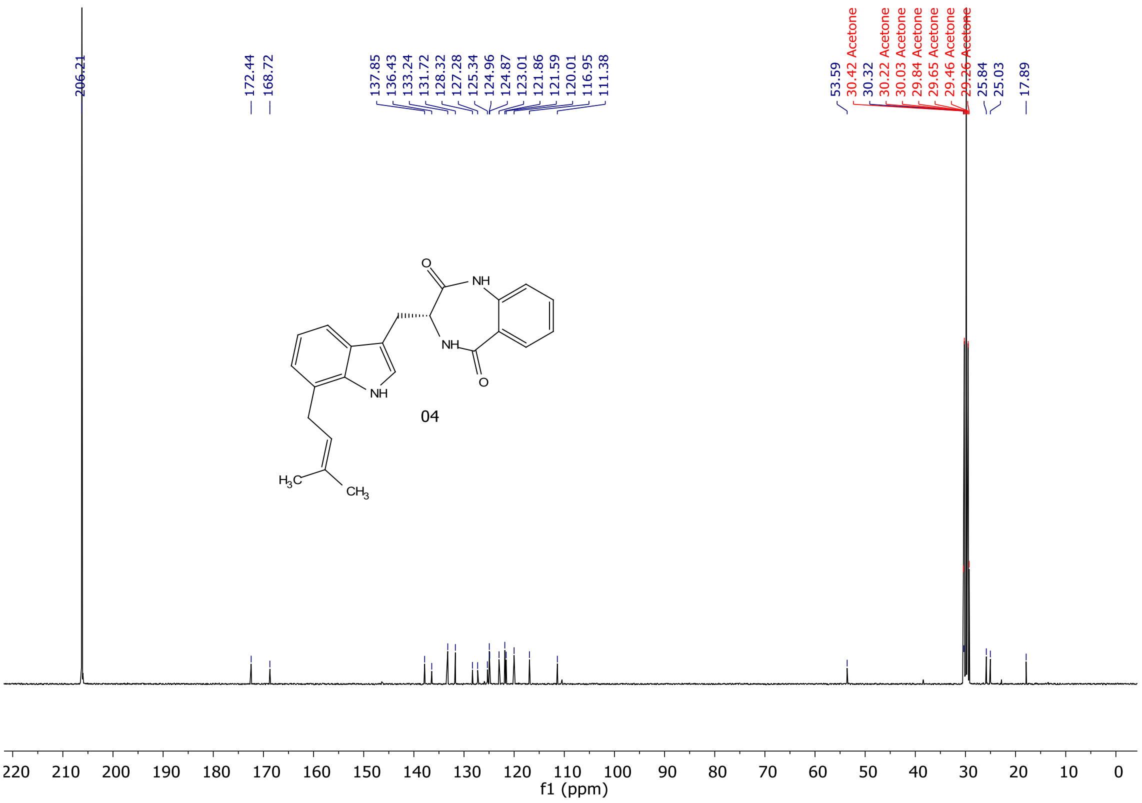

Figure S29: ^{1}H NMR (400 MHz, acetone-d_6) spectrum (top) and ^{13}C {^{1}H} NMR (101 MHz, acetone) spectrum (bottom) for **4**.

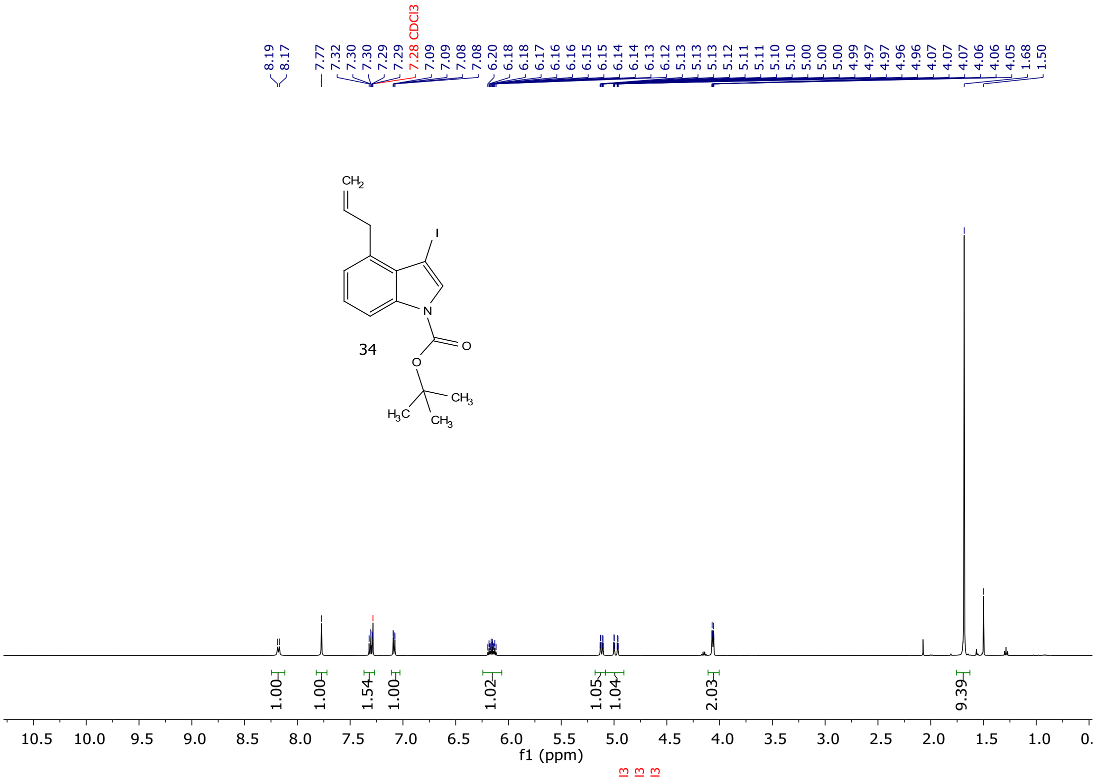

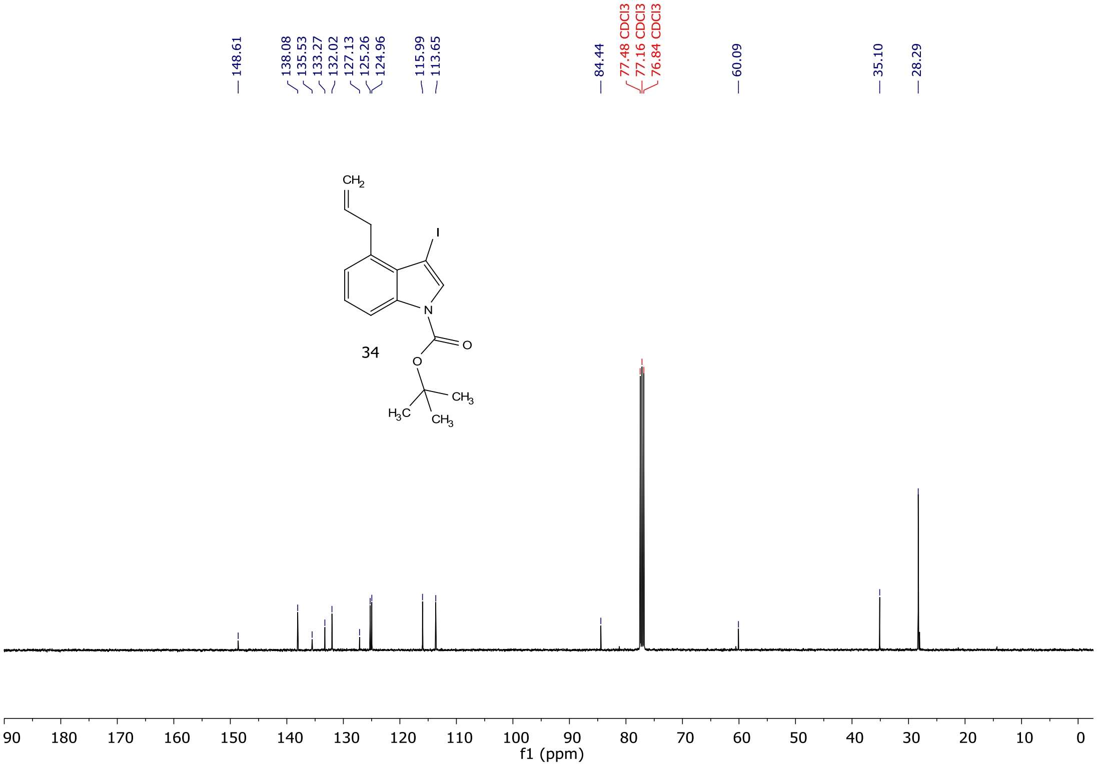

Figure S30:^{1}H NMR (400 MHz, Chloroform-*d*) spectrum (top) and ^{13}C {^{1}H} NMR (101 MHz, Chloroform) spectrum (bottom) for **34**.

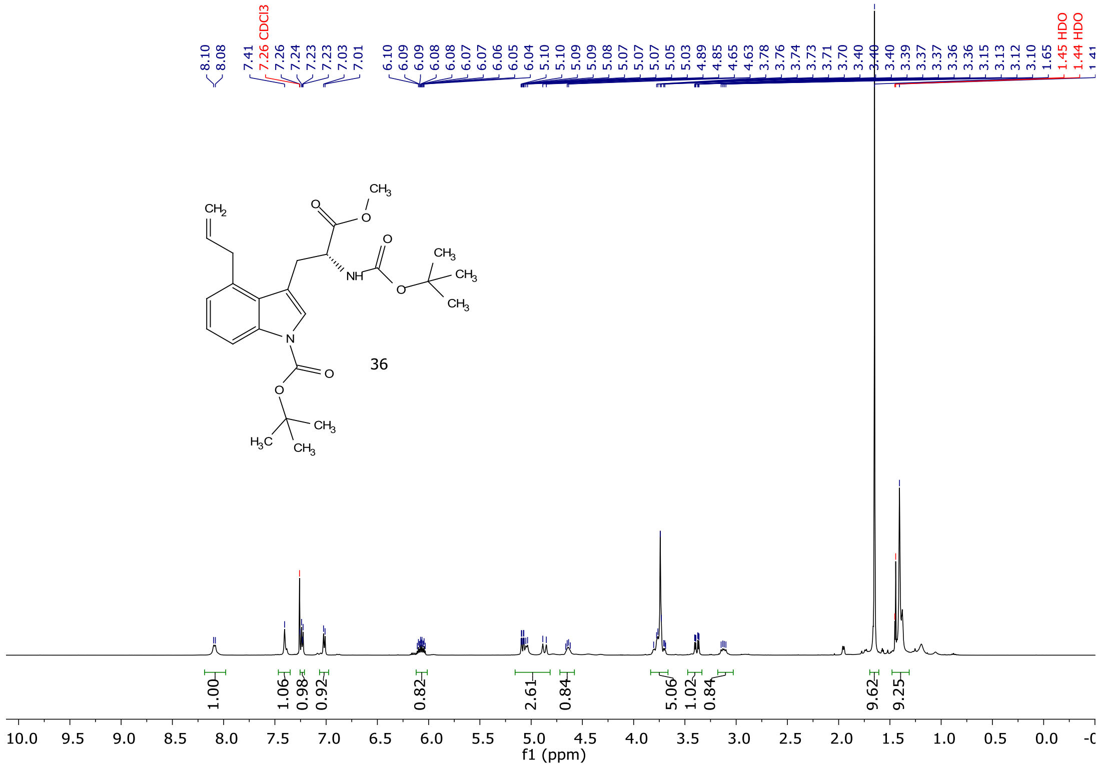

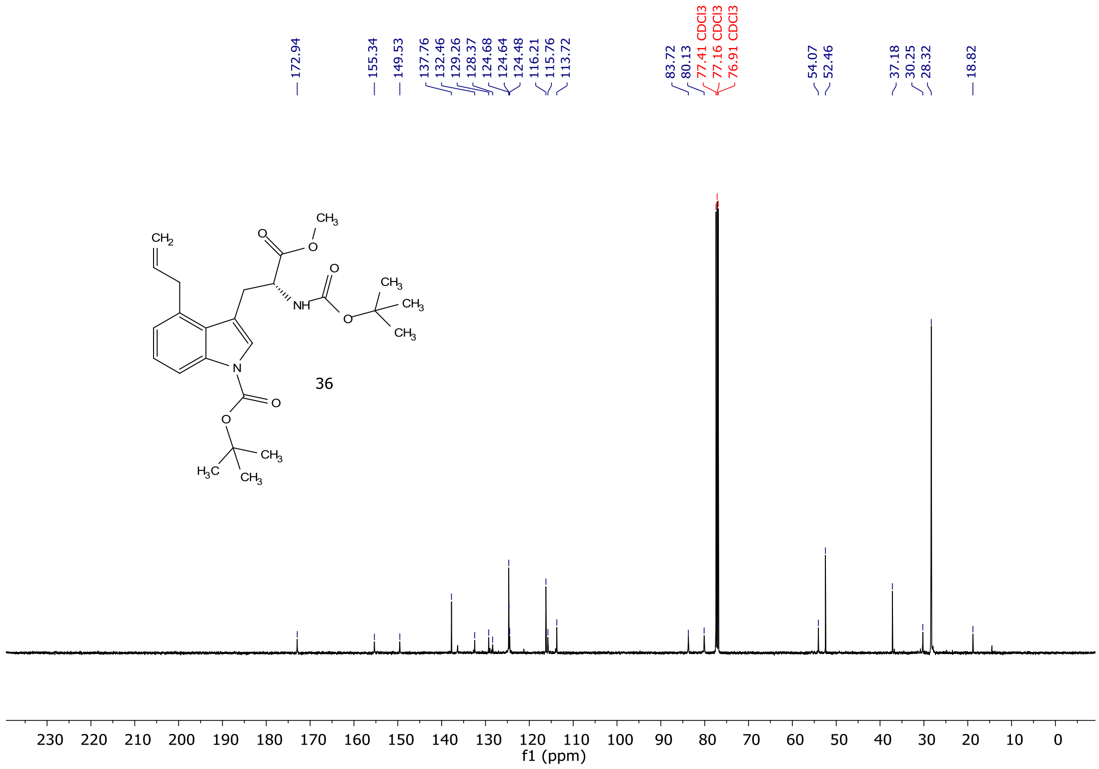

Figure S31: ^{1}H NMR (400 MHz, $CDCl_3$) spectrum (top) and ^{13}C {^{1}H} NMR (101 MHz, $CDCl_3$) spectrum (bottom) for **36**.

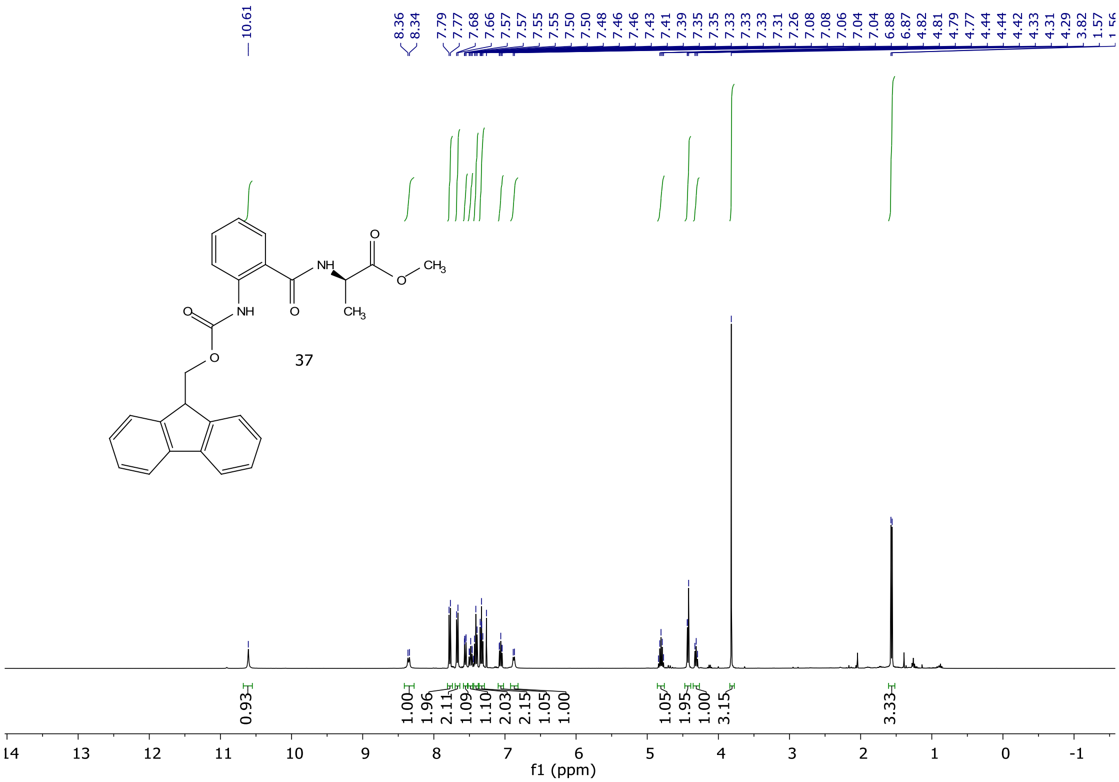

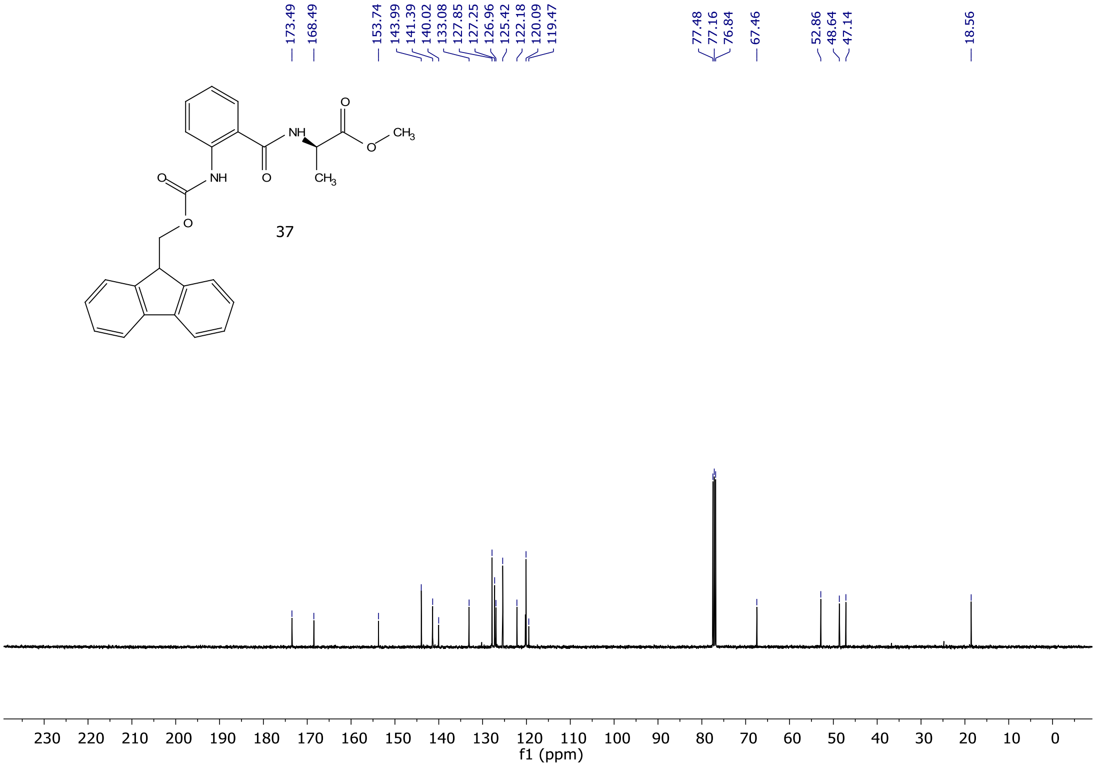

Figure S32:^{1}H NMR (400 MHz, $CDCl_3$) spectrum (top) and ^{13}C {^{1}H} NMR (101 MHz, $CDCl_3$) spectrum (bottom) for **37**.

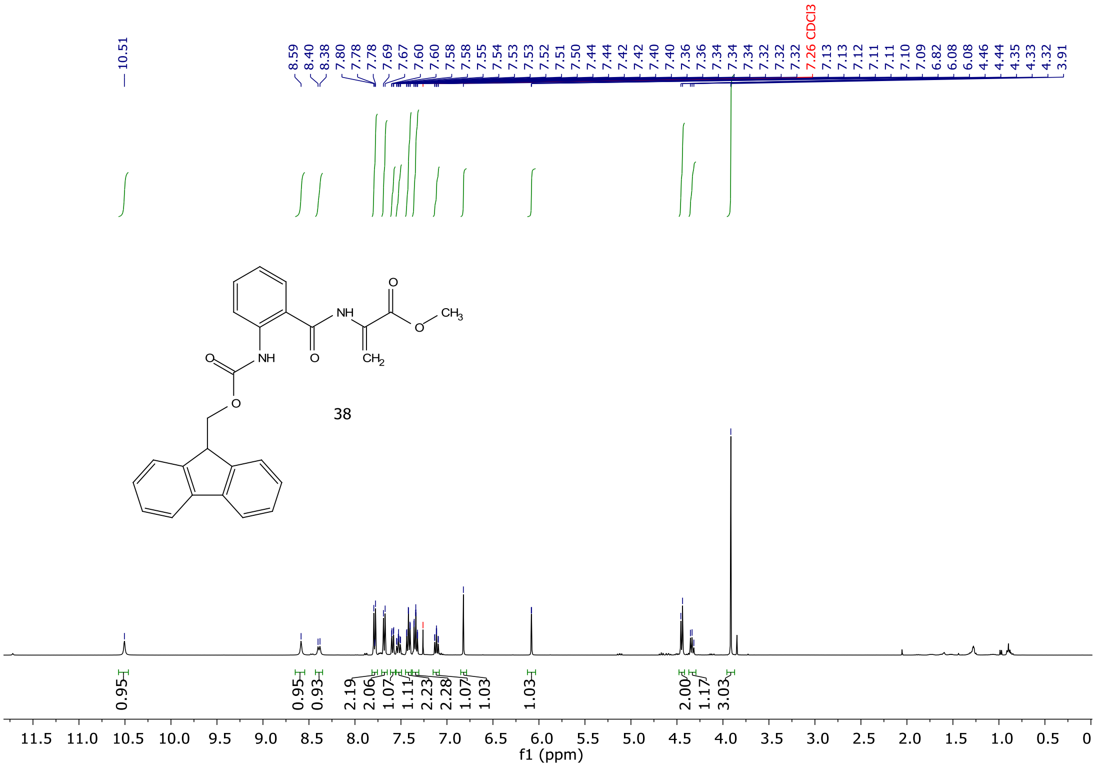

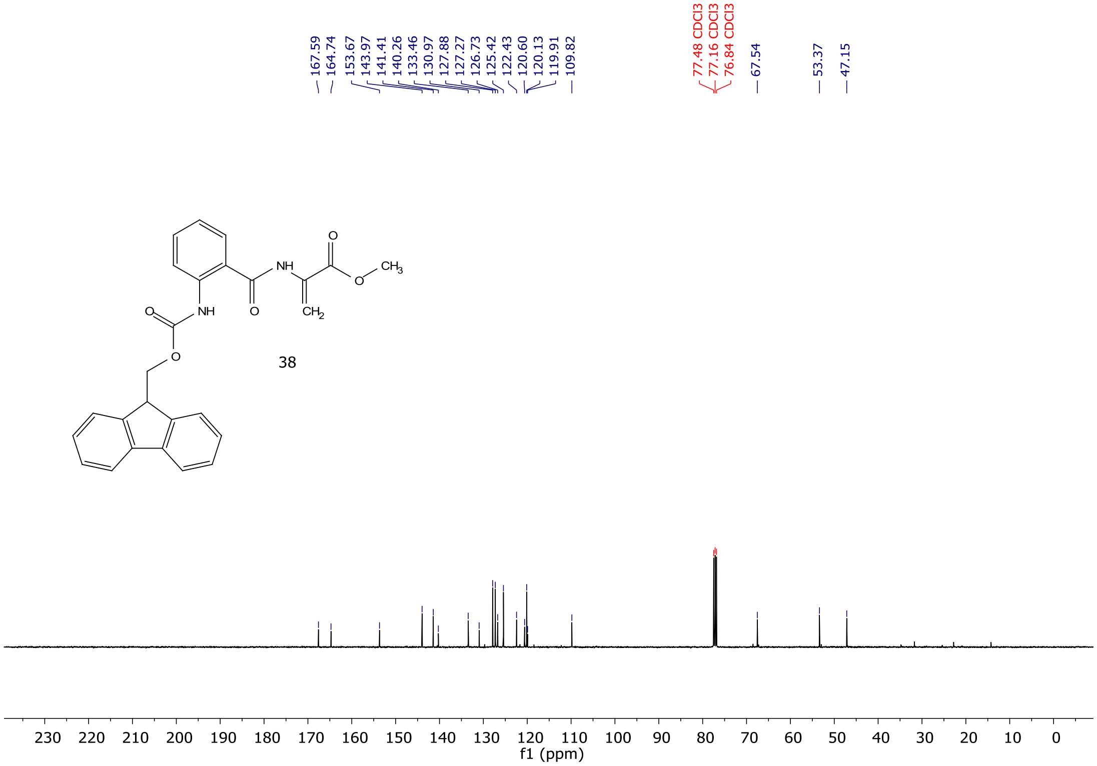

Figure S33: ^{1}H NMR (400 MHz, $CDCl_3$) spectrum (top) and ^{13}C {^{1}H} NMR (101 MHz, $CDCl_3$) spectrum (bottom) for **38**.

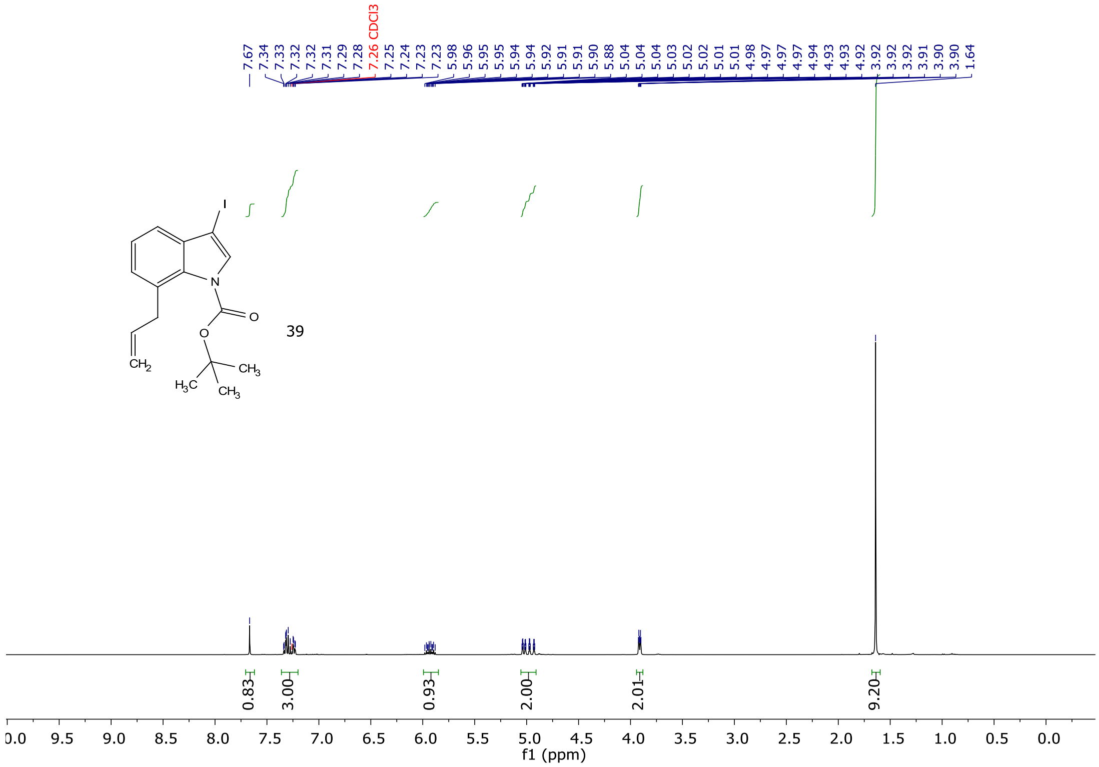

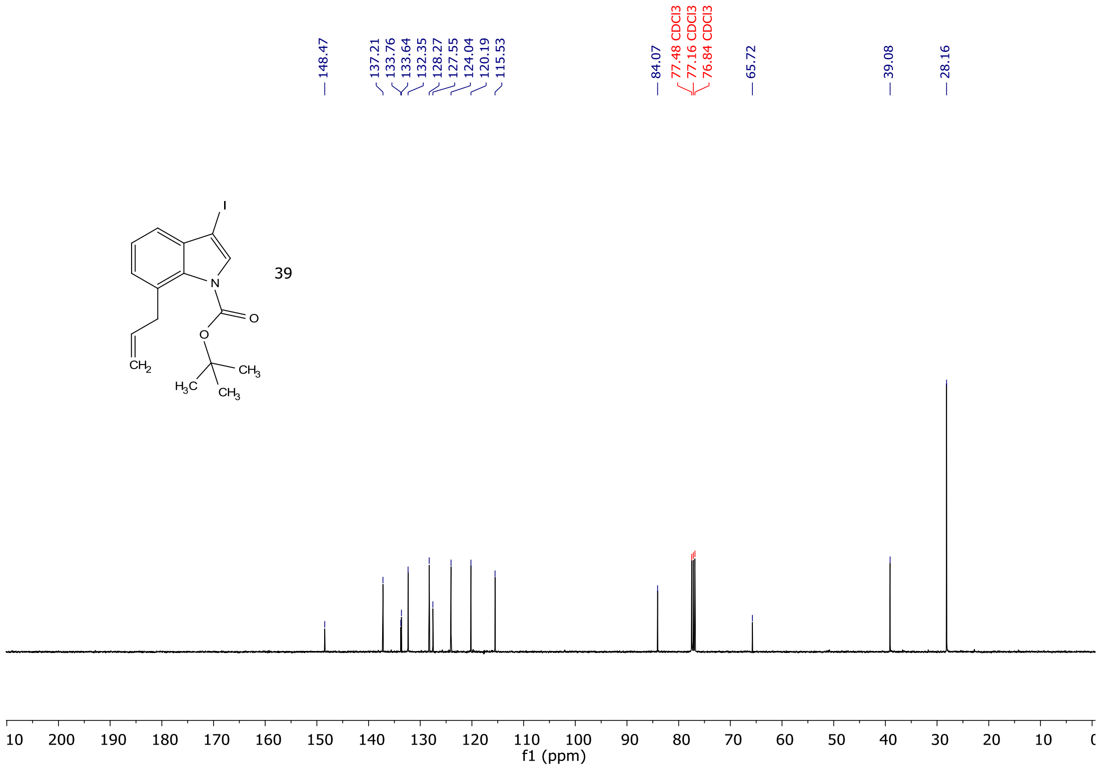

Figure S34: ^{1}H NMR (400 MHz, $CDCl_3$) spectrum (top) and ^{13}C {^{1}H} NMR (101 MHz, $CDCl_3$) spectrum (bottom) for **39**.

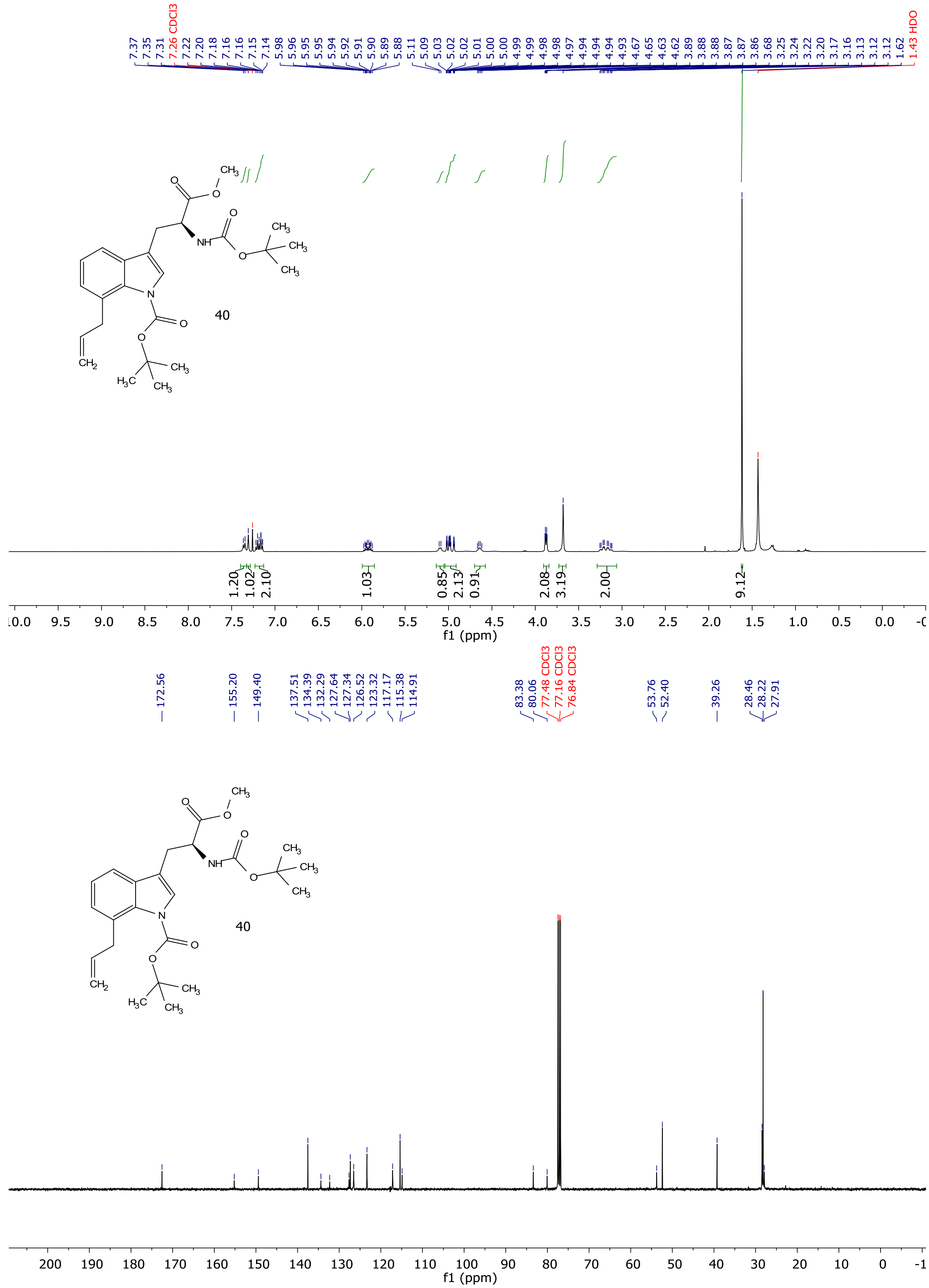

Figure S35: ^{1}H NMR (400 MHz, $CDCl_3$) spectrum (top) and ^{13}C {^{1}H} NMR (101 MHz, $CDCl_3$) spectrum (bottom) for **40**.

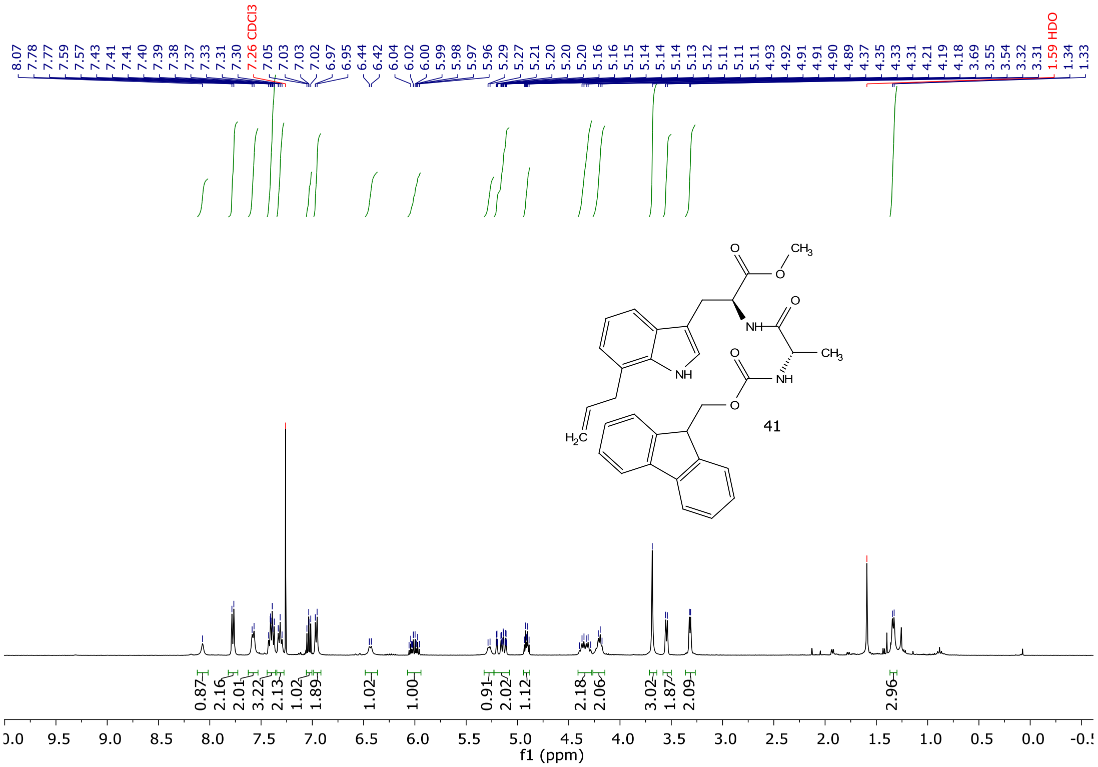

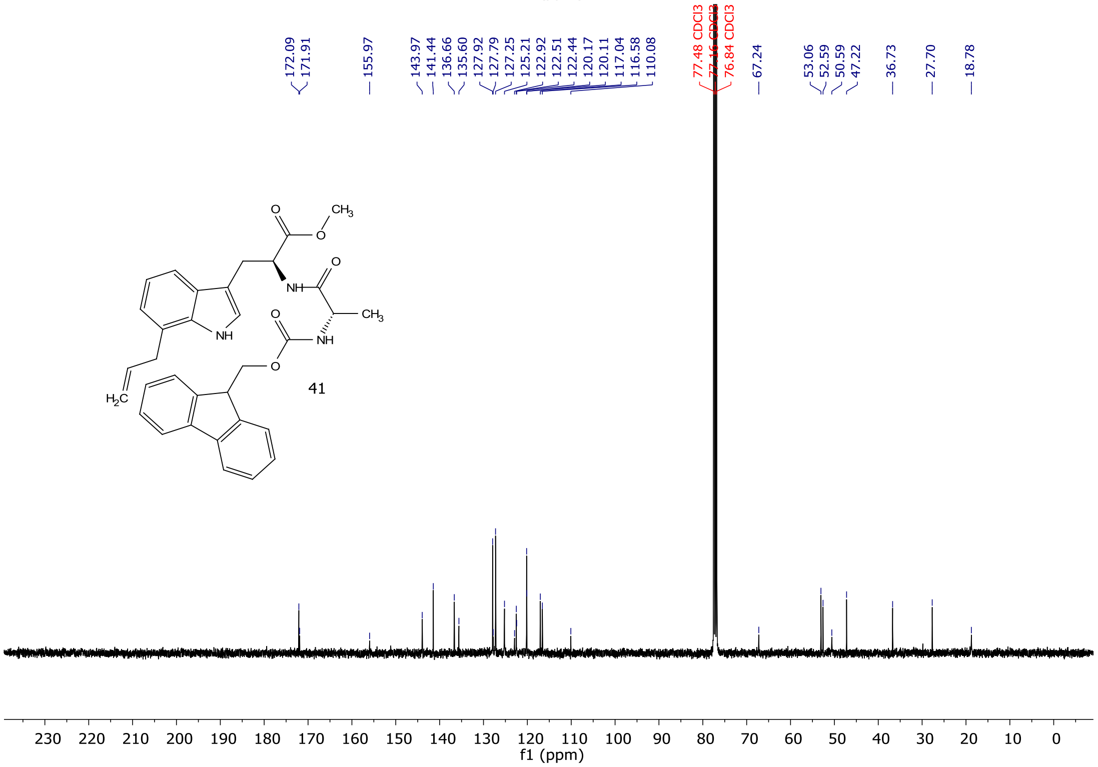

Figure S36: ^{1}H NMR (400 MHz, $CDCl_3$) spectrum (top) and ^{13}C {^{1}H} NMR (101 MHz, $CDCl_3$) spectrum (bottom) for **41**.

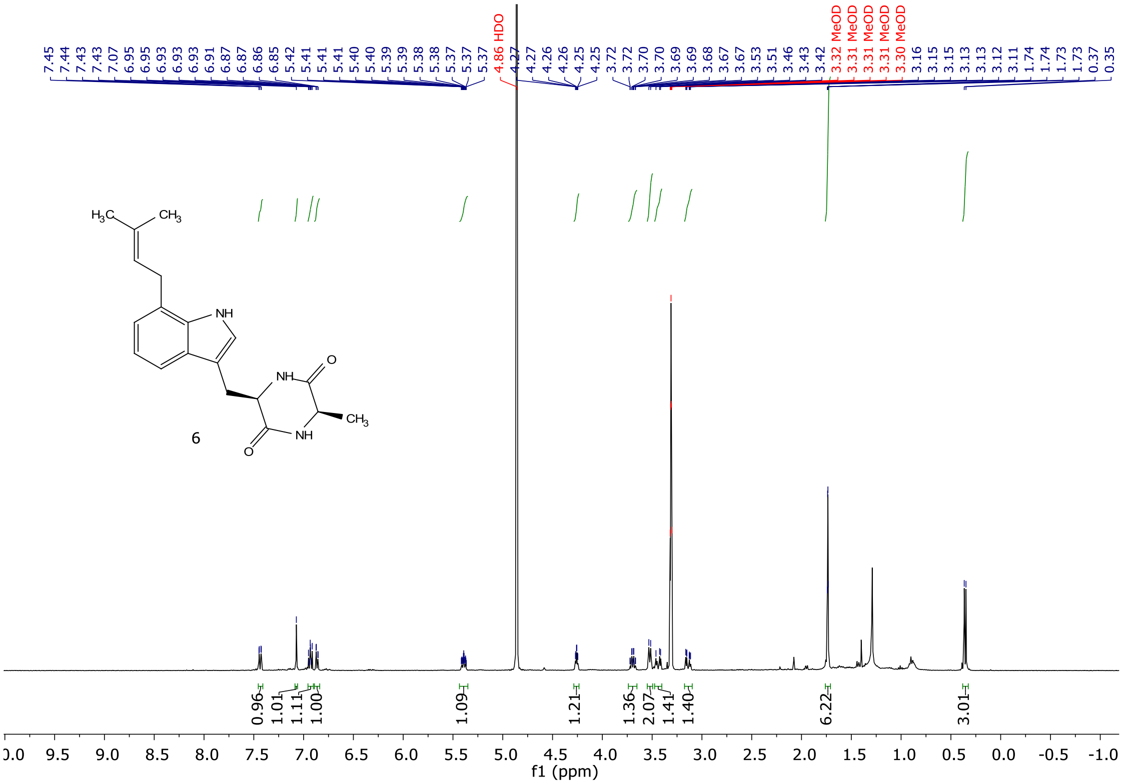

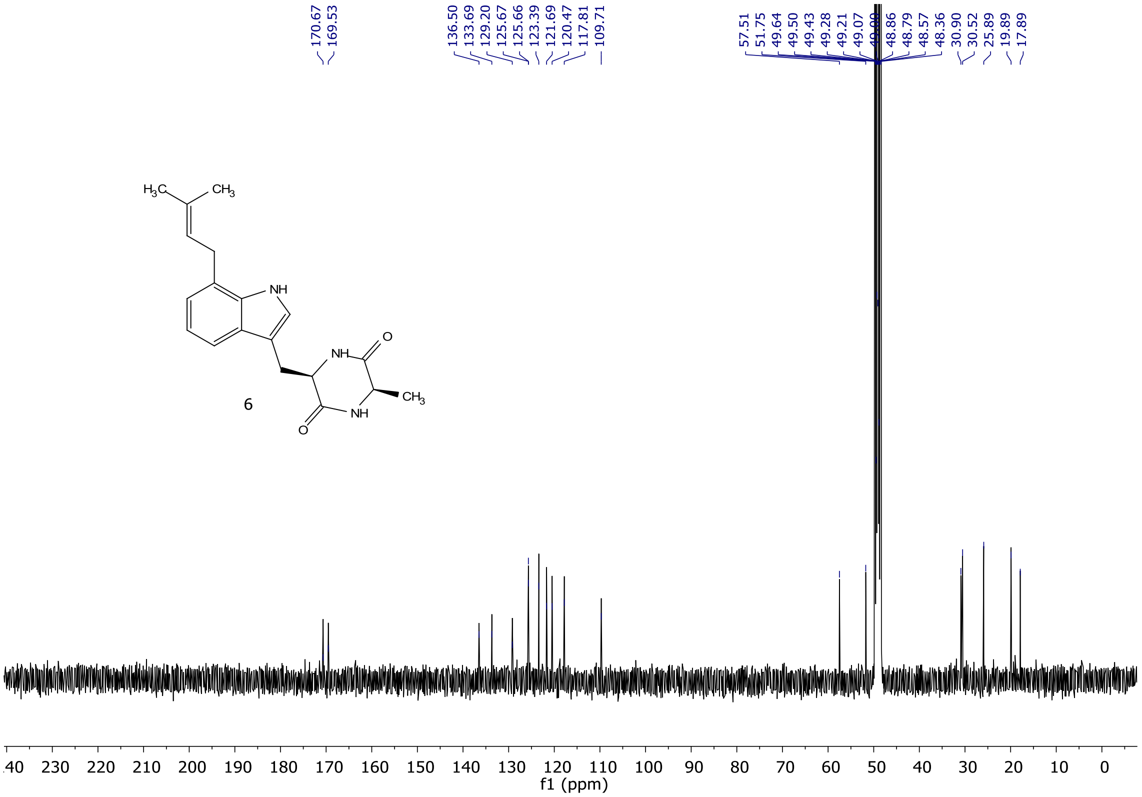

Figure S37: ^{1}H NMR (400 MHz, methanol-d_4) spectrum (top) and ^{13}C {^{1}H} NMR (101 MHz, MeOD) spectrum (bottom) for **6**.

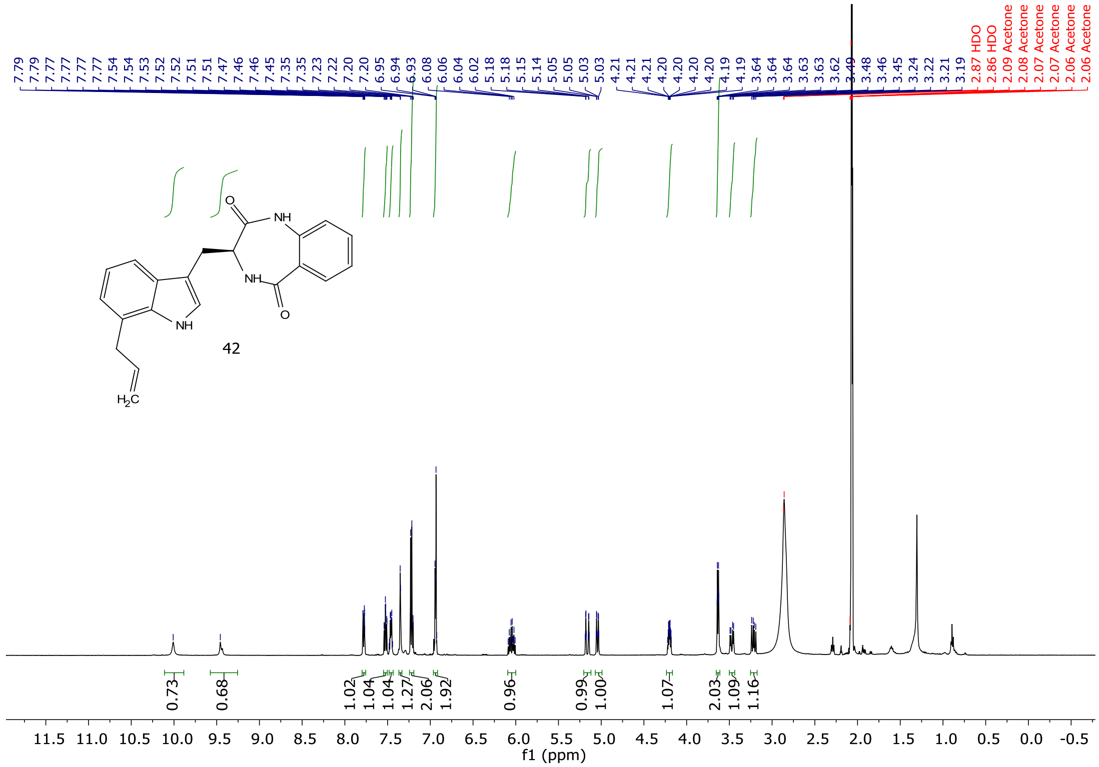

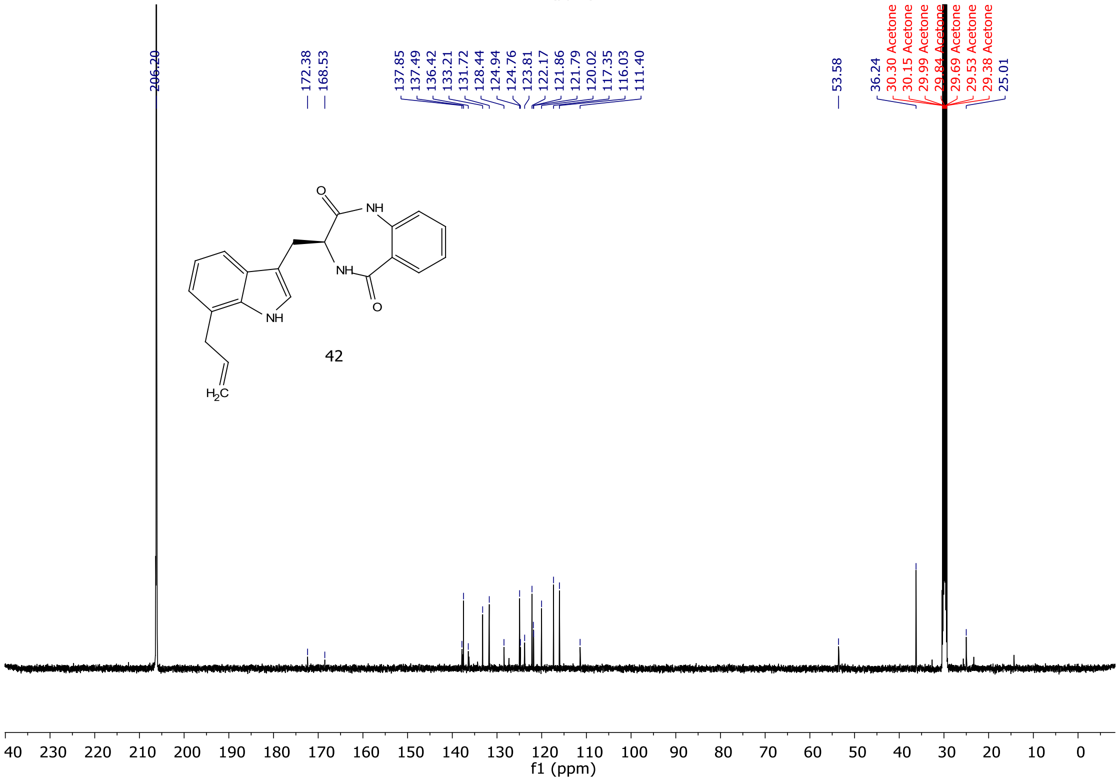

Figure S38: ^{1}H NMR (500 MHz, acetone-d_6) spectrum (top) and ^{13}C {^{1}H} NMR (126 MHz, acetone) spectrum (bottom) for **42**.

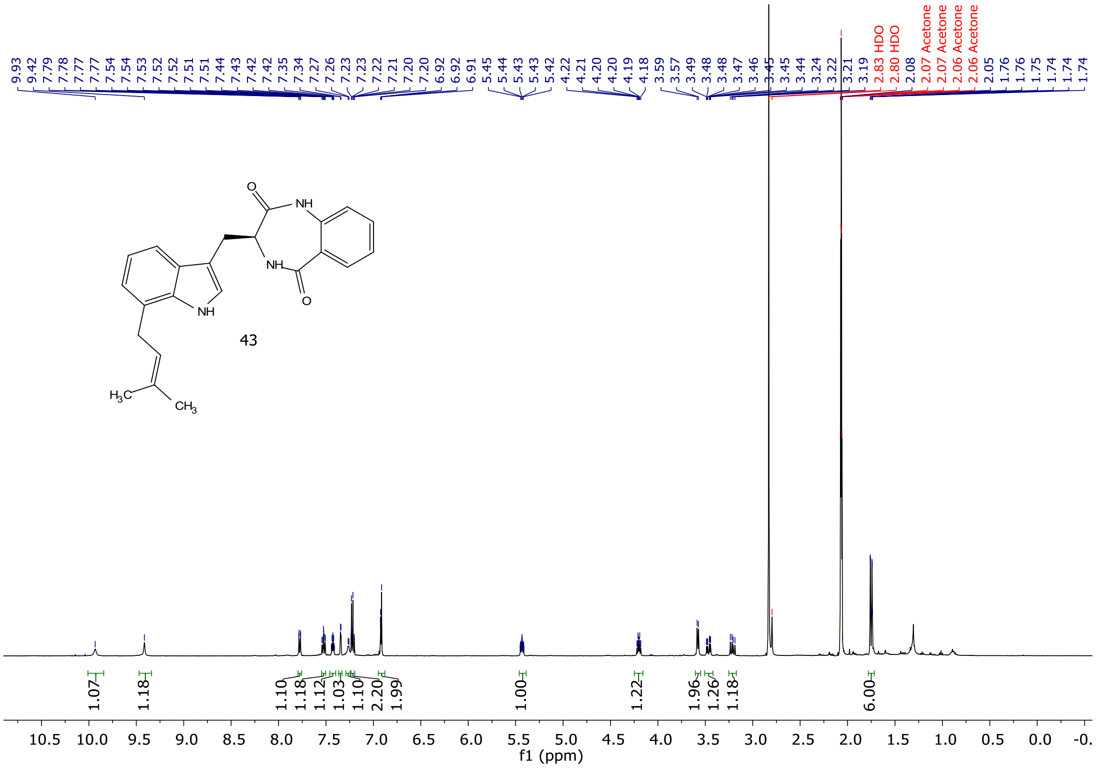

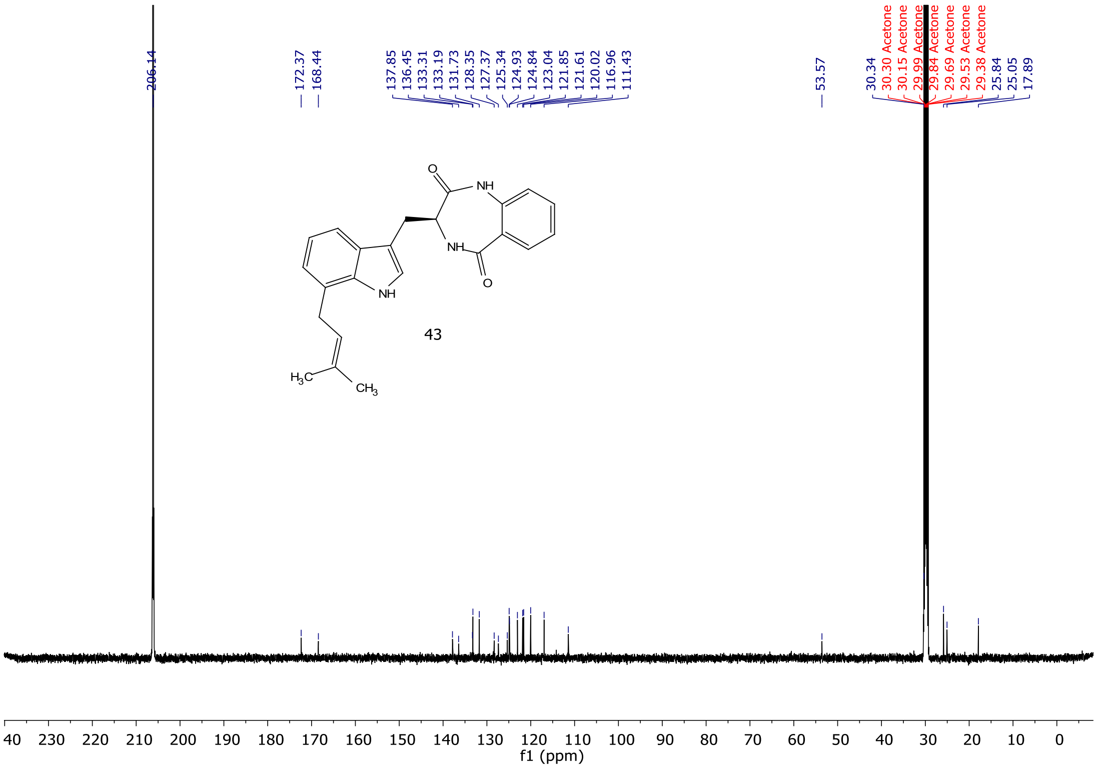

Figure S39: ^{1}H NMR (500 MHz, acetone-d_6) spectrum (top) and ^{13}C {^{1}H} NMR (126 MHz, acetone) spectrum (bottom) for **43**.
